# Cleaning up the 'Bigmessidae': Molecular phylogeny of scleractinian corals from Faviidae, Merulinidae, Pectiniidae and Trachyphylliidae

**DOI:** 10.1186/1471-2148-11-37

**Published:** 2011-02-07

**Authors:** Danwei Huang, Wilfredo Y Licuanan, Andrew H Baird, Hironobu Fukami

**Affiliations:** 1 Scripps Institution of Oceanography, University of California, San Diego, La Jolla, CA 92093, USA; 2 Department of Biological Sciences, National University of Singapore, Singapore 117543, Singapore; 3 Br. Alfred Shields Marine Station and Biology Department, De La Salle University, Manila 1004, The Philippines; 4 ARC Center of Excellence for Coral Reef Studies, James Cook University, Townsville, Queensland 4811, Australia; 5 Department of Marine Biology and Environmental Science, University of Miyazaki, Miyazaki 889-2192, Japan

## Abstract

**Background:**

Molecular phylogenetic studies on scleractinian corals have shown that most taxa are not reflective of their evolutionary histories. Based principally on gross morphology, traditional taxonomy suffers from the lack of well-defined and homologous characters that can sufficiently describe scleractinian diversity. One of the most challenging clades recovered by recent analyses is 'Bigmessidae', an informal grouping that comprises four conventional coral families, Faviidae, Merulinidae, Pectiniidae and Trachyphylliidae, interspersed among one another with no apparent systematic pattern. There is an urgent need for taxonomic revisions in this clade, but it is vital to first establish phylogenetic relationships within the group. In this study, we reconstruct the evolutionary history of 'Bigmessidae' based on five DNA sequence markers gathered from 76 of the 132 currently recognized species collected from five reef regions in the central Indo-Pacific and the Atlantic.

**Results:**

We present a robust molecular phylogeny of 'Bigmessidae' based on the combined five-gene data, achieving a higher degree of resolution compared to previous analyses. Two Pacific species presumed to be in 'Bigmessidae' are more closely related to outgroup clades, suggesting that other unsampled taxa have unforeseen affinities. As expected, nested within 'Bigmessidae' are four conventional families as listed above, and relationships among them generally corroborate previous molecular analyses. Our more resolved phylogeny supports a close association of *Hydnophora *(Merulinidae) with *Favites *+ *Montastraea *(Faviidae), rather than with the rest of Merulinidae, i.e., *Merulina *and *Scapophyllia*. *Montastraea annularis*, the only Atlantic 'Bigmessidae' is sister to *Cyphastrea*, a grouping that can be reconciled by their septothecal walls, a microstructural feature of the skeleton determined by recent morphological work. Characters at the subcorallite scale appear to be appropriate synapomorphies for other subclades, which cannot be explained using macromorphology. Indeed, wide geographic sampling here has revealed more instances of possible cryptic taxa confused by evolutionary convergence of gross coral morphology.

**Conclusions:**

Numerous examples of cryptic taxa determined in this study support the assertion that diversity estimates of scleractinian corals are erroneous. Fortunately, the recovery of most 'Bigmessidae' genera with only minor degrees of paraphyly offers some hope for impending taxonomic amendments. Subclades are well defined and supported by subcorallite morphological features, providing a robust framework for further systematic work.

## Background

For the last two decades, coral systematists have been untangling the complex evolutionary relationships among scleractinian species using DNA sequence data. Seminal molecular phylogenetic work by Romano and Palumbi [1,2] divided the Scleractinia into two major clades, the robust and complex groups, and indicated many problems with traditional taxonomy based on morphology (see also [3]). For instance, *Leptastrea *was recovered within a Fungiina clade rather than the suborder Faviina, where morphological studies had placed it (e.g., [4,5]). Gradually, using more genetic loci, further evidence was uncovered to show that non-monophyly of coral taxa is widespread in Scleractinia (e.g., [6-11]). This culminated in a comprehensive survey of the entire taxon by Fukami et al. [12], which showed that while Scleractinia is monophyletic, most taxonomic groups within it are not. In fact, a staggering 11 of 16 conventional families are polyphyletic.

Undoubtedly, one of the most challenging clades that have been recovered by recent analyses is a group of robust corals in clade XVII [12]. The disarray within the clade is epitomized by its informal name 'Bigmessidae' [13,14]. This clade contains species from four traditional coral families, Faviidae, Merulinidae, Pectiniidae and Trachyphylliidae, interspersed among one another in the tree based on mitochondrial cytochrome oxidase I (COI) and cytochrome b gene sequences [12]. With the exception of the *Montastraea annularis *complex, all members of this clade are from the Indo-Pacific. Families with all species included within clade XVII are Trachyphylliidae (monospecific) and Merulinidae, the latter being polyphyletic, while Faviidae and Pectiniidae have representatives present within and outside clade XVII. Although the clade has not been examined in detail, Huang et al. [15] showed that representatives from other families (Merulinidae and Mussidae) are also nested within it, and several genera are not monophyletic (i.e., *Echinopora*, *Favia*, *Favites, Goniastrea and Montastraea*). In addition, Fukami et al. [12] found para- or polyphyly in *Leptoria*, *Oulophyllia *and *Platygyra *for at least one marker.

Clearly, there exists an urgent need for taxonomic revisions in this clade, amidst the ongoing disarray in the Scleractinia. But in order to begin any form of revision for clade XVII, it is first necessary to determine which subclades are problematic, using as complete a morphological and genetic coverage as possible. Up to this point, the largest number of markers used for analysis of this group has been derived from Fukami et al. [12], who used the aforementioned mitochondrial genes, as well as the nuclear β-tubulin and 28S rDNA separately. However, only 33 species represented by 38 terminals were analyzed for clade XVII, and several subclades were not resolved due to their short branches. Resolution was improved in Huang et al. [15], which included 85 terminals from 43 species, but that study used only COI and a noncoding intergenic mitochondrial region (IGR).

In this study, we present data for five molecular markers—two mitochondrial and three nuclear loci—from 76 of the 132 currently recognized species in clade XVII [12]. We also included seven species from other robust corals as outgroups. Corals were sequenced from five reef regions—the central and northern Great Barrier Reef in Australia, Wakayama in Japan, Batangas in the Philippines, Singapore and the Caribbean. We reconstruct the evolutionary history of clade XVII and identify subclade placement of species that have not been studied in a molecular phylogenetic context. As some species were sampled from multiple locations, we also test if these corals were as widespread as previously recorded.

## Methods

### Specimen collection and DNA extraction

Specimens were collected from coral reefs in five regions—Singapore, Wakayama (Japan), Queensland (Australia), Batangas (The Philippines), and the Caribbean. To ensure consistency in identifications among localities, each coral was sampled by at least two authors, based on morphological features that can be recognized in the field. The identity was later confirmed in the laboratory after examining skeletal traits [5,16-21]. In total, 124 specimens from 83 species in clades XIV-XXI have been included in the present analysis (Table 1; see Additional file 1). We photographed each colony in the field and collected between 10 and 100 cm^2 ^of coral from each colony using a hammer and chisel, with ~2cm^2 ^of tissue preserved in 100% ethanol.

**Table 1:** Species and DNA sequences examined in this study.

No.	Species	Voucher	28S rDNA	histone H3	ITS rDNA	mt COI	mt IGR
1	*Acanthastrea echinata *(XX; Mussidae)	S031	HQ203399	HQ203520	HQ203308	EU371658	
2	*Barabattoia amicorum*	S047	HQ203400	HQ203521	HQ203309	FJ345412	FJ345480
3	*Caulastraea echinulata*	S041	HQ203401	HQ203522		FJ345414	FJ345496
4	*Caulastraea furcata*	P108	HQ203402	HQ203523		HQ203248	HQ203639
5	***Caulastraea tumida***	G61875	HQ203403	HQ203524	HQ203310	HQ203249	HQ203640
6	*Cyphastrea chalcidicum*	G61902	HQ203404	HQ203525	HQ203311	HQ203250	
7	*Cyphastrea chalcidicum*	S103	HQ203405	HQ203526	HQ203312	FJ345415	
8	*Cyphastrea microphthalma*	S069	HQ203406	HQ203527		FJ345416	
9	*Cyphastrea serailia*	G61889	HQ203407	HQ203528	HQ203313	HQ203251	
10	*Cyphastrea serailia*	S024	HQ203408	HQ203529	HQ203314	EU371659	
11	*Cyphastrea serailia*	P120	HQ203409	HQ203530		HQ203252	
12	*Diploastrea heliopora *(XV)	S048	HQ203410	HQ203531	HQ203315	EU371660	
13	*Echinopora gemmacea*	S120	HQ203411	HQ203532	HQ203316	FJ345418	FJ345457
14	***Echinopora horrida***	G61907	HQ203412	HQ203533	HQ203317	HQ203253	HQ203641
15	*Echinopora lamellosa*	S109	HQ203413	HQ203534	HQ203318	FJ345419	FJ345458
16	***Echinopora mammiformis***	G61884	HQ203414	HQ203535	HQ203319	HQ203254	HQ203642
17	*Echinopora pacificus*	S110	HQ203415	HQ203536	HQ203320	FJ345420	FJ345459
18	*Favia danae*	G61885	HQ203416	HQ203537	HQ203321		HQ203643
19	*Favia danae*	S092	HQ203417	HQ203538		EU371663	FJ345476
20	*Favia favus*	G61880	HQ203418	HQ203539	HQ203322	HQ203255	HQ203644
21	*Favia favus*	G61915	HQ203419	HQ203540	HQ203323	HQ203256	HQ203645
22	*Favia favus*	S003	HQ203420	HQ203541	HQ203324	EU371710	FJ345511
23	*Favia favus*	S025	HQ203421	HQ203542		EU371664	FJ345465
24	*Favia favus*	S040	HQ203422	HQ203543	HQ203325	EU371665	FJ345466
25	*Favia favus*	P105	HQ203423	HQ203544		HQ203257	HQ203646
26	*Favia fragum *(XXI)		AF549222			AB117222	
27	*Favia *cf. *laxa*	S013	HQ203424	HQ203545		EU371707	FJ345508
28	*Favia *cf. *laxa*	S014	HQ203425	HQ203546	HQ203326	EU371708	FJ345509
29	*Favia lizardensis*	G61872	HQ203426	HQ203547	HQ203327		HQ203647
30	*Favia lizardensis*	S072	HQ203427	HQ203548	HQ203328	EU371668	FJ345484
31	*Favia lizardensis*	P136	HQ203428	HQ203549			HQ203648
32	***Favia *cf. *maritima***	G61912	HQ203429	HQ203550	HQ203329	HQ203258	HQ203649
33	*Favia matthaii*	G61881	HQ203430	HQ203551	HQ203330		
34	*Favia matthaii*	G61883	HQ203431	HQ203552	HQ203331	HQ203259	HQ203650
35	*Favia matthaii*	S005	HQ203432	HQ203553	HQ203332	EU371669	FJ345471
36	*Favia matthaii*	S029	HQ203433	HQ203554	HQ203333	EU371671	FJ345473
37	*Favia maxima*	S052	HQ203434	HQ203555	HQ203334	EU371674	
38	*Favia maxima*	P142	HQ203435	HQ203556		HQ203260	HQ203651
39	*Favia *cf. *maxima*	P134	HQ203436	HQ203557	HQ203335	HQ203261	HQ203652
40	*Favia pallida*	G61898	HQ203437	HQ203558	HQ203336		HQ203653
41	*Favia pallida*	S036	HQ203438	HQ203559	HQ203337	EU371675	FJ345482
42	***Favia rosaria***	G61911	HQ203439	HQ203560	HQ203338	HQ203262	HQ203654
43	*Favia rotumana*	S068	HQ203440	HQ203561	HQ203339	FJ345427	FJ345485
44	***Favia rotundata***	G61874	HQ203441	HQ203562	HQ203340	HQ203263	
45	***Favia rotundata***	P132	HQ203442	HQ203563			
46	*Favia speciosa*	S001	HQ203443	HQ203564	HQ203341	EU371677	FJ345505
47	*Favia speciosa*	S026	HQ203444	HQ203565		EU371680	FJ345506
48	*Favia speciosa*	P103	HQ203445	HQ203566	HQ203342	HQ203264	HQ203655
49	*Favia stelligera*	P141	HQ203446	HQ203567	HQ203343	HQ203265	HQ203656
50	***Favia truncatus***	G61897	HQ203447	HQ203568	HQ203344	HQ203266	HQ203657
51	***Favites abdita***	S002	HQ203448	HQ203569	HQ203345	HQ203267	
52	*Favites chinensis*	S084	HQ203449	HQ203570	HQ203346	HQ203268	
53	*Favites complanata*	S007	HQ203450	HQ203571	HQ203347	EU371689	
54	***Favites flexuosa***	P116	HQ203451	HQ203572	HQ203348	HQ203269	
55	*Favites halicora*	S115	HQ203452	HQ203573	HQ203349	HQ203270	
56	*Favites paraflexuosa*	S100	HQ203453	HQ203574	HQ203350	EU371694	FJ345521
57	*Favites pentagona*	S086	HQ203454	HQ203575	HQ203351	EU371695	
58	*Favites pentagona*	P111	HQ203455	HQ203576		HQ203271	
59	***Favites russelli***	G61895	HQ203456	HQ203577	HQ203352	HQ203272	HQ203658
60	***Favites stylifera***	P128	HQ203457	HQ203578	HQ203353	HQ203273	HQ203659
61	*Goniastrea aspera*	S107	HQ203458	HQ203579	HQ203354	FJ345430	FJ345487
62	*Goniastrea australensis*	G61876	HQ203459	HQ203580	HQ203355	HQ203274	HQ203660
63	*Goniastrea australensis*	S088	HQ203460	HQ203581	HQ203356	FJ345431	FJ345490
64	*Goniastrea australensis*	S098	HQ203461	HQ203582		EU371696	FJ345491
65	*Goniastrea edwardsi*	S045	HQ203462	HQ203583	HQ203357	EU371697	FJ345492
66	*Goniastrea edwardsi*	S117	HQ203463	HQ203584		FJ345432	FJ345493
67	*Goniastrea favulus*	G61877	HQ203464	HQ203585	HQ203358		HQ203661
68	*Goniastrea favulus*	S022	HQ203465	HQ203586		EU371698	FJ345494
69	*Goniastrea palauensis*	S021	HQ203466	HQ203587	HQ203359	EU371699	FJ345488
70	*Goniastrea pectinata*	G61879	HQ203467	HQ203588	HQ203360		HQ203662
71	*Goniastrea pectinata*	S043	HQ203468	HQ203589		FJ345434	FJ345489
72	*Goniastrea pectinata*	P110	HQ203469	HQ203590			HQ203663
73	*Goniastrea retiformis*	S083	HQ203470	HQ203591	HQ203361	EU371700	FJ345527
74	*Goniastrea retiformis*	P119	HQ203471	HQ203592		HQ203275	HQ203664
75	*Hydnophora exesa *(Merulinidae)	P127	HQ203472	HQ203593	HQ203362	HQ203276	HQ203665
76	***Hydnophora microconos ***(Merulinidae)	P121	HQ203473	HQ203594	HQ203363	HQ203277	HQ203666
77	***Hydnophora pilosa*** (Merulinidae)	P138	HQ203474	HQ203595	HQ203364	HQ203278	HQ203667
78	*Leptoria irregularis*	P133	HQ203475	HQ203596		HQ203279	HQ203668
79	*Leptoria phrygia*	S081	HQ203476	HQ203597	HQ203365	EU371705	FJ345529
80	*Lobophyllia corymbosa *(XIX; Mussidae)		AF549237			AB117241	
81	*Merulina ampliata *(Merulinidae)	P106	HQ203477	HQ203598		HQ203280	HQ203669
82	*Merulina scabricula *(Merulinidae)	P114	HQ203478	HQ203599	HQ203366	HQ203281	HQ203670
83	*Montastraea annularis*	A622	HQ203479	HQ203600	HQ203367	HQ203282	
84	*Montastraea *cf. *annuligera*	P117	HQ203481	HQ203602	HQ203369		HQ203671
85	*Montastraea cavernosa *(XVI)	A005	HQ203480	HQ203601	HQ203368	HQ203283	
86	***Montastraea colemani***	P118	HQ203482	HQ203603		HQ203284	
87	*Montastraea curta*	G61882	HQ203483	HQ203604	HQ203370	HQ203285	
88	*Montastraea curta*	P122	HQ203484	HQ203605		HQ203286	
89	*Montastraea magnistellata*	G61896	HQ203485	HQ203606	HQ203371	HQ203287	
90	*Montastraea magnistellata*	P109	HQ203486	HQ203607		HQ203288	
91	***Montastraea multipunctata***	P131	HQ203487	HQ203608	HQ203372	HQ203289	
92	***Montastraea salebrosa***	P139	HQ203488	HQ203609	HQ203373	HQ203290	HQ203672
93	*Montastraea valenciennesi*	G61904	HQ203489	HQ203610		HQ203291	HQ203673
94	*Montastraea valenciennesi*	S006	HQ203490	HQ203611	HQ203374	EU371713	FJ345514
95	*Montastraea valenciennesi*	S008	HQ203491	HQ203612		EU371714	FJ345515
96	*Montastraea valenciennesi*	P102	HQ203492	HQ203613	HQ203375	HQ203292	
97	***Moseleya latistellata***	G61909	HQ203493	HQ203614	HQ203376	HQ203293	HQ203674
98	*Mussa angulosa *(XXI; Mussidae)		AF549236		AB441402	NC_008163	
99	*Mycedium elephantotus *(Pectiniidae)	S121	HQ203494	HQ203615	HQ203377	HQ203294	HQ203675
100	***Mycedium robokaki ***(Pectiniidae)	S126	HQ203495	HQ203616	HQ203378	HQ203295	HQ203676
101	*Oulophyllia bennettae*	G61873	HQ203496	HQ203617		HQ203296	HQ203677
102	*Oulophyllia bennettae*	S033	HQ203497	HQ203618	HQ203379	FJ345436	FJ345497
103	*Oulophyllia *aff. *bennettae*	P140	HQ203498	HQ203619	HQ203380	HQ203297	
104	*Oulophyllia crispa*	S055	HQ203499	HQ203620	HQ203381	EU371721	FJ345500
105	*Pectinia alcicornis *(Pectiniidae)	P124	HQ203500	HQ203621	HQ203382	HQ203298	HQ203678
106	***Pectinia ayleni*** (Pectiniidae)	S122	HQ203501	HQ203622	HQ203383	HQ203299	HQ203679
107	***Pectinia lactuca ***(Pectiniidae)	P115	HQ203502	HQ203623	HQ203384	HQ203300	HQ203680
108	*Pectinia paeonia *(Pectiniidae)	P126	HQ203503	HQ203624	HQ203385	HQ203301	HQ203681
109	***Platygyra acuta***	P123	HQ203504	HQ203625	HQ203386		HQ203682
110	***Platygyra contorta***	P112	HQ203505	HQ203626	HQ203387		HQ203683
111	*Platygyra daedalea*	G61878	HQ203506	HQ203627			HQ203684
112	*Platygyra daedalea*	S116	HQ203507	HQ203628	HQ203388	FJ345440	FJ345530
113	*Platygyra lamellina*	G61887	HQ203508	HQ203629	HQ203389	HQ203302	HQ203685
114	*Platygyra lamellina*	S114	HQ203509	HQ203630		FJ345441	FJ345531
115	*Platygyra pini*	G61899	HQ203510	HQ203631	HQ203390	HQ203303	HQ203686
116	*Platygyra pini*	S035	HQ203511	HQ203632	HQ203391	FJ345443	FJ345535
117	***Platygyra ryukyuensis***	P101	HQ203512	HQ203633	HQ203392	HQ203304	HQ203687
118	*Platygyra sinensis*	S118	HQ203513	HQ203634	HQ203393	FJ345442	FJ345534
119	*Platygyra sinensis*	P130	HQ203514	HQ203635		HQ203305	HQ203688
120	*Platygyra *cf. *verweyi*	S037	HQ203515	HQ203636	HQ203394	EU371722	FJ345532
121	*Plesiastrea versipora *(XIV)	S127	HQ203397	HQ203518	HQ203307	HQ203246	
122	*Plesiastrea versipora *(XIV)	P137	HQ203398	HQ203519		HQ203247	
123	*Scapophyllia cylindrica *(Merulinidae)	S060	HQ203516	HQ203637	HQ203395	FJ345444	FJ345502
124	*Trachyphyllia geoffroyi *(Trachyphylliidae)	J001	HQ203517	HQ203638	HQ203396	HQ203306	HQ203689

Unless indicated by roman numerals and/or family names in parentheses, all species belong to clade XVII and Faviidae, respectively. Species placed in a molecular phylogenetic context for the first time are in bold. Specimens with voucher numbers starting with 'G' are from Great Barrier Reef (Australia), 'S' from Singapore, 'J' from Japan, 'P' from the Philippines, and 'A' from the Atlantic. GenBank accession numbers are displayed for each molecular marker.

For each colony from Singapore, Japan and the Caribbean, DNA was extracted from ~2 cm^2 ^of tissue digested in twice their volume of CHAOS solution (not an acronym; 4 M guanidine thiocyanate, 0.1% N-lauroyl sarcosine sodium, 10 mM Tris pH 8, 0.1 M 2-mercaptoethanol) for at least three days at room temperature before DNA extraction using a phenol-chloroform based method with a phenol extraction buffer (100 mM TrisCl pH 8, 10 mM EDTA, 0.1% SDS) [15,22-24]. For specimens from Australia and the Philippines, genomic DNA was extracted from the tissues preserved in ethanol using the Qiagen DNeasy kit, following the manufacturer's instructions.

The rest of the colony was sprayed with a powerful water jet to remove as much tissue as possible before being bleached in 5-10% sodium hypochlorite solution. The skeletons were rinsed in fresh water, dried, and deposited in the Raffles Museum of Biodiversity Research (Singapore), Seto Marine Biological Laboratory (Wakayama, Japan), Museum of Tropical Queensland (Australia), and De La Salle University (Manila, The Philippines) (Table 1).

### PCR amplification and sequencing

A total of five molecular markers were amplified for a majority of the samples (Tables 1 and 2). They consist of three nuclear and two mitochondrial loci: (1) 28S rDNA D1 and D2 fragments; (2) histone H3; (3) internal transcribed spacers 1 and 2, including 5.8S rDNA (ITS in short); (4) cytochrome oxidase subunit I (COI); and (5) noncoding intergenic region situated between COI and the formylmethionine transfer RNA gene (IGR in short) [8,23,25-27].

**Table 2:** Molecular markers utilized for phylogenetic reconstruction.

Marker	Primer pairs	Total characters (informative)	Model	Source
28S rDNA	C1': 5'-ACC CGC TGA ATT TAA GCA T-3' D2MAD: 5'-GAC GAT CGA TTT GCA CGT CA-3'	861 (135)	HKY+Γ	[25]
histone H3	H3F: 5'-ATG GCT CGT ACC AAG CAG ACV GC-3' H3R: 5'-ATA TCC TTR GGC ATR ATR GTG AC-3'	374 (73)	HKY+Γ	[26]
ITS rDNA	A18S: 5'-GATCGAACGGTTTAGTGAGG-3' ITS-4: 5'-TCCTCCGCTTATTGATATGC-3'	1137 (425)	SYM+Γ	[27]
mt COI	MCOIF: 5'-TCTACAAATCATAAAGACATAGG-3' MCOIR: 5'-GAGAAATTATACCAAAACCAGG-3'	719 (71)	HKY+I	[8]
mt IGR	MNC1f: 5'-GAGCTGGGCTTCTTTAGAGTG-3' MNC1r: 5'-GTGAGACTCGAACTCACTTTTC-3'	1509 (763)	SYM+I	[23]

The mitochondrial intergenic region (IGR) was too variable to be aligned across the entire clade, so only alignable sequences were included in the analysis. ITS comprises multiple copies in the nuclear genome, but the primers we used have shown high fidelity for a single copy, precluding the need to clone the amplicons [27-33]. Nevertheless, in the unlikely case that paralogs were sequenced, our analyses could be confused by incomplete lineage sorting [7]. We therefore sequenced the ITS locus from at most one representative of each species, unless analyses of the other four markers did not recover its sequences as a clade. In the latter case, sequences may actually belong to separate cryptic species that have been obscured by gross morphological similarities. For COI, not all specimens of each species were necessarily sequenced since intraspecific variation of this gene is limited [15,24].

PCR products were purified with ExoSAP-IT (GE Healthcare, Uppsala, Sweden) and sequencing was performed by Advanced Studies in Genomics, Proteomics and Bioinformatics (ASGPB) at the University of Hawaii at Manoa using the Applied Biosystems BigDye Terminator kit and an ABI 3730XL sequencer. New sequences were deposited in GenBank under accession numbers HQ203246-HQ203689 (Table 1).

### Phylogenetic analyses

Sequences were organized into five separate data matrices using Mesquite 2.72 [34], and each aligned with the accurate alignment option (E-INS-i) in MAFFT 6.7 [35-37] under default parameters. Substitution saturation of protein-coding genes was assessed via DAMBE [38,39], where we found histone H3 and COI to be unsaturated at the third codon positions for tree inference. Consequently, we concatenated the five gene matrices into a single partitioned matrix consisting of 4600 characters, 1467 of which were parsimony informative. This was analyzed using maximum parsimony, Bayesian likelihood, and maximum likelihood methods. We also carried out these analyses on a four-gene dataset omitting the ITS partition to determine if the phylogenetic reconstruction was sensitive to the ITS sampling strategy.

Under a maximum parsimony framework, we utilized new search technologies [40,41] in the software TNT 1.1 [42,43]. Tree searches consisted of 50000 random addition sequence replicates under the default sectorial, ratchet, drift and tree fusing parameters. Gaps were treated as missing data and clade stability was inferred using 1000 bootstrap replicates each employing 100 random addition sequences.

For maximum likelihood, neighbor-joining and Bayesian analyses, we determined the most suitable model of molecular evolution for each gene partition and the concatenated matrix using jModelTest 0.1.1 [44,45] to test for a total of 24 models, following the Akaike Information Criterion (AIC). The maximum likelihood tree for each partition and the combined dataset was inferred using RAxML 7.2.3 [46,47] at the Cyberinfrastructure for Phylogenetic Research (CIPRES; http://www.phylo.org), employing the GTRGAMMA model. The proportion of invariable sites and gamma distribution shape parameter for variable sites were estimated during the maximum likelihood analysis. Multiparametric bootstrap analysis was carried out using 1000 bootstrap replicates. Maximum likelihood analysis was also carried out with PhyML 3.0 [45] on the combined data, utilizing the AIC-chosen model (GTR+I+Γ), and generating 1000 bootstrap replicates. The neighbor-joining tree of the combined data was calculated in PAUP*4.0b10 [48] with 1000 bootstrap replicates, employing the evolutionary model selected above.

Bayesian inference was carried out in MrBayes 3.1.2 [49,50], using the resources of the Computational Biology Service Unit from Cornell University, with each partition modeled (Table 2) but unlinked for separate parameter estimations. Four Markov chains of 10 million generations were implemented in twelve runs, saving a tree every 100th generation. MCMC convergence among the runs was monitored using Tracer 1.5 [51], where we ascertained that only four of the twelve runs converged on the shortest trees (only two runs converged for the four-gene analysis; see [52-54]), and the first 40001 trees were to be discarded as burn-in.

Additionally, compensatory base changes because of the secondary structure of the ITS rDNA loci may lead to non-independence and increased homoplasy of characters [55-57]. Hence, analysis of the secondary structure of this region may result in a more rigorous phylogeny [58-61]. Using the ITS2 segment of each ITS sequence, secondary structure was predicted by searching the ITS2 database [62] for the best match template and then modeling its structure based on free energy minimization. The ITS2 sequences and their associated structural information were aligned using 4SALE 1.5 [63,64], and then exported for analysis in ProfDistS 0.9.8 [65-68]. The profile neighbor-joining algorithm was executed with 10000 bootstrap replicates on the RNA structural alignment, using the GTR model and rate matrix 'Q_ITS2.txt' for distance correction. ITS2 could not be amplified from *Hydnophora microconos*, *H. pilosa *and *Merulina scabricula*. Consequently these species were excluded from the analysis.

## Results and Discussion

**Figure 1:**
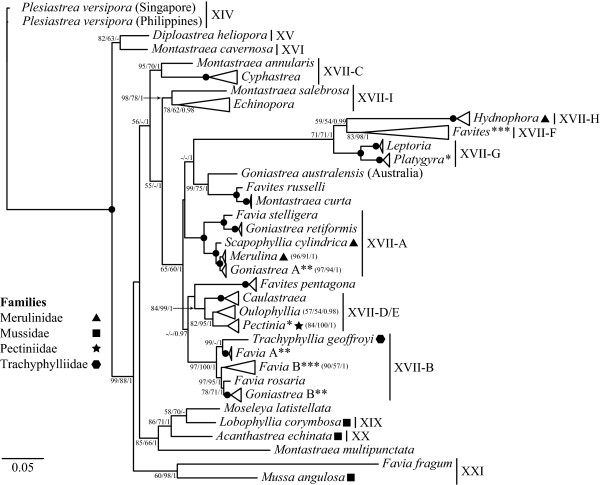
**Maximum likelihood tree of the combined molecular data**. Species have been summarized into genera where possible. One asterisk denotes paraphyletic genus, two asterisks polyphyly, and three represents a genus that is both para- and polyphyletic. All taxa from conventional family Faviidae unless otherwise indicated. Clade designations XIV to XXI shown; clade XVII divided into well-supported subclades. Numbers adjacent to branches/taxa are support values (maximum likelihood bootstrap ≥50, maximum parsimony bootstrap ≥50, followed by Bayesian posterior probability ≥0.9). Filled circles indicate well-supported clades (bootstrap values ≥98 and posterior probability of 1).

**Figure 2:**
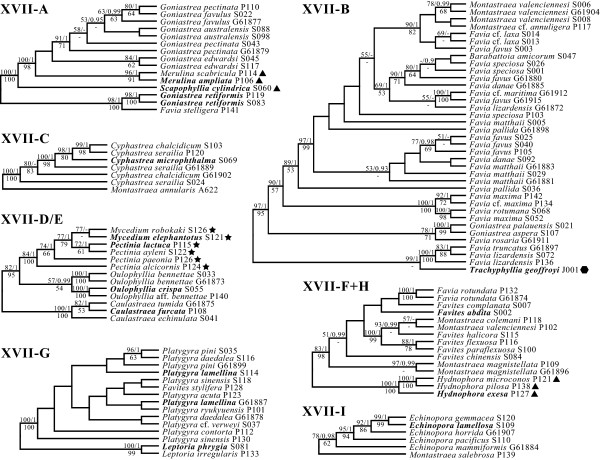
**Maximum likelihood topologies of each subclade**. Numbers above branches are maximum likelihood bootstrap ≥50 and Bayesian posterior probability ≥0.9, while number below denotes maximum parsimony bootstrap ≥50. Family classification follows definitions given for Figure 1. Type species of genera are in bold.

In this study, the evolutionary history of the 'Bigmessidae' corals was robustly reconstructed using five genes. Relations among other clade representatives chosen as outgroups were also inferred. The maximum likelihood reconstructions carried out by RAxML 7.2.3 and PhyML 3.0 had log likelihood values of -36224.67 and -36995.48, respectively. As they were identical when considering nodes with bootstrap values ≥50, we present the RAxML tree that garnered a higher likelihood score (Figures 1 and 2). A total of 182 most parsimonious trees (tree length = 6178) were obtained. No conflicts between tree optimization procedures (including Bayesian inference and the neighbor-joining algorithm) were apparent when considering only the supported nodes (bootstrap ≥50 and posterior probability ≥0.9) (see Additional file 2). Analyses excluding the ITS partition also gave congruent results. Several clades were consistent and well supported among maximum likelihood, parsimony and Bayesian inferences. We named some of these groups within clade XVII from A to I, consistent with the classification in Budd and Stolarski [69]. On the other hand, the neighbor-joining method generated a relatively unresolved tree—subclades A, C, F and I did not achieve bootstrap values of ≥50 (see Additional file 2).

The combined five-gene data yielded the most resolved phylogeny hitherto of clade XVII, with most branches garnering high support values. However, most partitions gave fairly unresolved trees when analyzed individually (see Additional file 3). By examining the support of subclades among trees obtained via different partitions, we found that nuclear markers contributed a greater extent to the final tree topology (Table 3). Histone H3, for instance, supported all higher-level groupings and all subclades except D/E (Figure 1). The 28S and ITS rDNA gene trees had moderate resolution within clade XVII, with only two unresolved subclades each. Surprisingly, the tree based on ITS2 rDNA secondary structure had less resolution than the primary sequence alignment. Indeed, the former has demonstrated potential for resolving intrageneric phylogenies in other anthozoans [70,71], but it is less informative for relationships at higher taxonomic levels [72,73]. Evidently, the COI tree was poorly resolved, with ≥50 bootstrap support for few relationships among major clades and only one subclade. The slow evolution of the mitochondrial COI gene among anthozoans is certainly the reason behind this [24,74,75]. While the intergenic marker (IGR) adjacent to COI on the mitochondrial genome has shown promise for phylogenetic reconstruction among Faviidae and Mussidae [15,23,76], it cannot be unambiguously aligned between the major clades. We urge the development of more nuclear phylogenetic markers that can be reliably applied across diverse scleractinian clades.

**Table 3:** Clades supported by maximum likelihood analysis for each partition.

Clade	Nuclear DNA	mt DNA	28S rDNA	histone H3	ITS sequence	ITS structure	mt COI	mt IGR
XV to XXI	√√	√√	√√	√√	√√	√√	√√	
XV+XVI	√√	X	√√	√√	√	√√	XX	
XVII to XXI	√√	√√	√	√√	√√	√	√√	
XXI	√√	√√	√				√√	
XIX+XX^1^	√√	√	√	√√	X	√√	√	
XVII	√√	X	√	√√	√	X	X	√√
XVII-A	√√	X	√√	√√	√√	X	X	X
XVII-B	√√	X	X	√√	√√	√√	X	√
XVII-C	√√	XX	√√	√√	√√	X	X	
XVII-D/E	√√	XX	X	X	√√	√	XX	√√
XVII-F	√√	X	√√	√√	X	√√	XX	
XVII-G	√√	√√	√√	√√	X	X	√	√√
XVII-H	√√	X	√√	√√	√√		√√	√√
XVII-I^2^	√√	X	√√	√√	√√	√	X	X

^1^*Montastraea multipunctata *and *Moseleya latistellata *are herein considered as part of clade XIX+XX.

^2^Subclade I is expanded to include *Montastraea salebrosa*.

'√√': clade present with ≥50 bootstrap support; '√': clade present but not supported (<50 bootstrap); 'XX': contradicted clade with ≥50 bootstrap support; and 'X': contradicted clade not supported. Empty cells indicate insufficient data.

Most relationships among clades XV to XXI obtained in this study corroborate results of Fukami et al. [12] (Figure 1). The only difference occurs in the sister grouping of *Diploastrea heliopora *(XV) and *Montastraea cavernosa *(XVI) (supported by all analyses except Bayesian likelihood) that form a grade in Fukami et al. [12]. The monophyly of the clade XVII+XIX+XX (Pacific faviids and mussids) is recovered but not well supported. *Montastraea multipunctata *and *Moseleya latistellata *are Pacific faviids, and therefore presumably in clade XVII. But as a result of superficial similarities, they have historically been associated with the Pacific mussids *Blastomussa merleti *(clade XIV) [77] and *Acanthastrea hillae *(clade XVIII) [5,18], respectively. Here, we find them to be more closely related to clades XIX and XX instead, revealing a taxonomic situation more challenging than anticipated. Pacific faviids other than *Diploastrea heliopora *can no longer be restricted to clade XVII, and the possibility exists that yet-to-be sampled taxa provisionally placed in clade XVII—particularly the monotypic genera, *Australogyra*, *Erythrastrea*, *Boninastrea *and *Paraclavarina*—have unexpected affinities.

Nested within clade XVII are four conventional families—Faviidae, Merulinidae, Pectiniidae and Trachyphylliidae (Figure 1). Two Pectiniidae genera, *Pectinia *and *Mycedium *(XVII-E) form the sister clade to *Oulophyllia*. This is a similar relationship to the results of Fukami et al. [12], although here we also show with reasonable support that *Oulophyllia *is monophyletic, and *Caulastraea *is an outgroup rather than nested within *Oulophyllia *(XVII-D). Merulinidae is represented by *Hydnophora*, *Merulina *and *Scapophyllia*. *Hydnophora *is more closely related to *Favites *and Pacific *Montastraea *spp. than *Merulina *and *Scapophyllia*, which form a grade within the clade dominated by *Goniastrea*. The monospecific Trachyphylliidae is nested within the clade consisting primarily of *Favia *spp., and is sister to *Favia lizardensis *and *F. truncatus *(Figure 2). Work is ongoing to redescribe clade XVII by incorporating the above families and applying a new taxon name since the type species of Faviidae, *Favia fragum *(Esper, 1797), belongs to clade XXI [12].

The genetic affiliation of *Hydnophora *and *Trachyphyllia *with Faviidae has previously been proposed by Fukami et al. [8,12]. However, this is not exclusively a molecular hypothesis. Based on a combination of colony, corallite and subcorallite characters (e.g., polyp budding; wall, septal and columellar structures), Vaughan and Wells, 1943 [78], placed the two taxa within Faviidae. But later, Chevalier, 1975 [79], attempted to distinguish *Trachyphyllia *from Faviidae based on minor differences in wall and septal structures by elevating it to the rank of family. Correspondingly, Veron, 1985 [17], moved *Hydnophora *into Merulinidae because of *Hydnophora *species' macromorphological similarities (i.e., colony growth form and polyp structure) with *Merulina ampliata *and *Scapophyllia cylindrica*, which are genetically in the same lineage (subclade A) as several *Goniastrea *spp. and *Favia stelligera *(Figures 1 and 2; see also [8,12]).

*Montastraea annularis *and likely other members of the species complex (*M. faveolata *and *M. franksi*) are the only Atlantic species in clade XVII (see also [8,12]). Most significantly here, *M. annularis *is sister to *Cyphastrea*, forming clade XVII-C (Figure 1). This placement may seem bizarre in the context of traditional macromorphological characters used to classify scleractinians (e.g., [4,78]). However, recent work at the microstructural scale (centers of rapid accretion and thickening deposits) has suggested that their septothecal walls (formed by fusion of outer margins of septa) may unite the two taxa [69] (see also [80]). These subcorallite features appear to be appropriate synapomorphies for other subclades. For instance, clade XVII-A consists of *Merulina*, *Scapophyllia*, *Goniastrea *A and *Favia stelligera *(Figure 2). At the corallite level, these corals cannot be reconciled within the same taxon, since *Favia stelligera *corallites have single centers with separate walls (plocoid), *Goniastrea *spp. have fused walls (cerioid) and may form valleys (meandroid), while *Merulina *and *Scapophyllia *are composed predominantly of elongated valleys (see Additional file 1). On the other hand, they share the apomorphy of having septothecal walls with abortive septa (thin bands between normal septa with their own centers of rapid accretion).

The use of macromorphology for identifying 'Bigmessidae' species is known for being problematic as most of these characters are homoplasious [15,80,81]. The ability to distinguish clades based on microstructural features is encouraging for scleractinian systematics. Micromorphology, at the scale of septal teeth and granules, has also exhibited promise as phylogenetic characters [25,80,82-85]. Interestingly, in light of recent molecular hypotheses, other biological traits, in particular, sexuality and to a lesser extent, breeding mode appear highly conserved and could be further developed as phylogenetic markers [86,87].

Prior to the use of molecular data to build evolutionary trees, it was a great challenge to determine which morphological characters could be useful for classification, given their intraspecific variability [32,88] and phenotypic plasticity [89-94]. Indeed, the general anthozoan body plan is relatively simple, and scleractinians in particular have few discrete morphological characters that are known to be phylogenetically informative at the polyp level [4,95-97]. As a result of the recent disarray in coral systematics, morphological taxonomies of scleractinians have been heavily criticized (e.g., [8,12,98,99]). Molecular characters, which are much more numerous and arguably neutrally evolving, can certainly aid our understanding of evolutionary relationships. However, morphological evidence supporting various molecular clades in the present analysis suggests that morphology at novel scales will play an essential role in the taxonomy of 'Bigmessidae' [80].

Widespread sampling in this study has shown that corals thought to belong to the same species across the central Indo-Pacific are actually from distinct lineages. Consider *Goniastrea australensis *(Milne Edwards and Haime, 1857), which occurs in two clades (Figures 1 and 2; see also Additional file 1). Since this species was first described from Australia, the Australian specimen that clustered with *Favites russelli *and *Montastraea curta *should be considered *G. australensis*, while the two specimens from Singapore (S088 and S098, subclade A) probably represent new species yet to be described. This is certainly not an isolated case. A similar situation is revealed for *Montastraea valenciennesi*. Specimens from Australia (G61904) and Singapore (S006 and S008) are in subclade B of mostly *Favia *spp., while the representative from the Philippines (P102) is in subclade F, a distant clade comprising mainly *Favites *species. Interestingly, two reproductively isolated morphotypes of *M. valenciennesi *were recently found to co-occur in Wakayama (Japan), distinguished by the degree of wall fusion among corallites [100]. Chevalier, 1971 [101], upon examination of the holotype, placed the species in *Favia *on the basis of corallites possessing separate walls and budding intratentacularly (see also [102-108]). This suggests that the name *Favia valenciennesi *(Milne Edwards and Haime, 1848) could be applied to the Australian and Singaporean specimens in subclade B, while P102 (subclade F) is a new species.

Less extensive issues occur among *Goniastrea *and *Favia *species. For instance, *G. pectinata *(subclade A), collected from three locations, is clearly paraphyletic, with *G. australensis *and *G. favulus *nested within them (Figure 2). For *Favia *(subclade B), of six *F. favus *specimens collected from three localities, only three of these form a supported clade while the rest are dispersed within clade XVII-B with no apparent biogeographical pattern. The nesting of *Barabattoia amicorum *among *Favia *spp. has been consistently recovered in recent molecular phylogenies [12,15], but this affinity was in fact the dominant hypothesis [5,107-109] until Veron, 1986 [18], included the species in its current genus. Conversely, *Favia rotundata *clusters with *Favites *spp. rather than its congeners, but it was indeed originally described as *Favites rotundata *Veron, Pichon and Wijsman-Best, 1977 [5] (see also [109,110]).

The polyphyly of most 'Bigmessidae' genera seems to confer a bleak outlook for revisionary work. However, as we have shown in Figure 1, several genera can be clearly grouped as clades with limited name changes. For instance, subclade F is composed of species from *Favites *Link, 1807, *Montastraea *de Blainville, 1830, and *Favia *Ehrenberg, 1834 (Figure 2). While the remaining *Favites *spp. (i.e., *F. pentagona*, *F. russelli*, and *F. stylifera*) are not included within this subclade, the type species of this genus is *Favites abdita *(Ellis and Solander, 1786, type locality 'Probablement les mers des Grandes-Indes', Lamarck, 1816 [111]). The representative of the latter we used falls well within subclade F. Since no other type species were recovered and with *Favites *Link, 1807, being the oldest valid genus in the subclade, *Favites *should be expanded to include the other species, while *F. pentagona*, *F. russelli *and *F. stylifera *will have to be subsumed within other genera. Several other multi-species genera in fact appear stable: *Caulastraea*, *Cyphastrea*, *Echinopora*, *Hydnophora*, *Leptoria*, *Merulina *and *Oulophyllia*. Name changes are certainly not necessary for *Favites *and *Platygyra*, since they host their respective type species in the subclades shown in Figure 2.

## Conclusions

Numerous instances of cryptic taxa determined in this study support the assertion that coral diversity estimates have been fraught with errors [8]. Traits relating to the gross skeletal morphology of corals are unreliable for species description and identification because of their potential for intraspecific variability [32,88] and environment-induced plasticity [89-94]. Yet, these characters have served as the foundation for scleractinian taxonomy (e.g., [4,5]). Fortunately, using molecular data, the recovery of most genera within the 'Bigmessidae' with only minor degrees of paraphyly spells hope for impending taxonomic amendments. Our results show that most genera only require slight revisions, and most major changes are necessary only at the level of the major clades described in Fukami et al. [12]. Certainly, broad taxonomic sampling within Faviidae has revealed more species with unexpected affinities, such as *Moseleya latistellata *and *Montastraea multipunctata*. Clade XVII may consequently have to be redefined to exclude them.

Nevertheless, 'Bigmessidae' subclades are well defined and will no doubt provide a robust framework for taxonomic revisions. The fact that microstructural features support 'Bigmessidae' subclades also offers hope for the morphological approach. Evolutionary relationships among subclades are still provisional due to insufficient statistical support, but they can be clarified with further sampling of nuclear sequences. Eventually, a well-resolved tree of a redescribed clade XVII will be available to reconstruct the morphological evolution of 'Bigmessidae' at various scales.

## Authors' contributions

DH obtained the DNA sequences in the laboratory, performed the phylogenetic analyses, and had a major role in writing the manuscript. All authors collected the specimens examined, contributed to and approved the final manuscript.

## Supplementary Material

Additional file 1 **'Bigmessidae' corals**. Photographs of most coral specimens sequenced in this study. More photographs are available from the authors. Click here for file

Additional file 2 **Maximum likelihood tree topology of the combined molecular data**. Numbers above branches are maximum likelihood bootstrap ≥50 and Bayesian posterior probability ≥0.9, while numbers below denote maximum parsimony bootstrap ≥50 and neighbor-joining bootstrap ≥50. Family classification follows definitions given for Figure 1. Click here for file

Additional file 3 **Maximum likelihood tree topology of each partition**. Numbers adjacent to branches are bootstrap support values ≥50. Definitions for family classification follow Figure 1. Click here for file

## References

[B1] Romano SL Palumbi SR Evolution of scleractinian corals inferred from molecular systematics Science 1996 271 640 642 10.1126/science.271.5249.640

[B2] Romano SL Palumbi SR Molecular evolution of a portion of the mitochondrial 16S ribosomal gene region in scleractinian corals J Mol Evol 1997 45 397 411 10.1007/PL00006245 9321419

[B3] Chen CA Wallace CC Wolstenholme JK Analysis of the mitochondrial 12S rRNA gene supports a two-clade hypothesis of the evolutionary history of scleractinian corals Mol Phylogenet Evol 2002 23 137 149 10.1016/S1055-7903(02)00008-8 12069546

[B4] Wells JW Moore RC Scleractinia Treatise on Invertebrate Paleontology Part F: Coelenterata 1956 Lawrence KS: University of Kansas Press F328 F444

[B5] Veron JEN Pichon M Wijsman-Best M Scleractinia of Eastern Australia. Part II. Families Faviidae, Trachyphylliidae Australian Institute of Marine Science Monograph Series 1977 1 233

[B6] Romano SL Cairns SD Molecular phylogenetic hypotheses for the evolution of scleractinian corals Bull Mar Sci 2000 67 1043 1068

[B7] van Oppen MJH McDonald BJ Willis BL Miller DJ The evolutionary history of the coral genus *Acropora *(Scleractinia, Cnidaria) based on a mitochondrial and a nuclear marker: reticulation, incomplete lineage sorting, or morphological convergence? Mol Biol Evol 2001 18 1315 1329 11420370 10.1093/oxfordjournals.molbev.a003916

[B8] Fukami H Budd AF Paulay G Sole-Cava AM Chen CA Iwao K Knowlton N Conventional taxonomy obscures deep divergence between Pacific and Atlantic corals Nature 2004 427 832 835 10.1038/nature02339 14985760

[B9] Le Goff-Vitry MC Rogers AD Baglow D A deep-sea slant on the molecular phylogeny of the Scleractinia Mol Phylogenet Evol 2004 30 167 177 10.1016/S1055-7903(03)00162-3 15022767

[B10] Kerr AM Molecular and morphological supertree of stony corals (Anthozoa: Scleractinia) using matrix representation parsimony Biol Rev 2005 80 543 558 10.1017/S1464793105006780 16221328

[B11] Benzoni F Stefani F Stolarski J Pichon M Mitta G Galli P Debating phylogenetic relationships of the scleractinian *Psammocora*: molecular and morphological evidences Contrib Zool 2007 76 35 54

[B12] Fukami H Chen CA Budd AF Collins A Wallace CC Chuang YY Dai CF Iwao K Sheppard CRC Knowlton N Mitochondrial and nuclear genes suggest that stony corals are monophyletic but most families of stony corals are not (Order Scleractinia, Class Anthozoa, Phylum Cnidaria) PLoS ONE 2008 3 e3222 10.1371/journal.pone.0003222 18795098 PMC2528942

[B13] Knowlton N Fukami H Chen CA Budd AF Mitochondrial and nuclear genes suggest that stony corals are monophyletic but most families of stony corals are not [abstract] 11th Int Coral Reef Symp 2008 251 10.1371/journal.pone.0003222 PMC2528942 18795098

[B14] Budd AF Systematics and evolution of scleractinian corals Encyclopedia of Life Synthesis Meeting Report 2009 Smithsonian Institution, National Museum of Natural History

[B15] Huang D Meier R Todd PA Chou LM More evidence for pervasive paraphyly in scleractinian corals: Systematic study of Southeast Asian Faviidae (Cnidaria; Scleractinia) based on molecular and morphological data Mol Phylogenet Evol 2009 50 102 116 10.1016/j.ympev.2008.10.012 19000930

[B16] Veron JEN Pichon M Scleractinia of Eastern Australia. Part III. Families Agariciidae, Siderastreidae, Fungiidae, Oculinidae, Merulinidae, Mussidae, Pectiniidae, Caryophylliidae, Dendrophylliidae Australian Institute of Marine Science Monograph Series 1980 1 422

[B17] Veron JEN New Scleractinia from Australian coral reefs Rec West Aust Mus 1985 12 147 183

[B18] Veron JEN Corals of Australia and the Indo-Pacific 1986 Townsville: Australian Institute of Marine Science

[B19] Veron JEN New Scleractinia from Japan and other Indo-West Pacific countries Galaxea 1990 9 95 173

[B20] Veron JEN Corals of the World 2000 Townsville: Australian Institute of Marine Science

[B21] Veron JEN New species described in Corals of the World Australian Institute of Marine Science Monograph Series 2002 1 209

[B22] Sargent TD Jamrich M Dawid IB Cell interactions and the control of gene activity during early development of *Xenopus laevis* Dev Biol 1986 114 238 246 10.1016/0012-1606(86)90399-4 3956863

[B23] Fukami H Budd AF Levitan DR Jara J Kersanach R Knowlton N Geographic differences in species boundaries among members of the *Montastraea annularis *complex based on molecular and morphological markers Evolution 2004 58 324 337 15068349

[B24] Huang D Meier R Todd PA Chou LM Slow mitochondrial COI sequence evolution at the base of the metazoan tree and its implications for DNA barcoding J Mol Evol 2008 66 167 174 10.1007/s00239-008-9069-5 18259800

[B25] Cuif JP Lecointre G Perrin C Tillier A Tillier S Patterns of septal biomineralization in Scleractinia compared with their 28S rRNA phylogeny: a dual approach for a new taxonomic framework Zool Scr 2003 32 459 473 10.1046/j.1463-6409.2003.00133.x

[B26] Colgan DJ McLauchlan A Wilson GDF Livingston SP Edgecombe GD Macaranas J Cassis G Gray MR Histone H3 and U2 snRNA DNA sequences and arthropod molecular evolution Aust J Zool 1998 46 419 437 10.1071/ZO98048

[B27] Takabayashi M Carter DA Loh WKW Hoegh-Guldberg O A coral-specific primer for PCR amplification of the internal transcribed spacer region in ribosomal DNA Mol Ecol 1998 7 928 930

[B28] Takabayashi M Carter DA Ward S Hoegh-Guldberg O Inter- and intra-specific variability in ribosomal DNA sequence in the internal transcribed spacer region of corals Proc Aust Coral Reef Soc 75th Ann Conf 1998 241 248

[B29] Takabayashi M Carter DA Lopez JV Hoegh-Guldberg O Genetic variation of the scleractinian coral *Stylophora pistillata*, from western Pacific reefs Coral Reefs 2003 22 17 22

[B30] van Oppen MJH Worheide G Takabayashi M Nuclear markers in evolutionary and population genetic studies of scleractinian corals and sponges Proc 9th Int Coral Reef Symp 2000 1 131 138

[B31] Lam KKY Morton B Morphological and ITS1, 5.8S, and partial ITS2 ribosomal DNA sequence distinctions between two species *Playtygyra *(Cnidaria: Scleractinia) from Hong Kong Mar Biotechnol 2003 5 555 567 10.1007/s10126-002-0114-x 12913814

[B32] Mangubhai S Souter P Grahn M Phenotypic variation in the coral *Platygyra daedalea *in Kenya: morphometry and genetics Mar Ecol-Prog Ser 2007 345 105 115 10.3354/meps07013

[B33] Knittweis L Kraemer WE Timm J Kochzius M Genetic structure of *Heliofungia actiniformis *(Scleractinia: Fungiidae) populations in the Indo-Malay Archipelago: implications for live coral trade management efforts Conserv Genet 2009 10 241 249 10.1007/s10592-008-9566-5

[B34] Maddison WP Maddison DR Mesquite: a modular system for evolutionary analysis. Version 2.72 http://mesquiteproject.org

[B35] Katoh K Misawa K Kuma K Miyata T MAFFT: a novel method for rapid multiple sequence alignment based on fast Fourier transform Nucleic Acids Res 2002 30 3059 3066 10.1093/nar/gkf436 12136088 PMC135756

[B36] Katoh K Asimenos G Toh H Multiple alignment of DNA sequences with MAFFT Bioinformatics for DNA Sequence Analysis 2009 39 63 full_text 10.1007/978-1-59745-251-9_3 19378139

[B37] Katoh K Toh H Recent developments in the MAFFT multiple sequence alignment program Brief Bioinform 2008 9 286 298 10.1093/bib/bbn013 18372315

[B38] Xia X Data analysis in molecular biology and evolution 2001 Boston: Kluwer Academic Publishers

[B39] Xia X Xie Z DAMBE: Data analysis in molecular biology and evolution J Hered 2001 92 371 373 10.1093/jhered/92.4.371 11535656

[B40] Goloboff PA Analyzing large data sets in reasonable times: Solutions for composite optima Cladistics 1999 15 415 428 10.1111/j.1096-0031.1999.tb00278.x 34902941

[B41] Nixon KC The Parsimony Ratchet, a new method for rapid parsimony analysis Cladistics 1999 15 407 414 10.1111/j.1096-0031.1999.tb00277.x 34902938

[B42] Goloboff PA Farris JS Nixon KC Tree Analysis Using New Technology. Version 1.1 http://www.zmuc.dk/public/phylogeny/TNT

[B43] Goloboff PA Farris JS Nixon KC TNT, a free program for phylogenetic analysis Cladistics 2008 24 774 786 10.1111/j.1096-0031.2008.00217.x

[B44] Posada D jModelTest: Phylogenetic model averaging Mol Biol Evol 2008 25 1253 1256 10.1093/molbev/msn083 18397919

[B45] Guindon S Gascuel O A simple, fast, and accurate algorithm to estimate large phylogenies by maximum likelihood Syst Biol 2003 52 696 704 10.1080/10635150390235520 14530136

[B46] Stamatakis A RAxML-VI-HPC: Maximum likelihood-based phylogenetic analyses with thousands of taxa and mixed models Bioinformatics 2006 22 2688 2690 10.1093/bioinformatics/btl446 16928733

[B47] Stamatakis A Hoover P Rougemont J A rapid bootstrap algorithm for the RAxML web servers Syst Biol 2008 57 758 771 10.1080/10635150802429642 18853362

[B48] Swofford DL PAUP*. Phylogenetic Analysis Using Parsimony (*and Other Methods). Version 4 2003 Sunderland, Massachusetts: Sinauer Associates

[B49] Huelsenbeck JP Ronquist F MRBAYES: Bayesian inference of phylogenetic trees Bioinformatics 2001 17 754 755 10.1093/bioinformatics/17.8.754 11524383

[B50] Ronquist F Huelsenbeck JP MrBayes 3: Bayesian phylogenetic inference under mixed models Bioinformatics 2003 19 1572 1574 10.1093/bioinformatics/btg180 12912839

[B51] Rambaut A Drummond AJ Tracer. Version 1.5 http://beast.bio.ed.ac.uk/Tracer

[B52] Brown JM Hedtke SM Lemmon AR Lemmon EM When trees grow too long: Investigating the causes of highly inaccurate Bayesian branch-length estimates Syst Biol 2010 59 145 161 10.1093/sysbio/syp081 20525627

[B53] Marshall DC Cryptic failure of partitioned Bayesian phylogenetic analyses: Lost in the land of long trees Syst Biol 2010 59 108 117 10.1093/sysbio/syp080 20525623

[B54] Schwartz RS Mueller RL Branch length estimation and divergence dating: estimates of error in Bayesian and maximum likelihood frameworks BMC Evol Biol 2010 10 5 10.1186/1471-2148-10-5 20064267 PMC2827399

[B55] Dixon MT Hillis DM Ribosomal RNA secondary structure: Compensatory mutations and implications for phylogenetic analysis Mol Biol Evol 1993 10 256 267 8450759 10.1093/oxfordjournals.molbev.a039998

[B56] Baldwin BG Sanderson MJ Porter JM Wojciechowski MF Campbell CS Donoghue MJ The ITS region of nuclear ribosomal DNA: A valuable source of evidence on angiosperm phylogeny Ann Missouri Bot Gard 1995 82 247 277 10.2307/2399880

[B57] Álvarez I Wendel JF Ribosomal ITS sequences and plant phylogenetic inference Mol Phylogenet Evol 2003 29 417 434 14615184 10.1016/s1055-7903(03)00208-2

[B58] Müller T Philippi N Dandekar T Schultz J Wolf M Distinguishing species RNA 2007 13 1469 1472 17652131 10.1261/rna.617107 PMC1950759

[B59] Keller A Schleicher T Förster F Ruderisch B Dandekar T Müller T Wolf M ITS2 data corroborate a monophyletic chlorophycean DO-group (Sphaeropleales) BMC Evol Biol 2008 8 218 10.1186/1471-2148-8-218 18655698 PMC2519086

[B60] Coleman AW Is there a molecular key to the level of "biological species" in eukaryotes? A DNA guide Mol Phylogenet Evol 2009 50 197 203 10.1016/j.ympev.2008.10.008 18992828

[B61] Schultz J Wolf M ITS2 sequence-structure analysis in phylogenetics: A how-to manual for molecular systematics Mol Phylogenet Evol 2009 52 520 523 10.1016/j.ympev.2009.01.008 19489124

[B62] Koetschan C Förster F Keller A Schleicher T Ruderisch B Schwarz R Müller T Wolf M Schultz J The ITS2 Database III—sequences and structures for phylogeny Nucleic Acids Res 2010 38 D275 D279 10.1093/nar/gkp966 19920122 PMC2808966

[B63] Seibel PN Müller T Dandekar T Schultz J Wolf M 4SALE-A tool for synchronous RNA sequence and secondary structure alignment and editing BMC Bioinformatics 2006 7 498 10.1186/1471-2105-7-498 17101042 PMC1637121

[B64] Seibel PN Müller T Dandekar T Wolf M Synchronous visual analysis and editing of RNA sequence and secondary structure alignments using 4SALE BMC Res Notes 2008 1 91 10.1186/1756-0500-1-91 18854023 PMC2587473

[B65] Müller T Rahmann S Dandekar T Wolf M Accurate and robust phylogeny estimation based on profile distances: a study of the Chlorophyceae (Chlorophyta) BMC Evol Biol 2004 4 20 15222898 10.1186/1471-2148-4-20 PMC449703

[B66] Friedrich J Dandekar T Wolf M Müller T ProfDist: a tool for the construction of large phylogenetic trees based on profile distances Bioinformatics 2005 21 2108 2109 10.1093/bioinformatics/bti289 15677706

[B67] Rahmann S Müller T Dandekar T Wolf M Hsu HH Efficient and robust analysis of large phylogenetic datasets Advanced Data Mining Technologies in Bioinformatics 2006 Hershey: Idea Group, Inc 104 117

[B68] Wolf M Ruderisch B Dandekar T Schultz J Müller T ProfDistS: (profile-) distance based phylogeny on sequence-structure alignments Bioinformatics 2008 24 2401 2402 10.1093/bioinformatics/btn453 18723521

[B69] Budd AF Stolarski J Corallite wall and septal microstructure in scleractinian reef corals: Comparison of molecular clades within the family Faviidae J Morphol 2011 272 66 88 10.1002/jmor.10899 21061280

[B70] Grajales A Aguilar C Sánchez JA Phylogenetic reconstruction using secondary structures of Internal Transcribed Spacer 2 (ITS2, rDNA): finding the molecular and morphological gap in Caribbean gorgonian corals BMC Evol Biol 2007 7 90 10.1186/1471-2148-7-90 17562014 PMC1913914

[B71] Sánchez JA Dorado D Intragenomic ITS2 variation in Caribbean seafans Proc 11th Int Coral Reef Symp 2008 1383 1387

[B72] Chen CA Chang CC Wei NV Chen CH Lein YT Lin HE Dai CF Callace CC Secondary structure and phylogenetic utility of the ribosomal internal transcribed spacer 2 (ITS2) in scleractinian corals Zool Stud 2004 43 759 771

[B73] Wei NV Wallace CC Dai CF Moothien Pillay LR Chen CA Analyses of the ribosomal internal transcribed spacers (ITS) and the 5.8S gene indicate that extremely high rDNA heterogeneity is a unique feature in the scleractinian coral genus *Acropora *(Scleractinia; Acroporidae) Zool Stud 2006 45 404 418

[B74] Shearer TL van Oppen MJH Romano SL Wörheide G Slow mitochondrial DNA sequence evolution in the Anthozoa (Cnidaria) Mol Ecol 2002 11 2475 2487 10.1046/j.1365-294X.2002.01652.x 12453233

[B75] Hellberg ME No variation and low synonymous substitution rates in coral mtDNA despite high nuclear variation BMC Evol Biol 2006 6 8 10.1186/1471-2148-6-24 16542456 PMC1431588

[B76] Nunes F Fukami H Vollmer SV Norris RD Knowlton N Re-evaluation of the systematics of the endemic corals of Brazil by molecular data Coral Reefs 2008 27 423 432 10.1007/s00338-007-0349-0

[B77] Hodgson G A new species of *Montastrea *(Cnidaria, Scleractinia) from the Philippines Pac Sci 1985 39 283 290

[B78] Vaughan TW Wells JW Revision of the suborders, families, and genera of the Scleractinia Geol Soc Am Spec Pap 1943 44 1 345

[B79] Chevalier JP Les scléractiniaires de la Mélanésie Française (Nouvelle-Calédonie, Iles Chesterfield, Iles Loyauté, Nouvelles Hébrides). Deuxième partie Expéd Française récifs coralliens Nouvelle Calédonie 1975 7 1 407

[B80] Budd AF Stolarski J Searching for new morphological characters in the systematics of scleractinian reef corals: comparison of septal teeth and granules between Atlantic and Pacific Mussidae Acta Zool 2009 90 142 165 10.1111/j.1463-6395.2008.00345.x

[B81] Budd AF Smith ND Diversification of a new Atlantic clade of scleractinian reef corals: insights from phylogenetic analysis of morphologic and molecular data Paleontol Soc Pap 2005 11 103 128

[B82] Stolarski J Roniewicz E Towards a new synthesis of evolutionary relationships and classification of Scleractinia J Paleontol 2001 75 1090 1108 10.1666/0022-3360(2001)075<1090:TANSOE>2.0.CO;2

[B83] Stolarski J Russo A Microstructural diversity of the stylophyllid (Scleractinia) skeleton Acta Palaeontol Pol 2002 47 651 666

[B84] Stolarski J Vertino A First Mesozoic record of the scleractinian *Madrepora *from the Maastrichtian siliceous limestones of Poland Facies 2007 53 67 78 10.1007/s10347-006-0089-6

[B85] Zlatarski VN Need for a more integrative approach to scleractinian taxonomy Proc 11th Int Coral Reef Symp 2008 1406 1410

[B86] Baird AH Guest JR Willis BL Systematic and biogeographical patterns in the reproductive biology of scleractinian corals Annu Rev Ecol Evol Syst 2009 40 551 571 10.1146/annurev.ecolsys.110308.120220

[B87] Kerr AM Baird AH Hughes TP Correlated evolution of sex and reproductive mode in corals (Anthozoa: Scleractinia) Proc R Soc B-Biol Sci 2011 278 75 81 10.1098/rspb.2010.1196 PMC2992726 20659935

[B88] Budd AF Longterm patterns of morphological variation within and among species of reef-corals and their relationship to sexual reproduction Syst Bot 1990 15 150 165 10.2307/2419024

[B89] Budd AF Phenotypic plasticity in the reef corals *Montastraea annularis *(Ellis & Solander) and *Siderastrea siderea *(Ellis & Solander) J Exp Mar Biol Ecol 1979 39 25 54 10.1016/0022-0981(79)90003-0

[B90] Budd AF Large-scale evolutionary patterns in the reef-coral *Montastraea*: the role of phenotypic plasticity Proc 6th Int Coral Reef Symp 1988 3 393 398

[B91] Todd PA Ladle RJ Lewin-Koh NJI Chou LM Flesh or bone? Quantifying small-scale coral morphology using with-tissue and without-tissue techniques Mar Biol 2004 145 323 328 10.1007/s00227-004-1324-8

[B92] Todd PA Ladle RJ Lewin-Koh NJI Chou LM Genotype × environment interactions in transplanted clones of the massive corals *Favia speciosa *and *Diploastrea heliopora* Mar Ecol-Prog Ser 2004 271 167 182 10.3354/meps271167

[B93] Todd PA Sidle RC Lewin-Koh NJI An aquarium experiment for identifying the physical factors inducing morphological change in two massive scleractinian corals J Exp Mar Biol Ecol 2004 299 97 113 10.1016/j.jembe.2003.09.005

[B94] Todd PA Morphological plasticity in scleractinian corals Biol Rev 2008 83 315 337 10.1111/j.1469-185X.2008.00045.x 18979594

[B95] Budd AF Johnson KG Potts DC Recognizing morphospecies in colonial reef corals: I. Landmark-based methods Paleobiology 1994 20 484 505

[B96] Wallace CC Willis BL Systematics of the coral genus *Acropora*: implications of new biological findings for species concepts Annu Rev Ecol Syst 1994 25 237 262

[B97] Daly M Brugler MR Cartwright P Collins AG Dawson MN Fautin DG France SC McFadden CS Opresko DM Rodriguez E Romano SL Stake JL The phylum Cnidaria: A review of phylogenetic patterns and diversity 300 years after Linnaeus Zootaxa 2007 1668 127 182

[B98] Veron JEN Odorico DM Chen CA Miller DJ Reassessing evolutionary relationships of scleractinian corals Coral Reefs 1996 15 1 9

[B99] Knowlton N Budd AF Jackson JBC, Lidgard S, McKinney, FK Recognizing coral species past and present Evolutionary Patterns: Growth, Form, and Tempo in the Fossil Record 2001 Chicago: University of Chicago Press 97 119

[B100] Fukami H Nomura K Existence of a cryptic species of *Montastraea valenciennesi *(Milne Edwards and Haime, 1848) in Wakayama, southern Honshu, Japan (in Japanese) J Jpn Coral Reef Soc 2009 11 25 31 10.3755/jcrs.11.25

[B101] Chevalier JP Les scléractiniaires de la Mélanésie Française (Nouvelle-Calédonie, Iles Chesterfield, Iles Loyauté, Nouvelles Hébrides). Première partie Expéd Française récifs coralliens Nouvelle Calédonie 1971 5 1 307

[B102] Matthai G A revision of the Recent colonial Astræidæ possessing distinct corallites Trans Linn Soc Lond 1914 17 1 140 10.1111/j.1096-3642.1914.tb00590.x

[B103] Matthai G Report of the madreporarian corals in the collection of the Indian Museum, Calcutta Mem Indian Mus 1924 8 1 59

[B104] Crossland C Madreporaria, Hydrocorallinae, *Heliopora *and *Tubipora* Great Barrier Reef Exped (1928-1929) Sci Rep 1952 6 85 257

[B105] Wells JW Recent corals of the Marshall Islands Geol Surv Prof Pap 1954 260-I 385 486

[B106] Nemenzo F Systematic studies on Philippine shallow water scleractinians. II. Suborder Faviida Nat Appl Sci Bull 1959 16 73 135

[B107] Wijsman-Best M Systematics and ecology of New Caledonian Faviinae (Coelenterata - Scleractinia) Contrib Zool 1972 42 3 90

[B108] Wijsman-Best M Biological results of the Snellius Expedition. XXV Faviidae collected by the Snellius Expedition. I. The genus *Favia* Zool Meded Leiden 1974 48 249 261

[B109] Scheer G Pillai CSG Report on the stony corals from the Red Sea Zoologica 1983 131 1 198

[B110] Nemenzo F Studies on the systematics of scleractinian corals in the Philippines Proc 4th Int Coral Reef Symp 1981 1 25 32

[B111] Lamarck JBP Histoire Naturelle des Animaux sans Vertèbres (Tome Second) 1816 Paris: Verdière

