# Supplementary material for: Cleaning up the 'Bigmessidae': Molecular phylogeny of scleractinian corals from Faviidae, Merulinidae, Pectiniidae and Trachyphylliidae
Source: BMC Evol Biol. 2011 Feb 7;11:37. doi: 10.1186/1471-2148-11-37 (PMC3042006; doi:10.1186/1471-2148-11-37)
Supplement: Additional file 1 — 'Bigmessidae' corals. Photographs of most coral specimens sequenced in this study. More photographs are available from the authors. [file 1471-2148-11-37-S1.PDF]

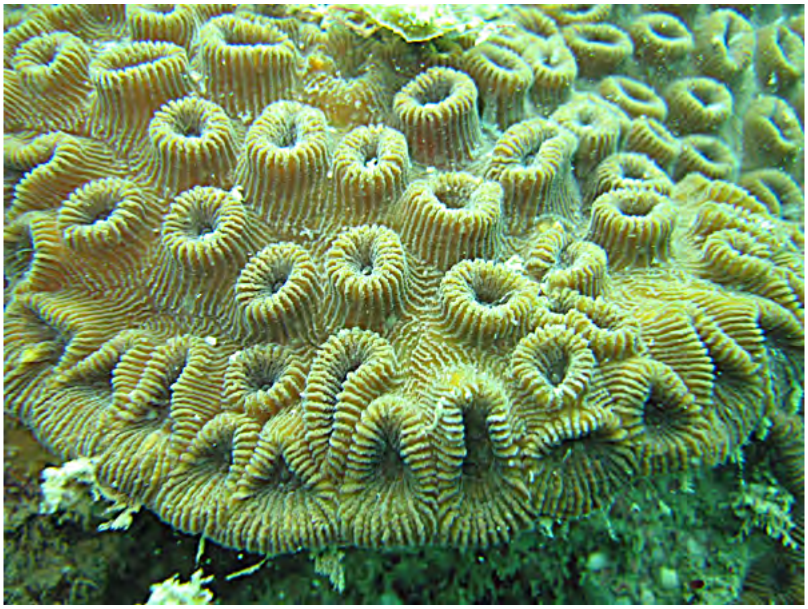

Barabattoia amicorum S047

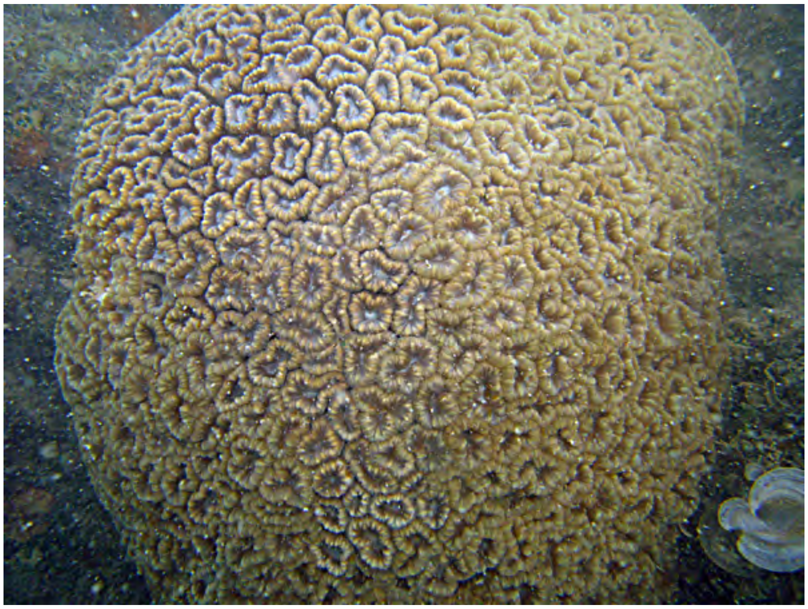

Caulastraea echinulata S041

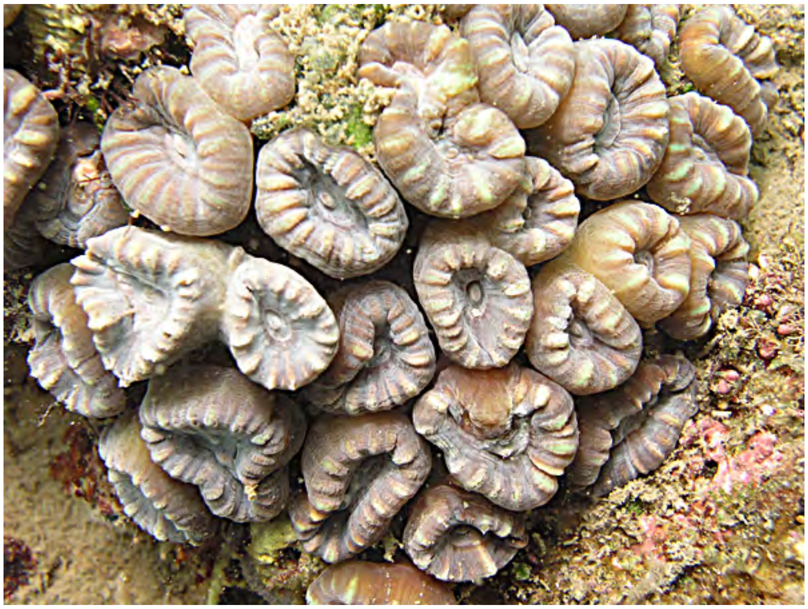

Caulastraea furcata P108

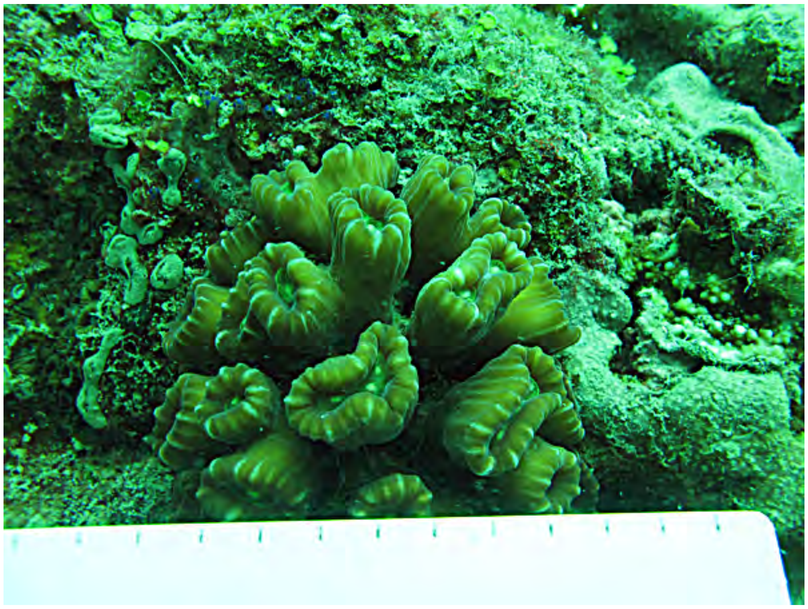

Caulastraea tumida G61875

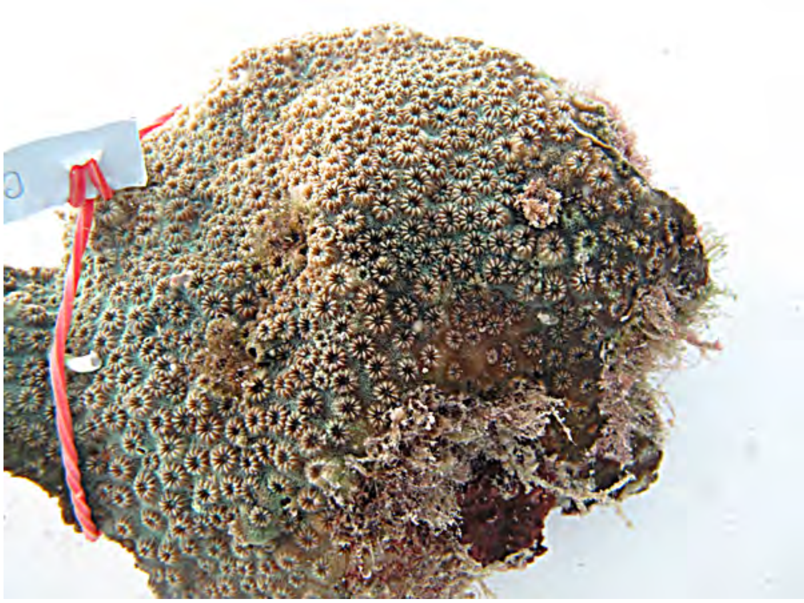

Cyphastrea chalcidicum G61902

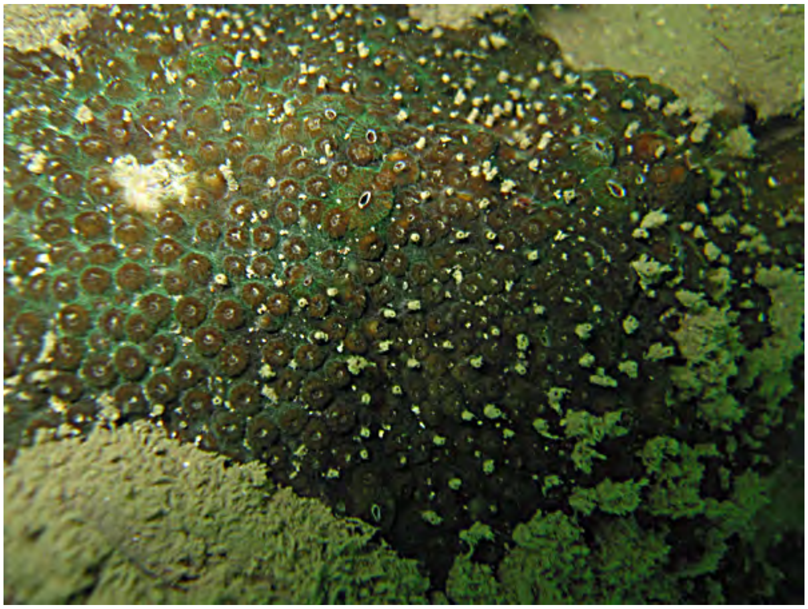

Cyphastrea serailia S120

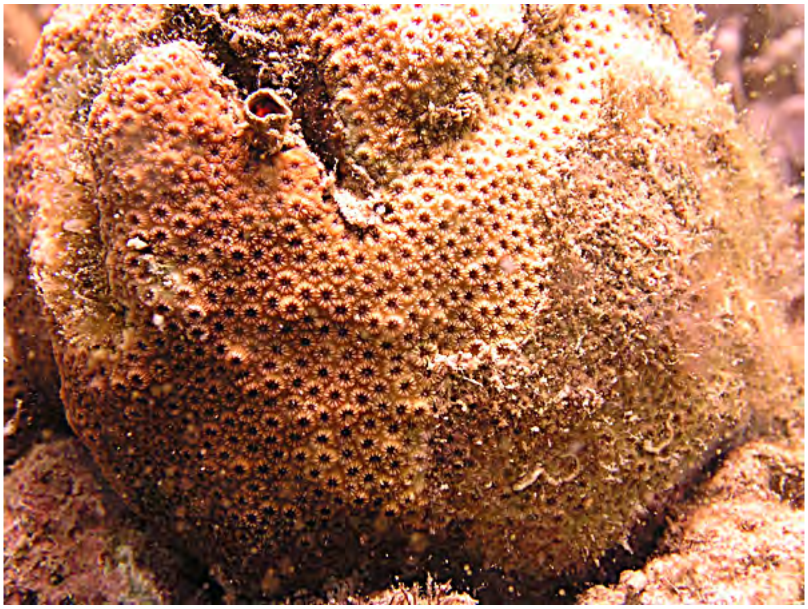

Cyphastrea serailia P120

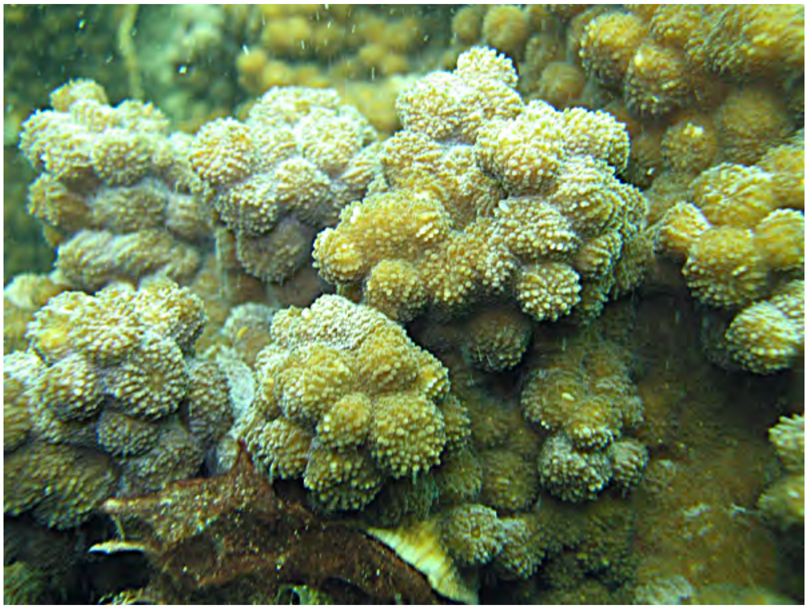

Echinopora gemmacea S120

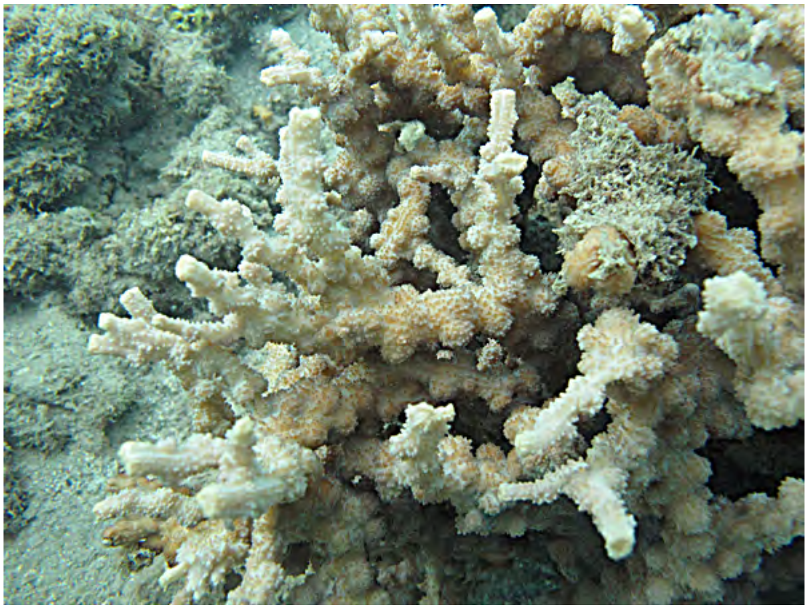

Echinopora horrida G61907

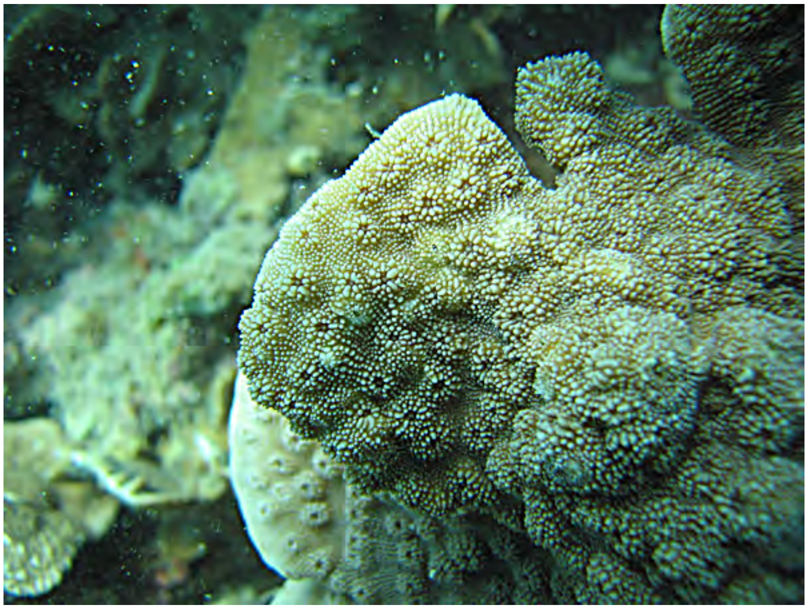

Echinopora lamellosa S109

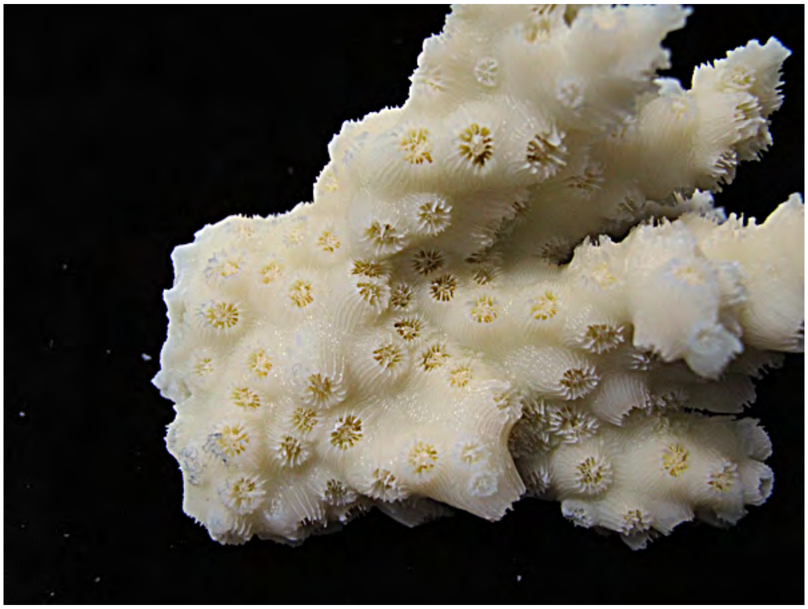

Echinopora mammiformis G61884

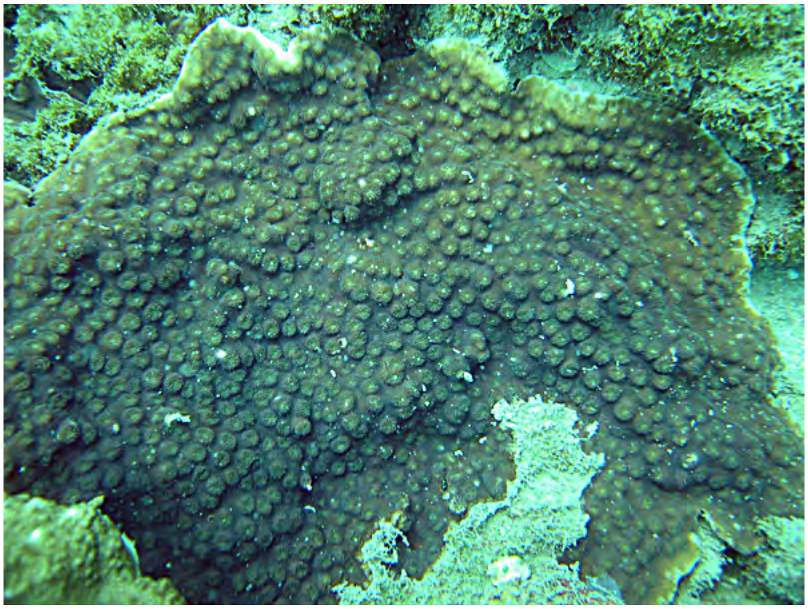

Echinopora pacificus S110

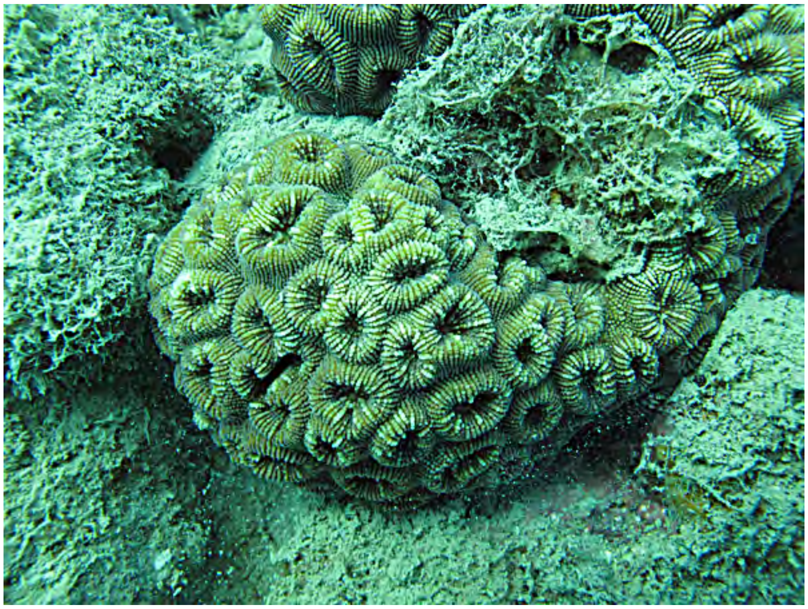

Favia danae G61885

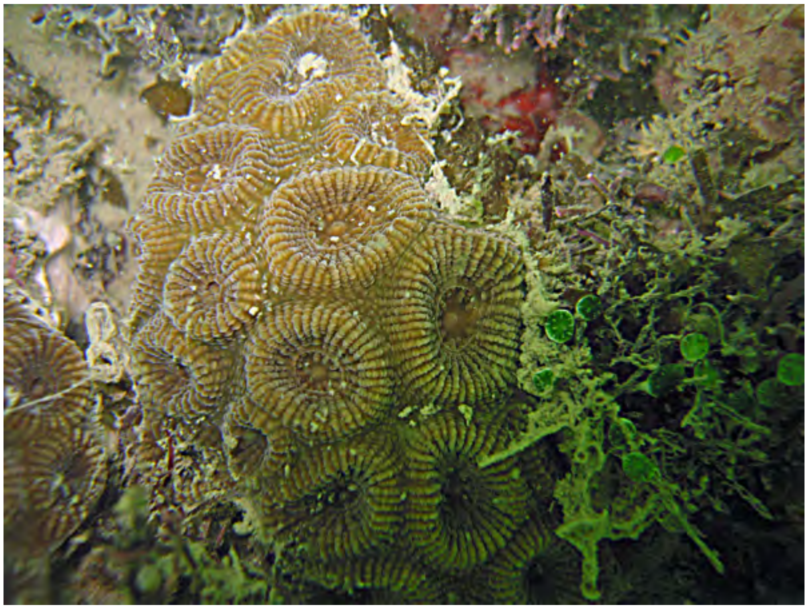

Favia danae S092

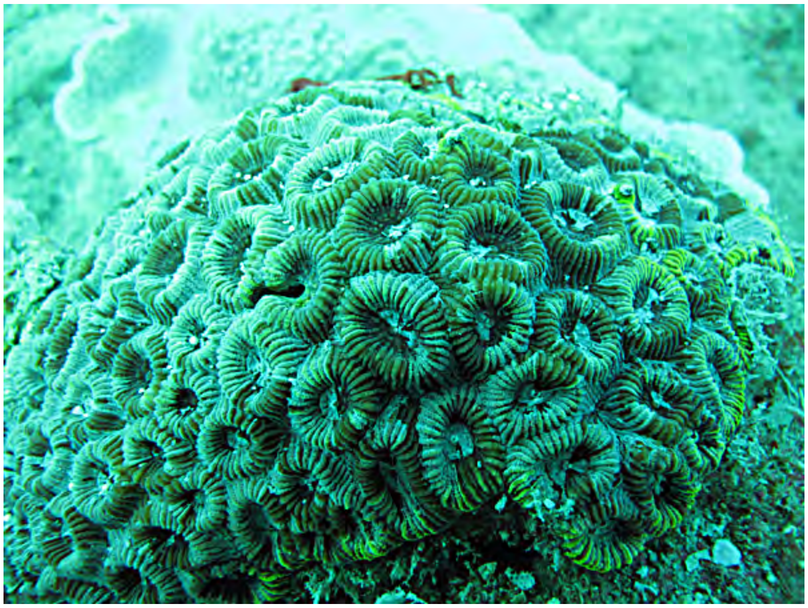

Favia favus G61880

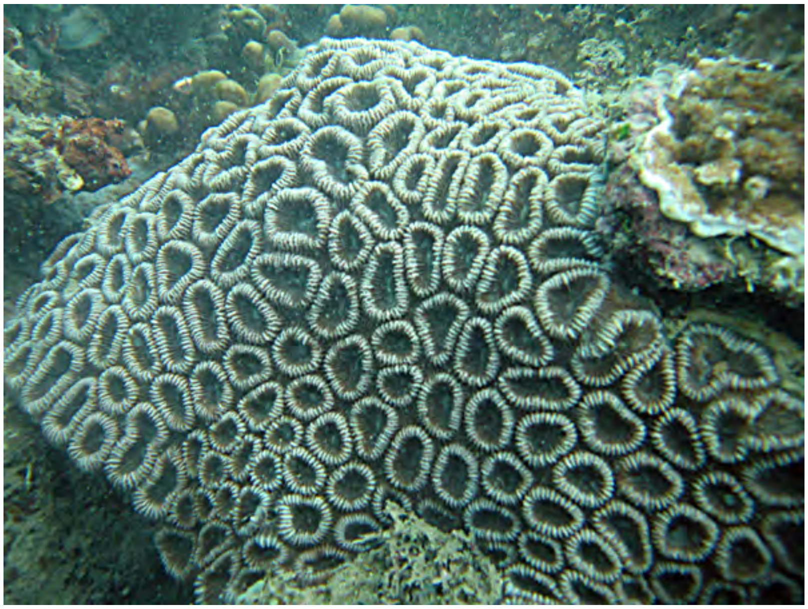

Favia favus S003

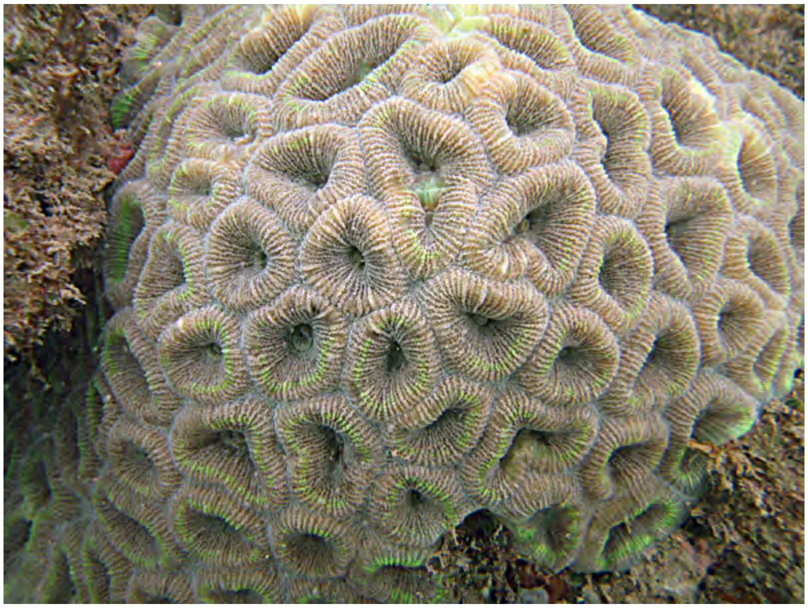

Favia favus S040

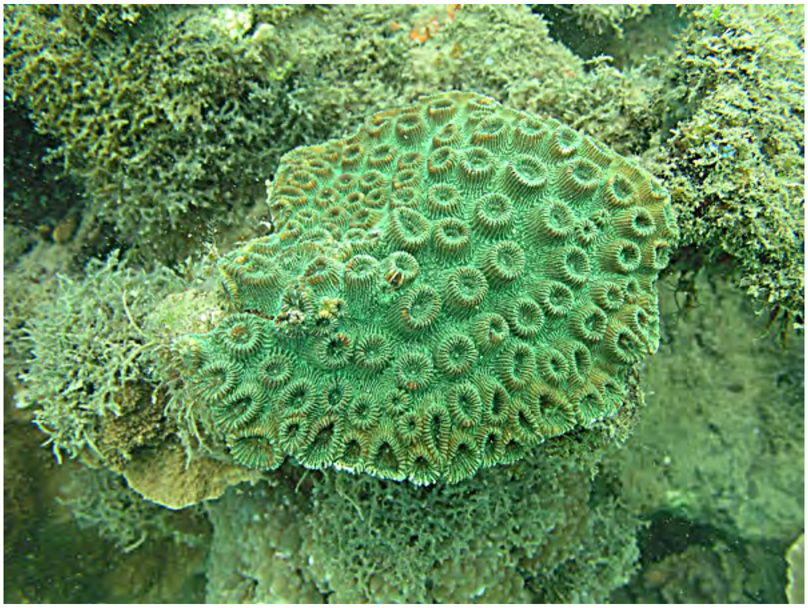

Favia cf. laxa S014

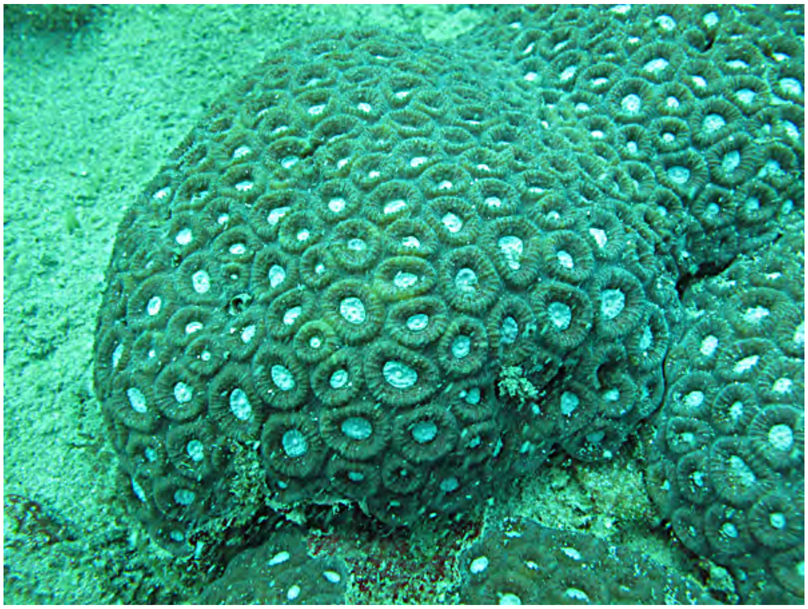

Favia lizardensis G61872

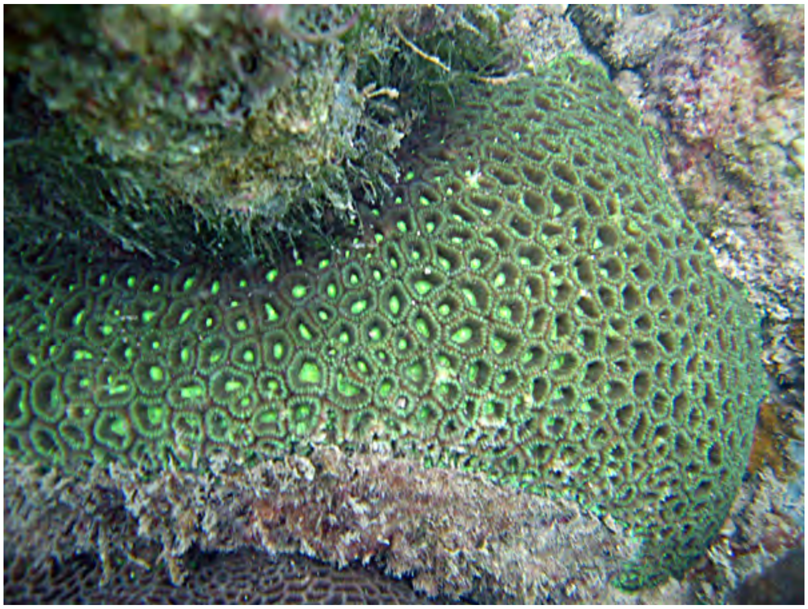

Favia lizardensis S072

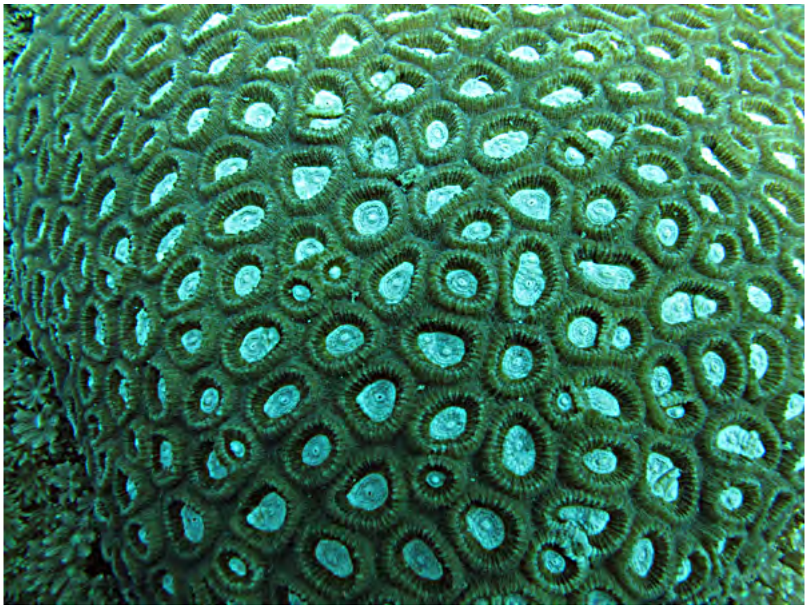

Favia lizardensis P136

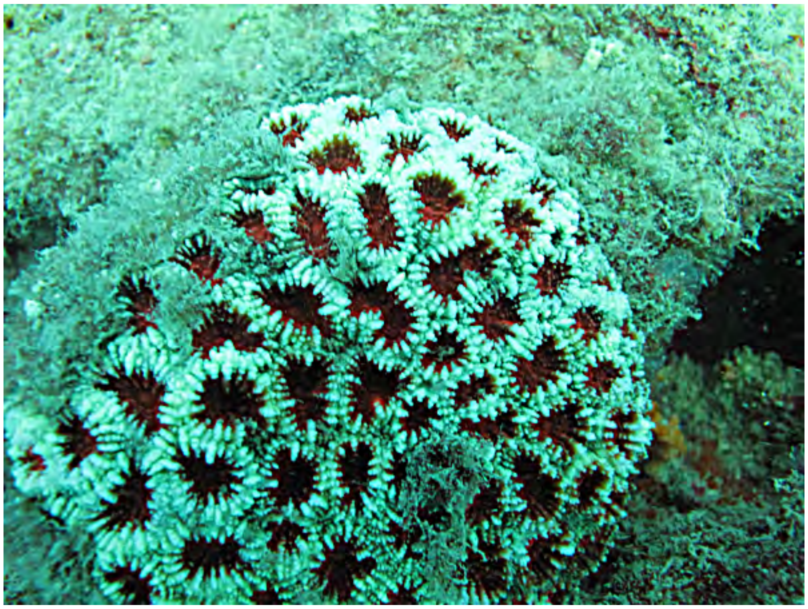

Favia matthaii G61881

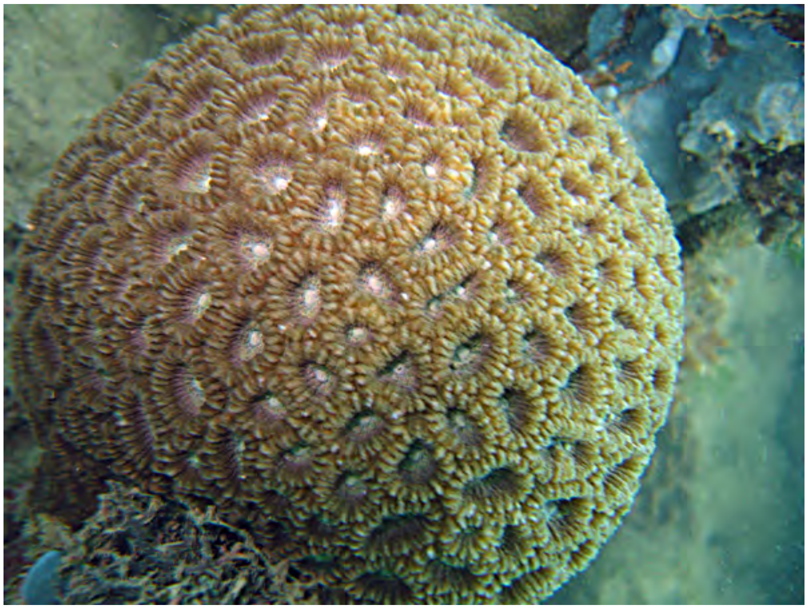

Favia matthaii S005

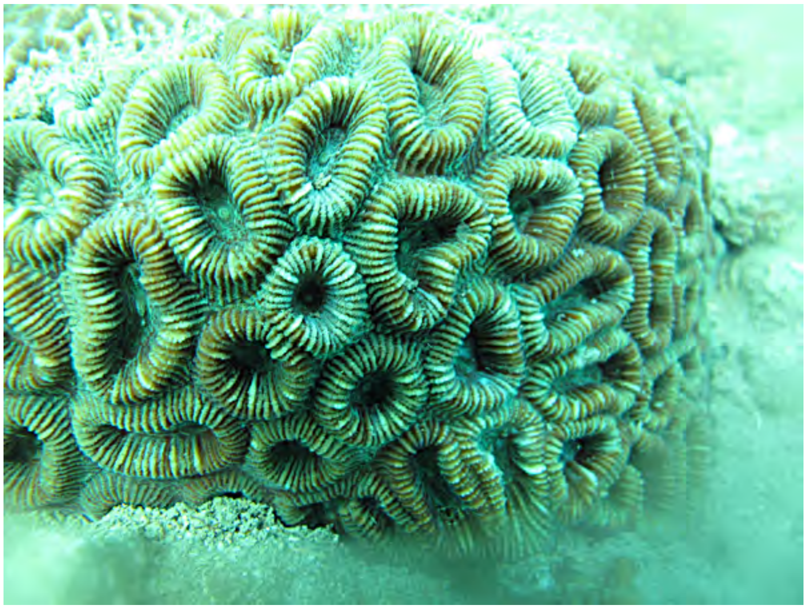

Favia cf. maritima G61912

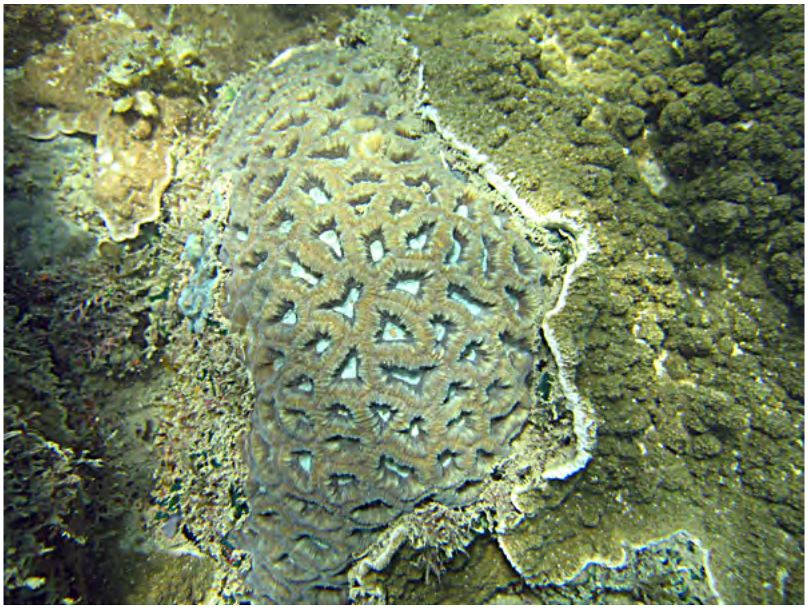

Favia maxima S052

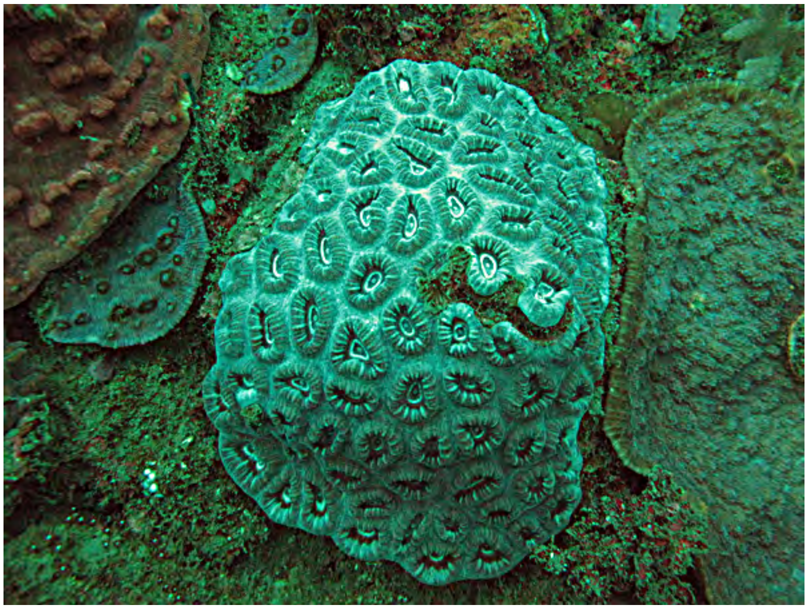

Favia maxima P142

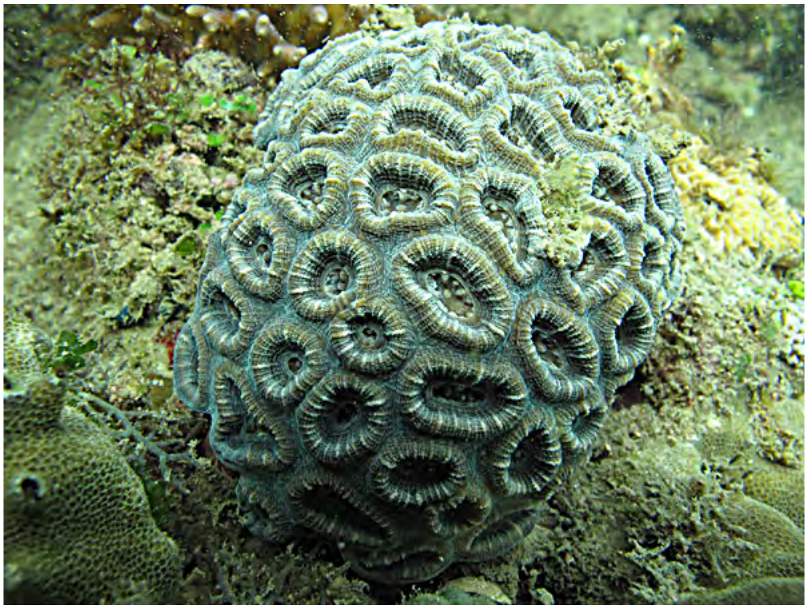

Favia cf. *maxima* P134

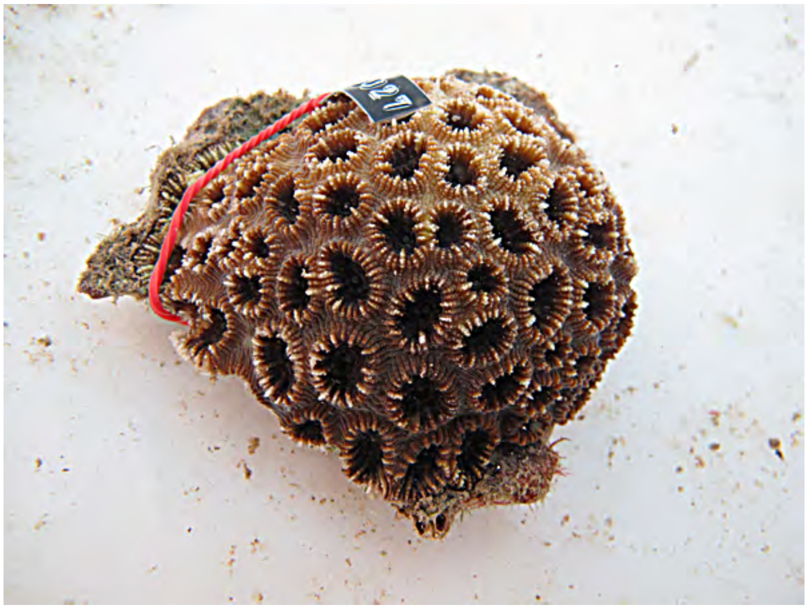

Favia pallida G61898

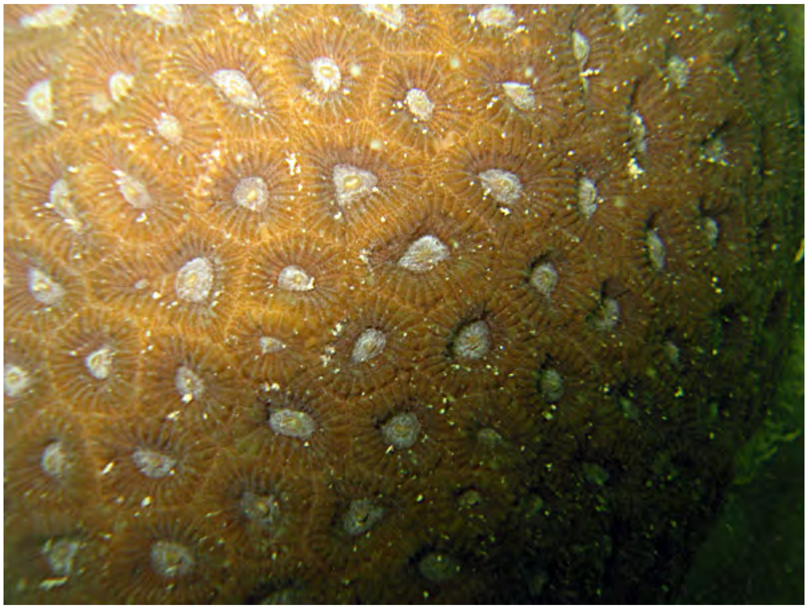

Favia pallida S036

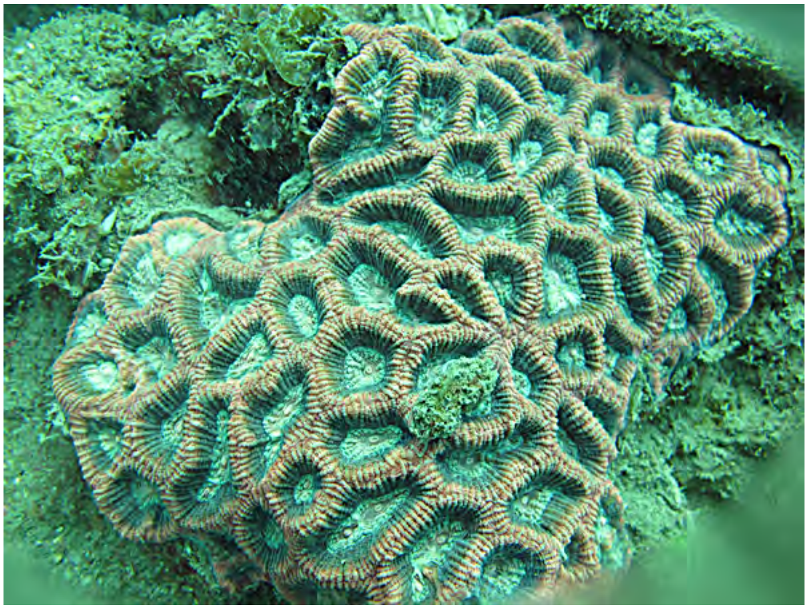

Favia rosaria G61911

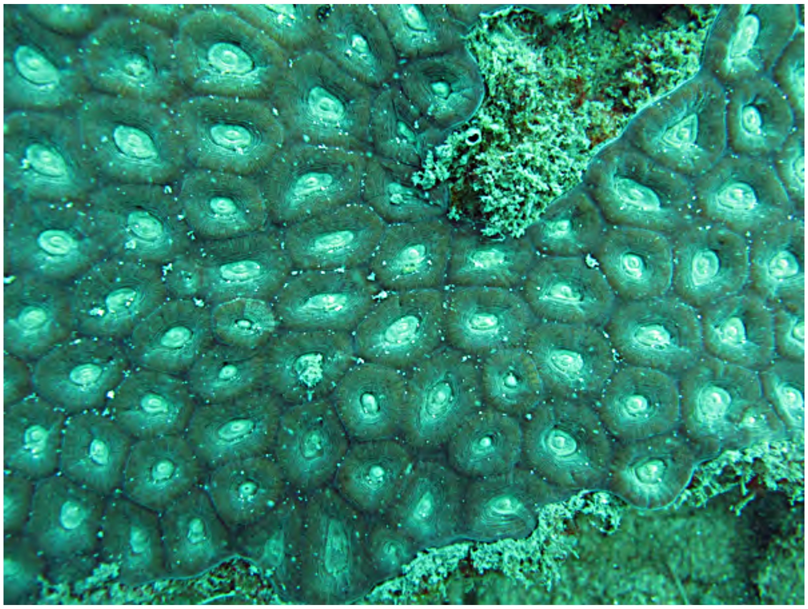

Favia rotundata G61874

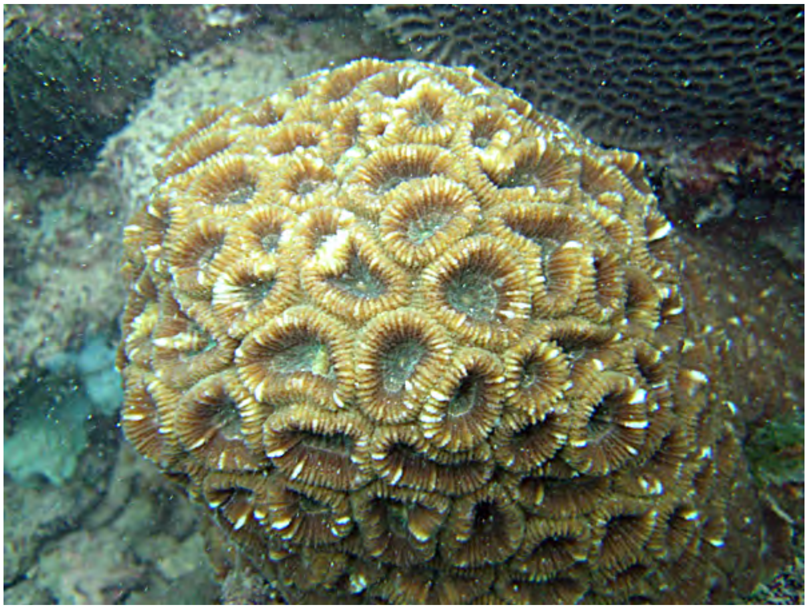

Favia speciosa S001

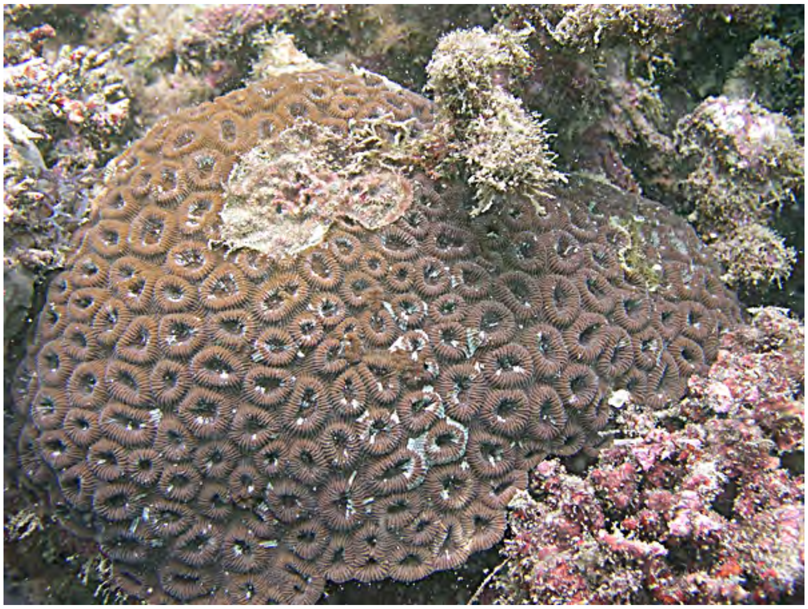

Favia speciosa P103

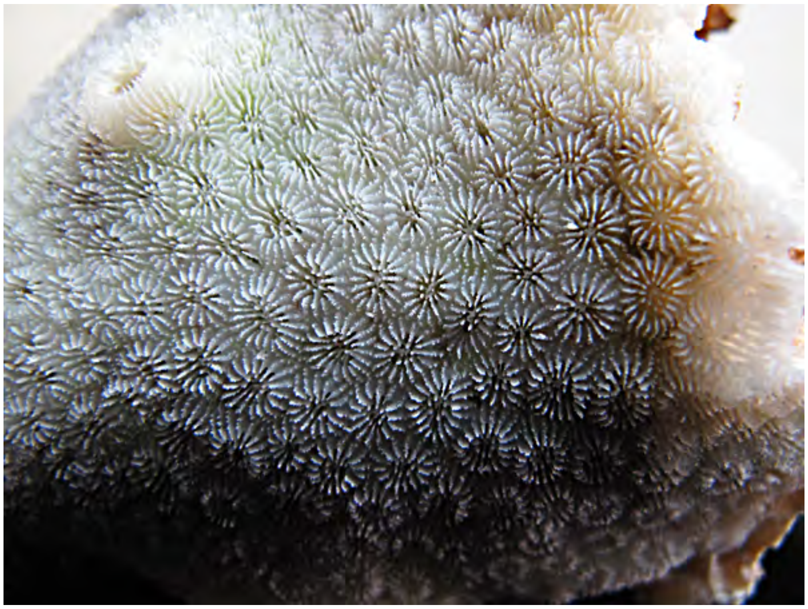

Favia stelligera P141

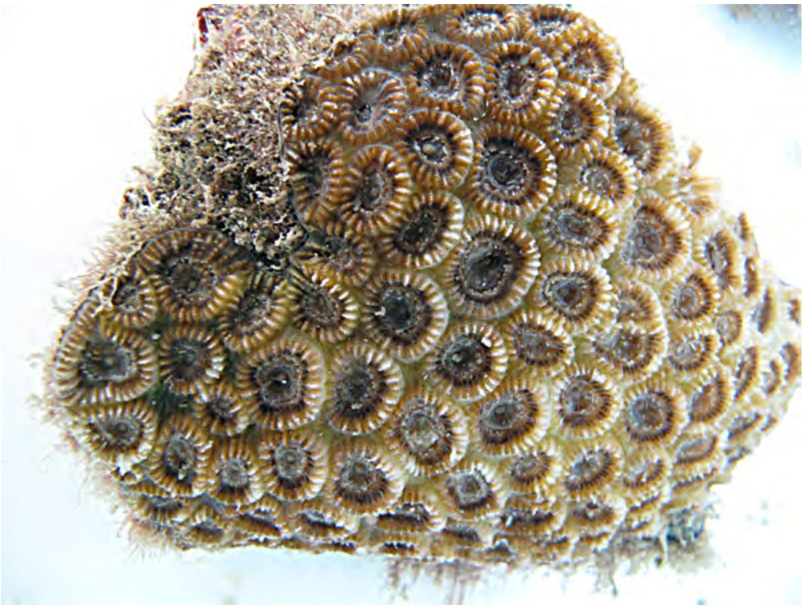

Favia truncatus G61897

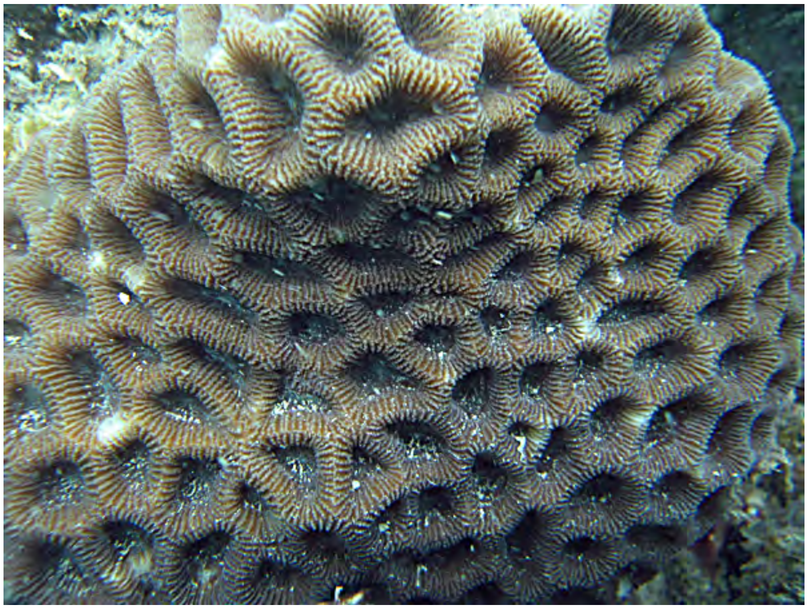

Favites abdita S002

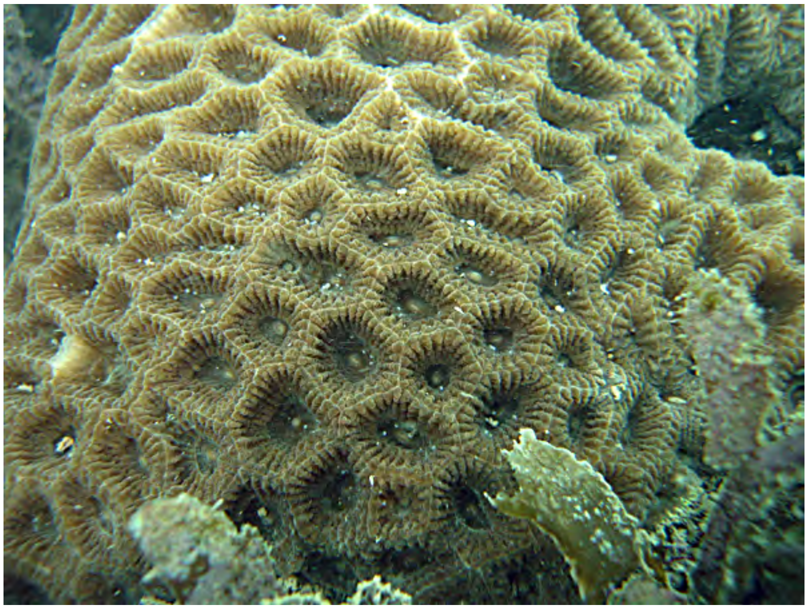

Favites chinensis S084

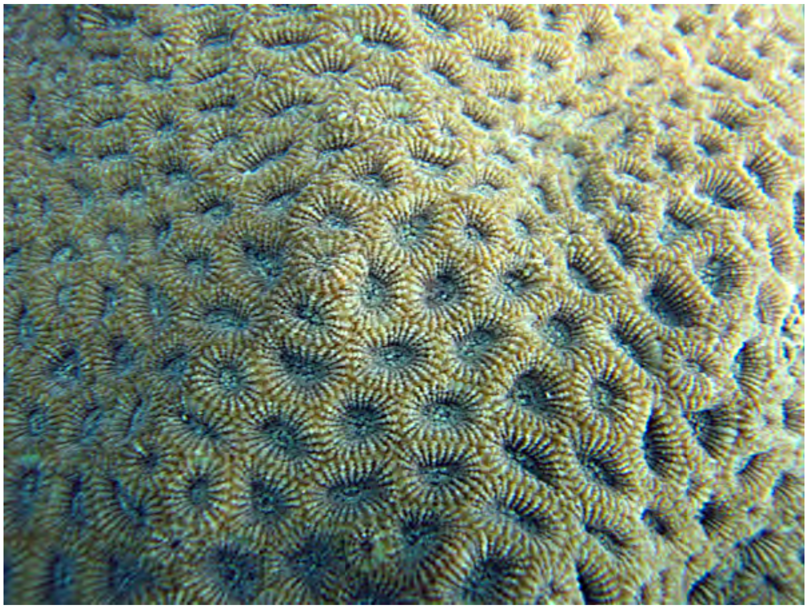

Favites complanata S007

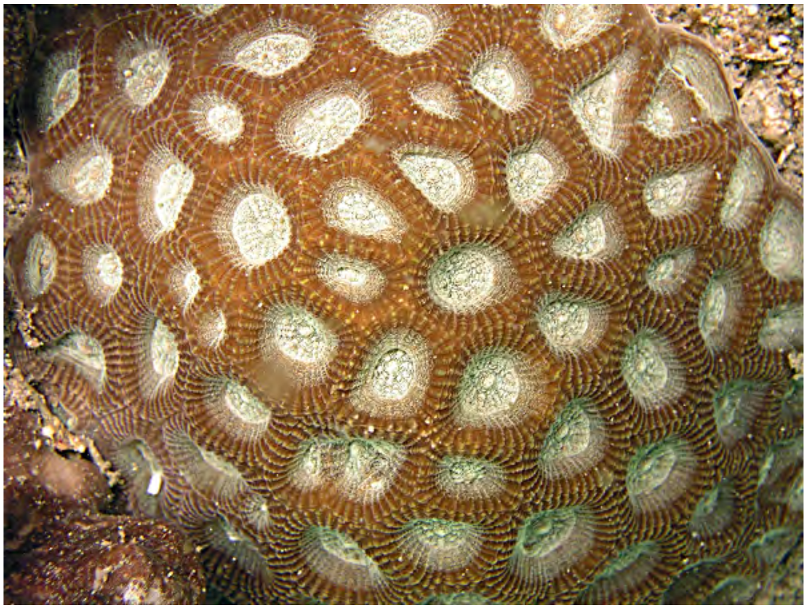

Favites flexuosa P116

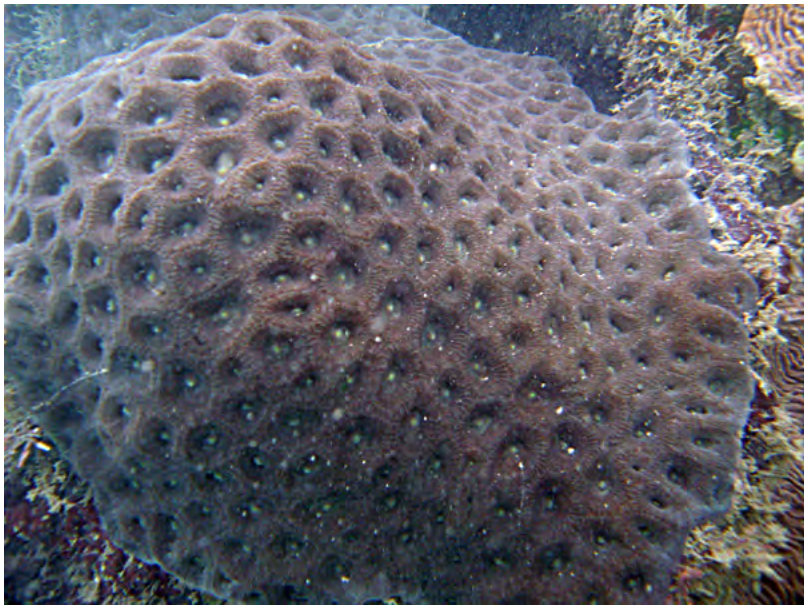

Favites paraflexuosa S100

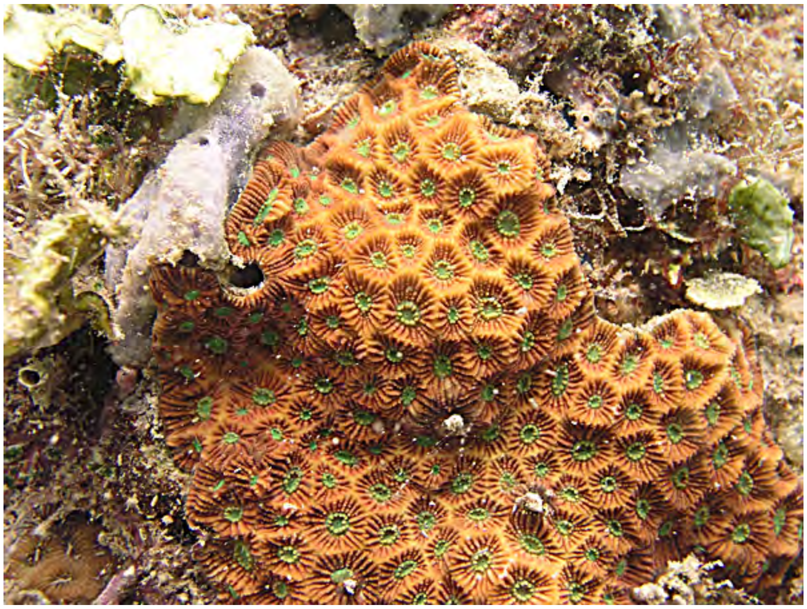

Favites pentagona P111

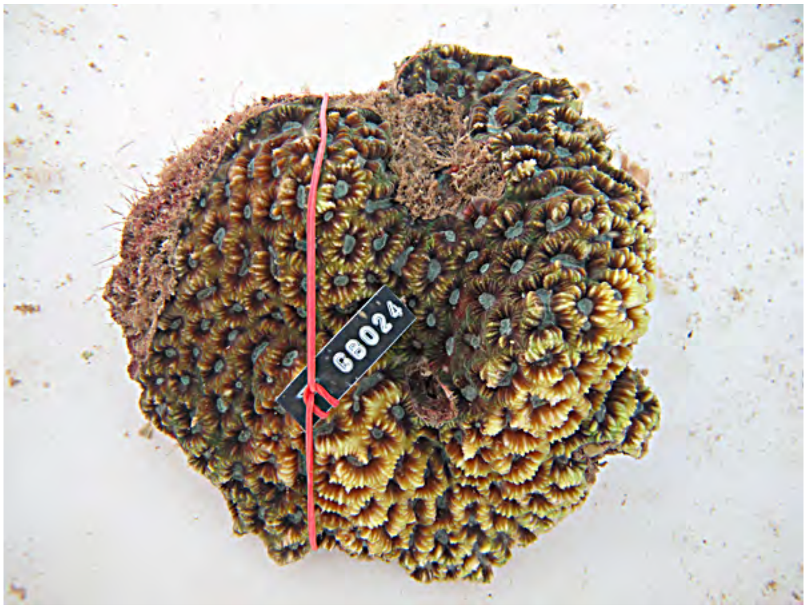

Favites russelli G61895

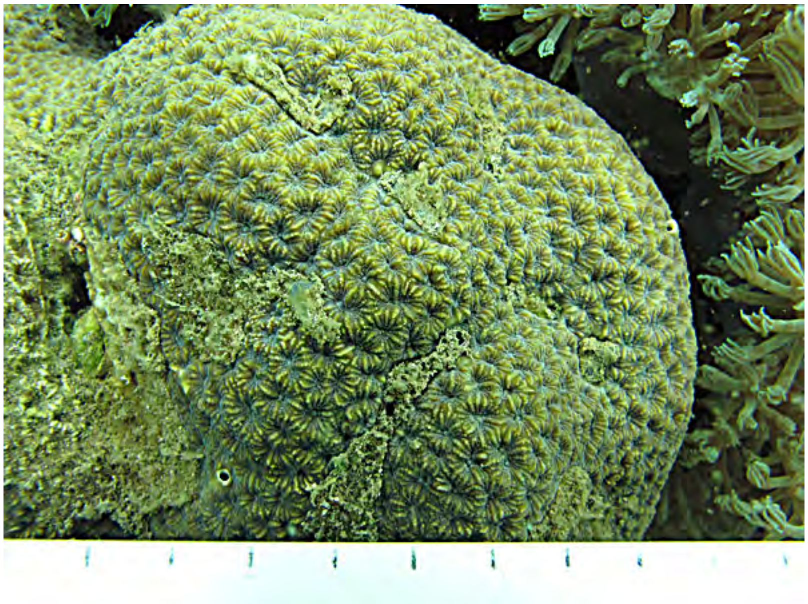

Favites stylifera P128

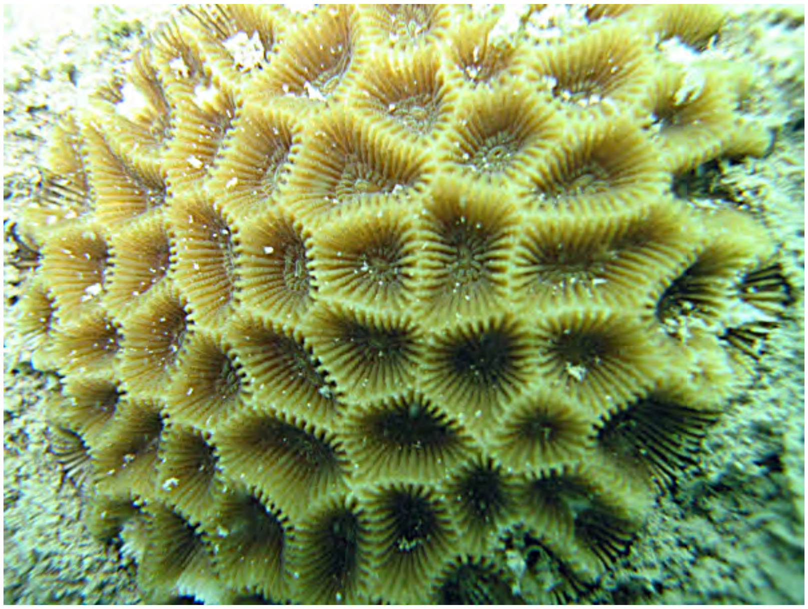

Goniastrea aspera S107

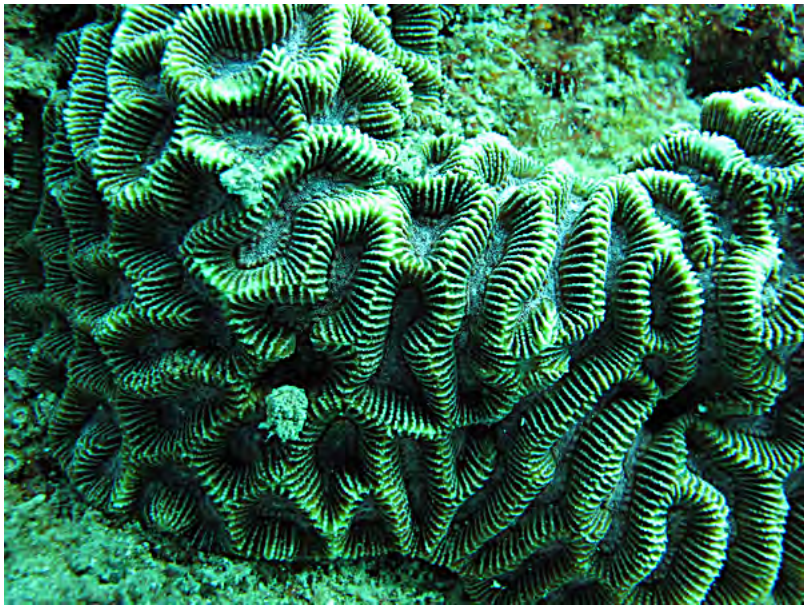

Goniastrea australensis G61876

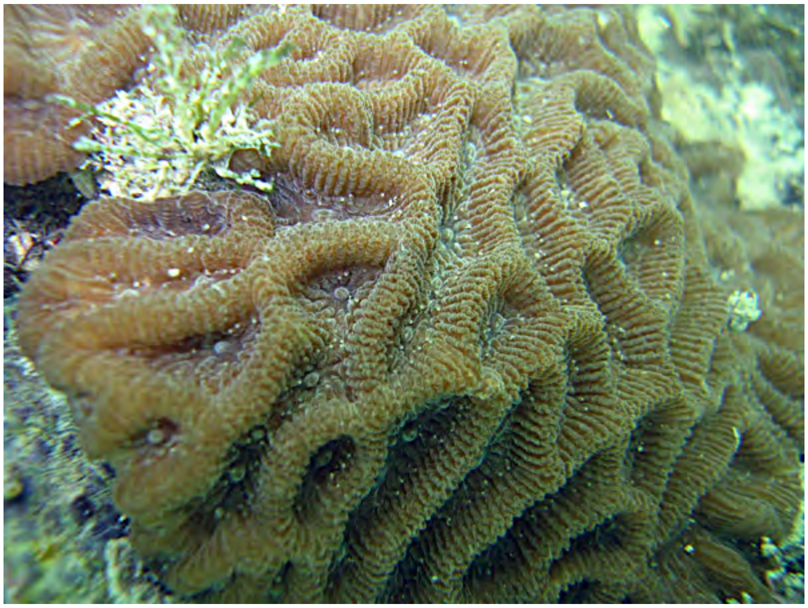

Goniastrea australensis S098

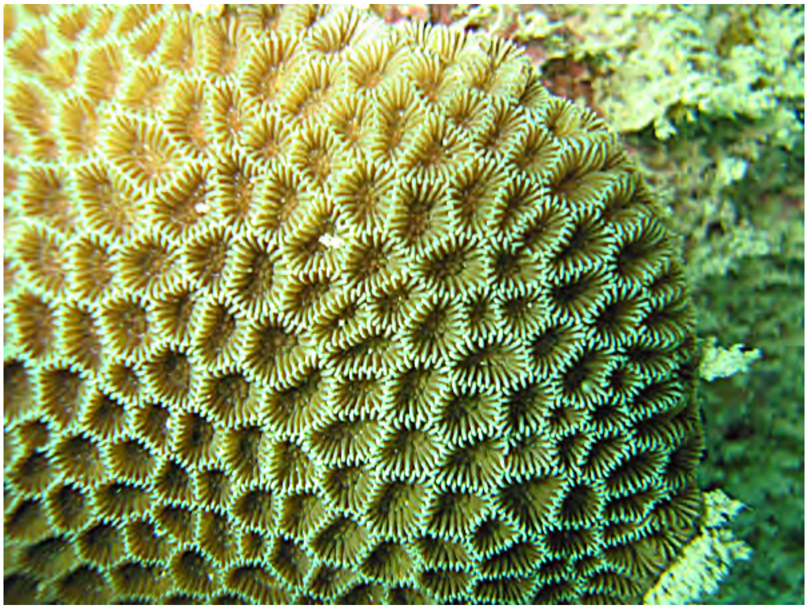

Goniastrea edwardsi S045

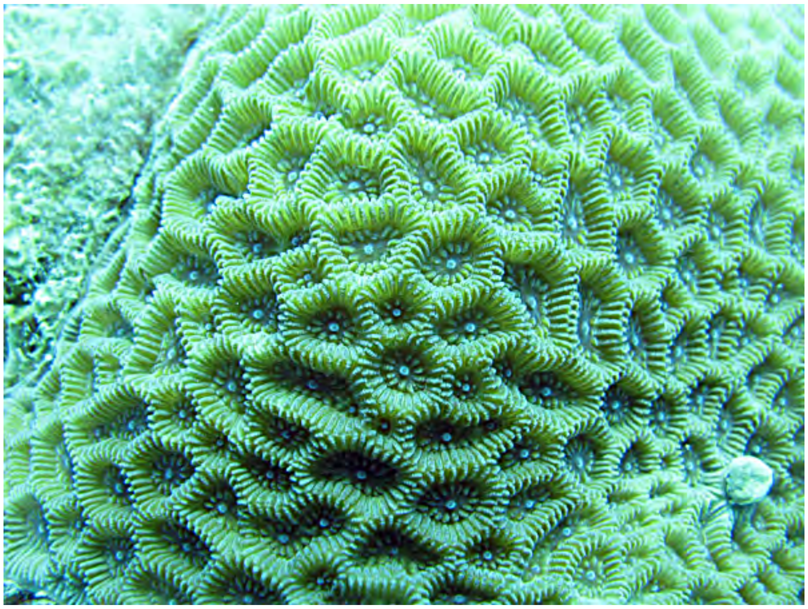

Goniastrea favulus G61877

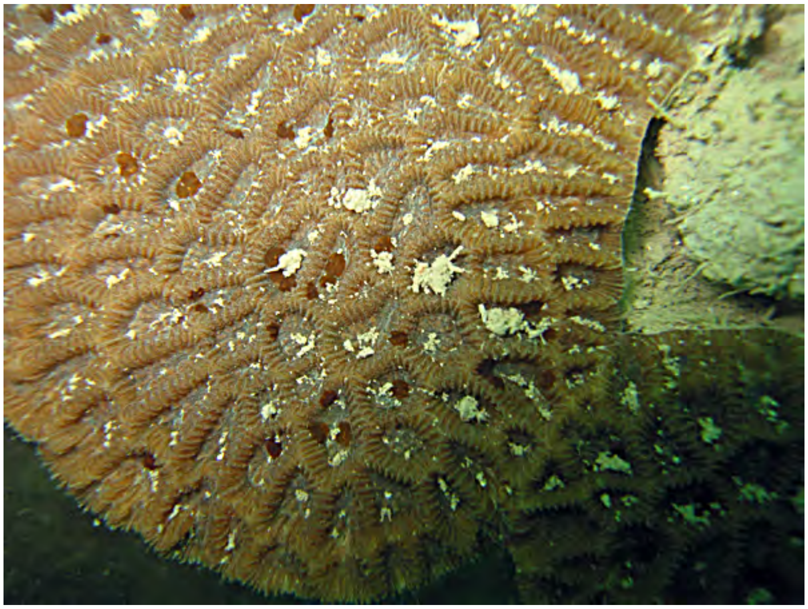

Goniastrea favulus S022

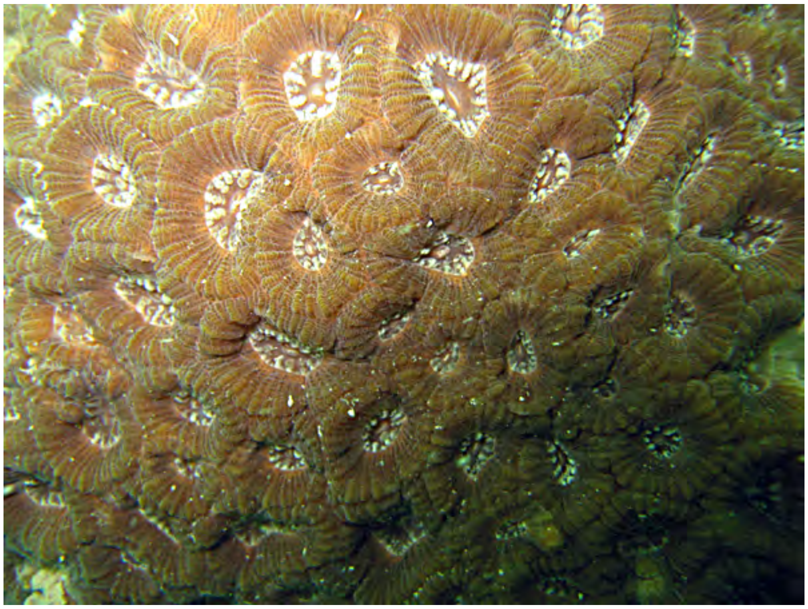

Goniastrea palauensis S021

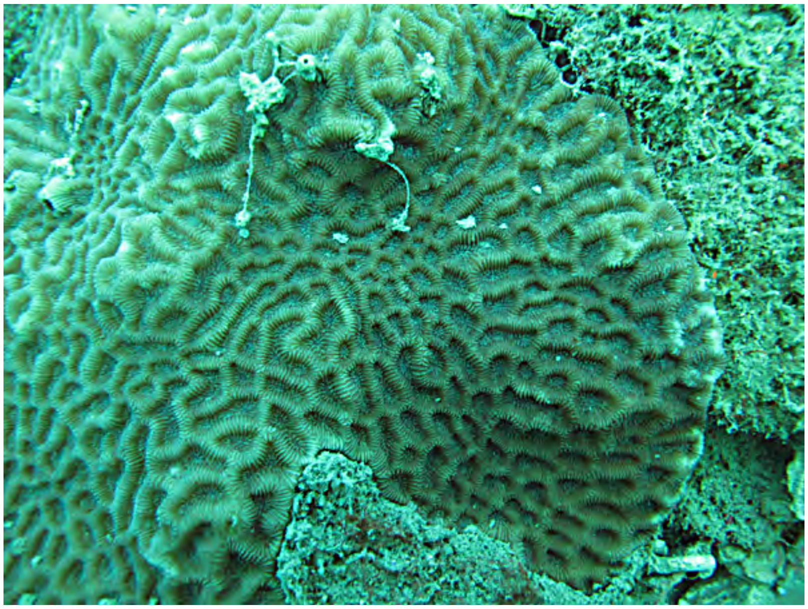

Goniastrea pectinata G61879

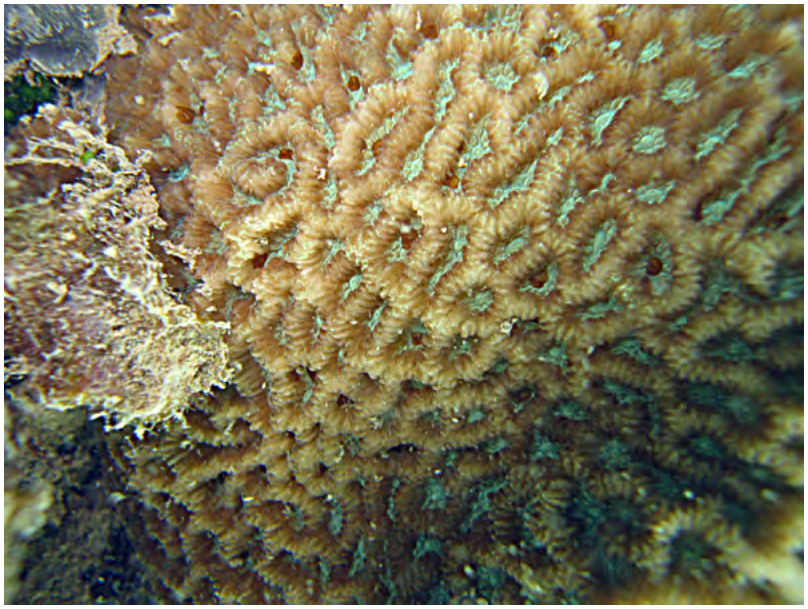

Goniastrea pectinata S043

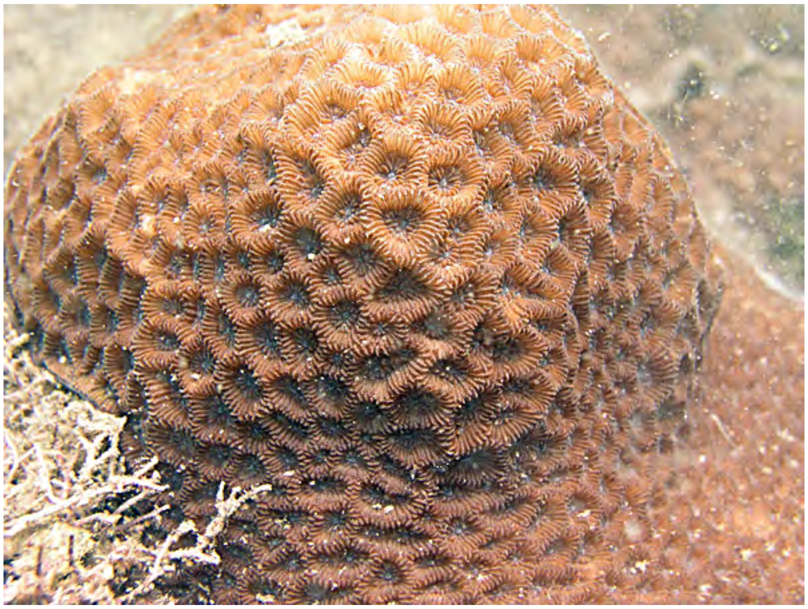

Goniastrea pectinata P110

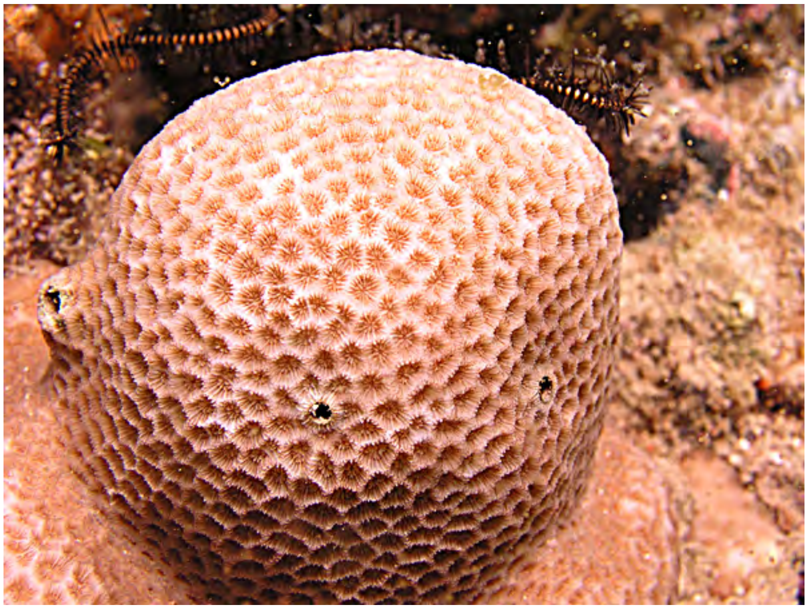

Goniastrea retiformis P119

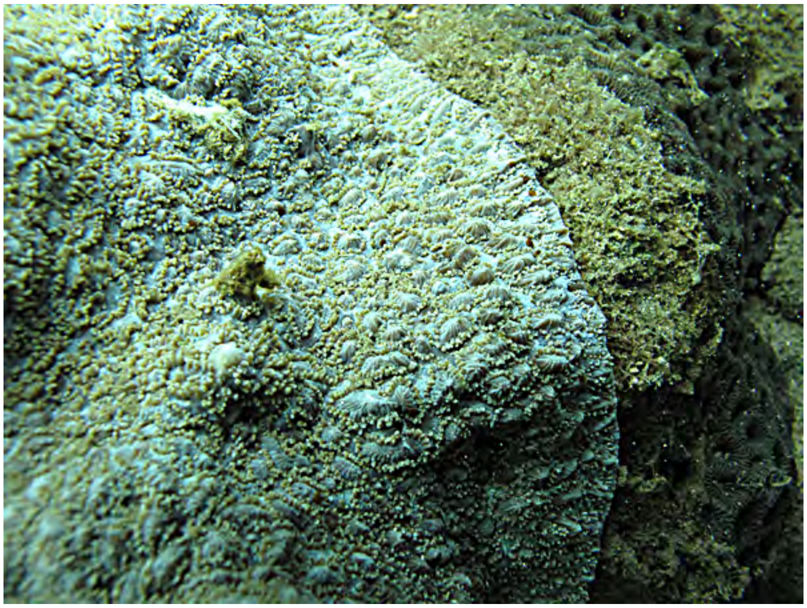

Hydnophora exesa P127

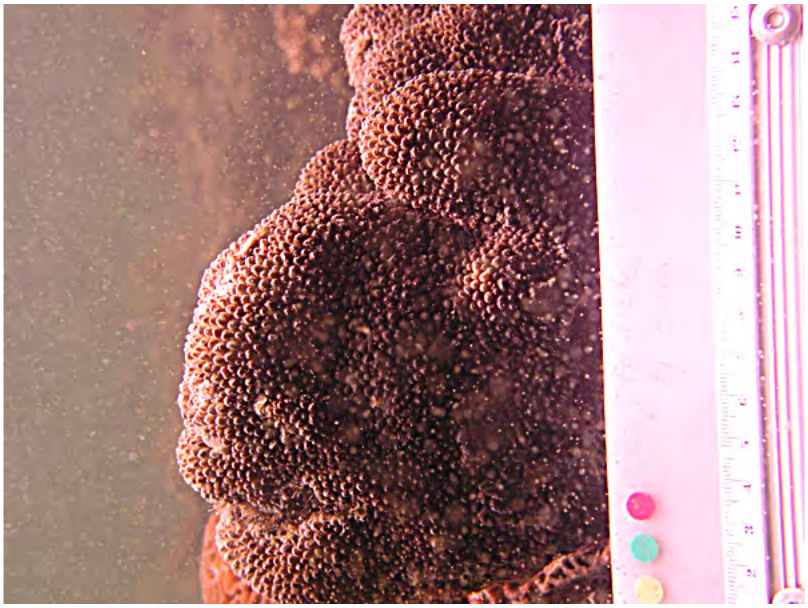

Hydnophora microconos P121

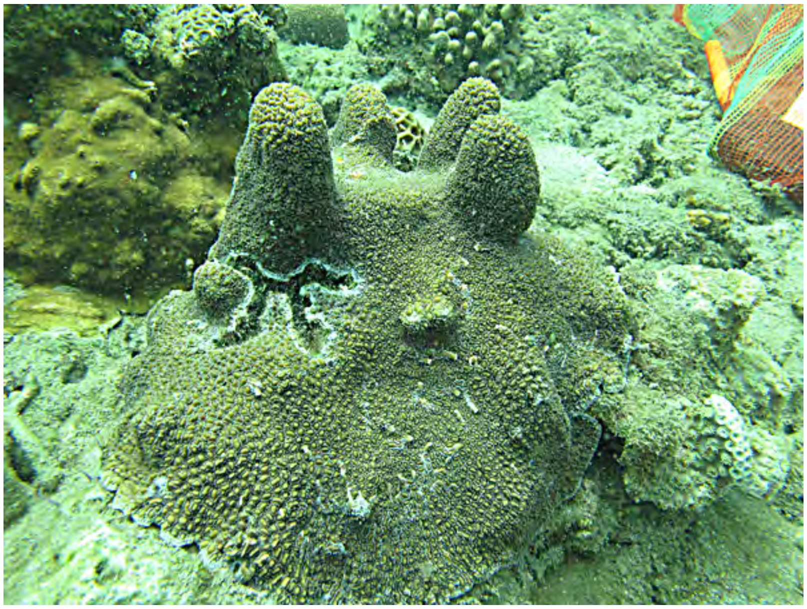

Hydnophora pilosa P138

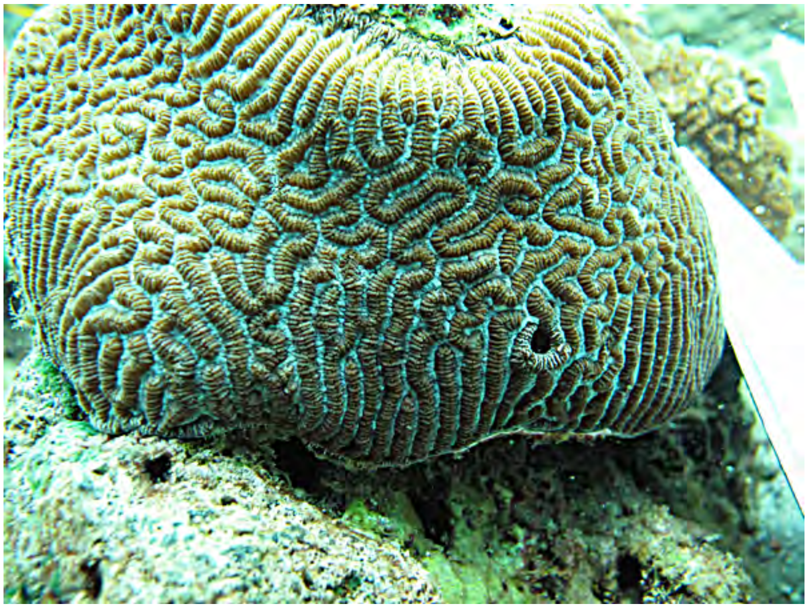

Leptoria irregularis P133

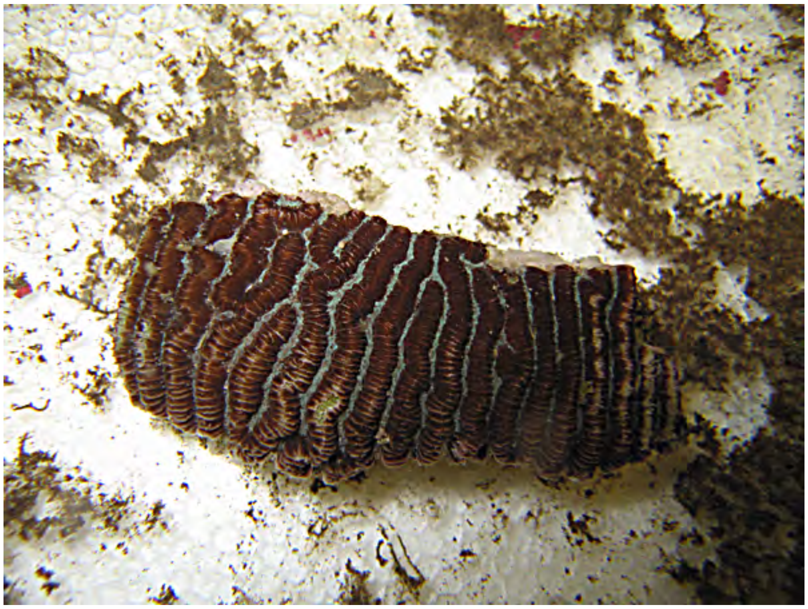

Leptoria phrygia S081

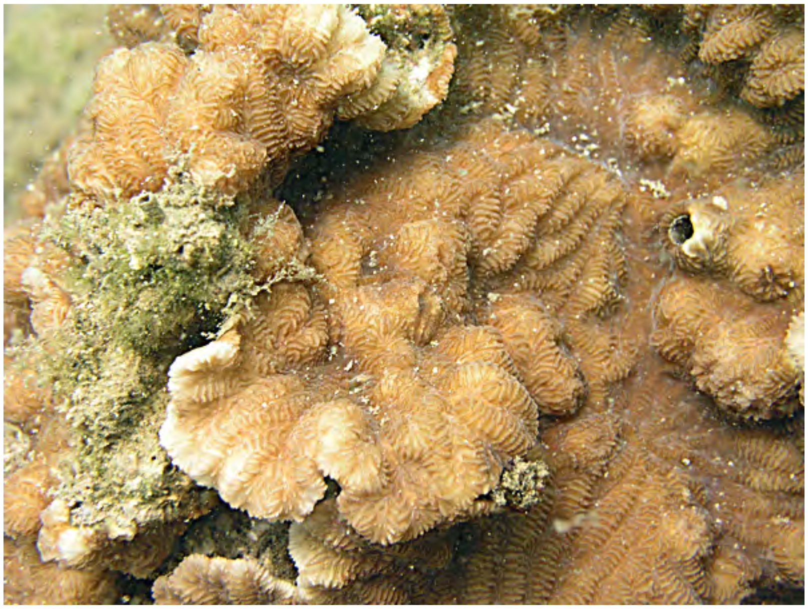

Merulina ampliata P106

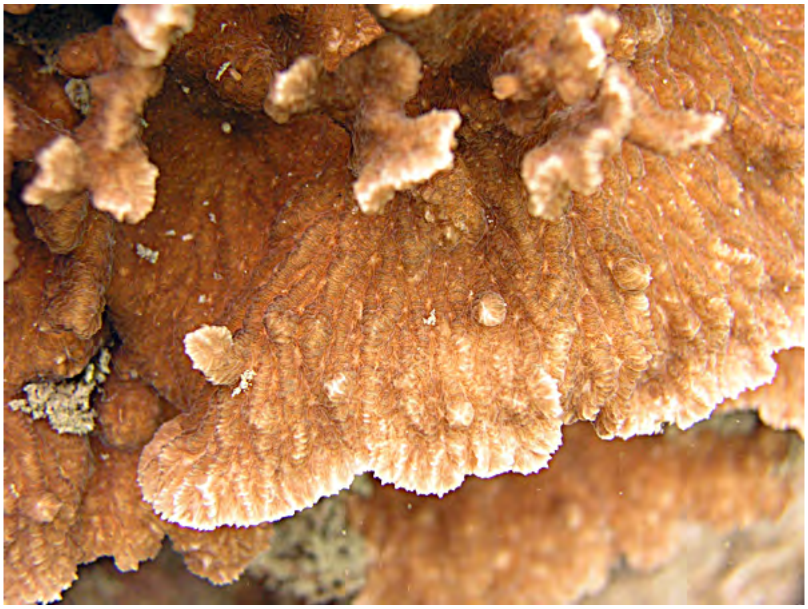
Merulina scabricula P114

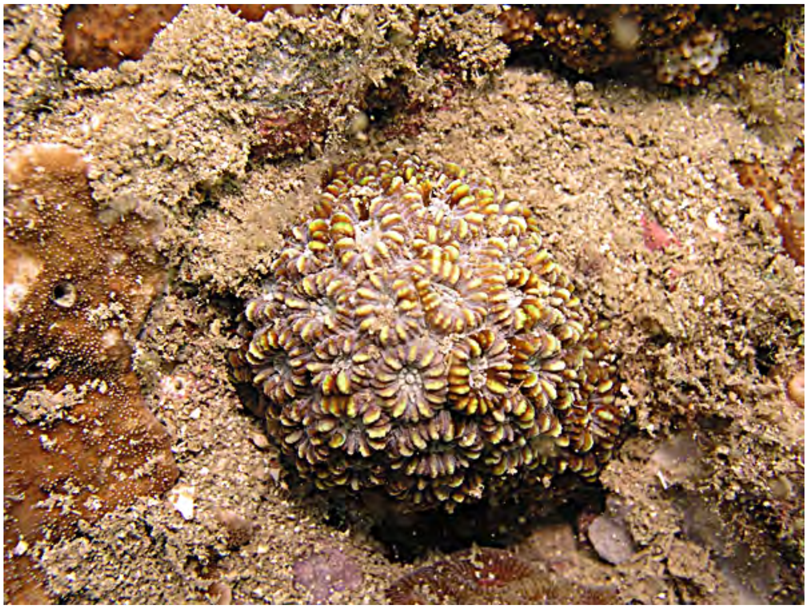
Montastraea cf. *annuligera* P117

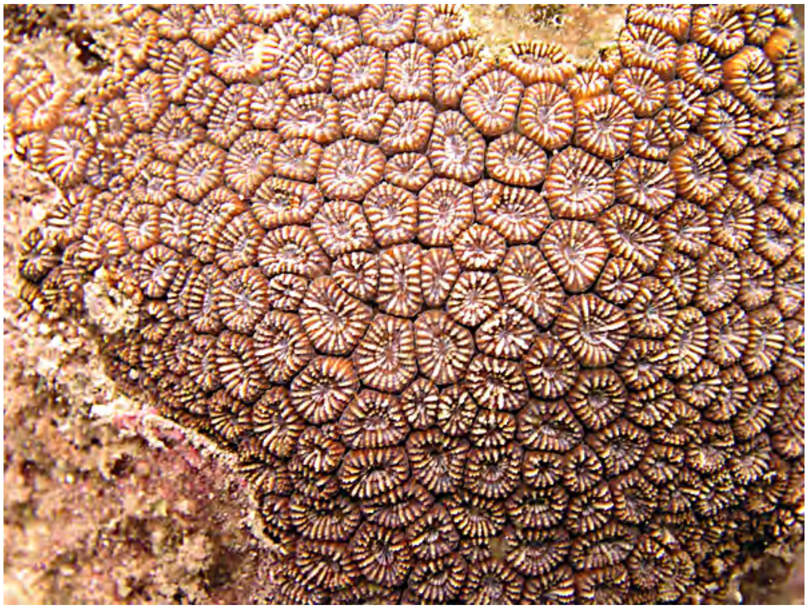
Montastraea colemani P118

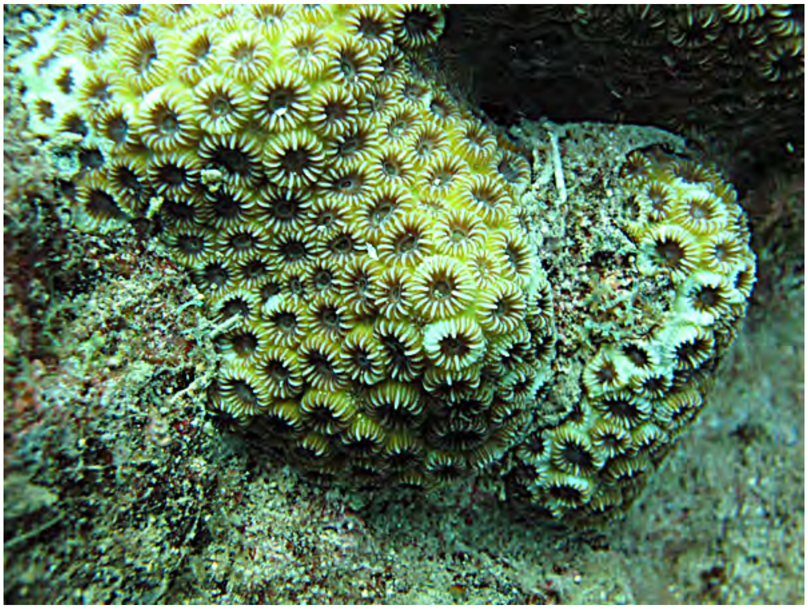
Montastraea curta G61882

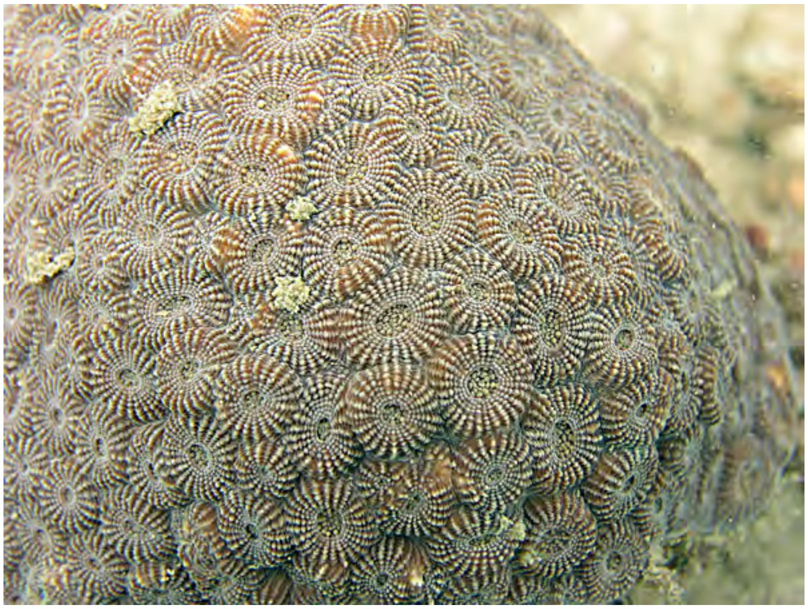
Montastraea magnistellata P109

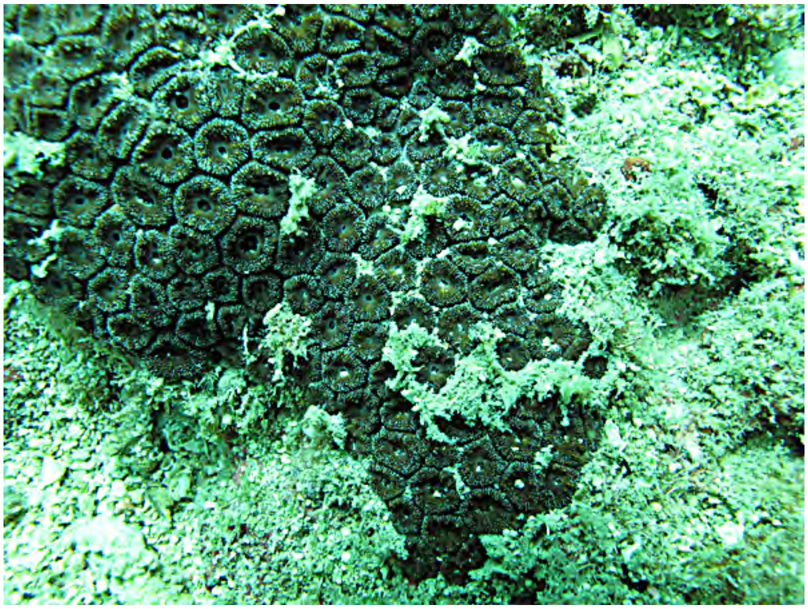
Montastraea multipunctata P131

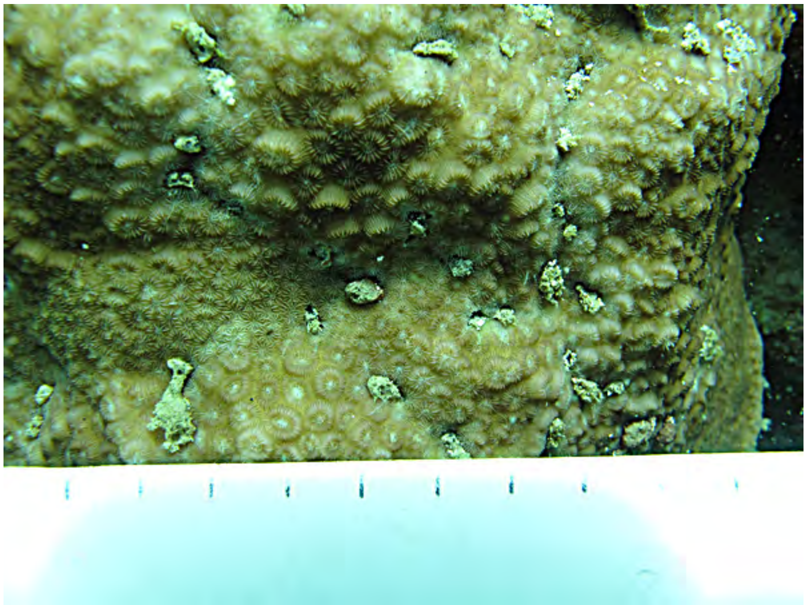
Montastraea salebrosa P139

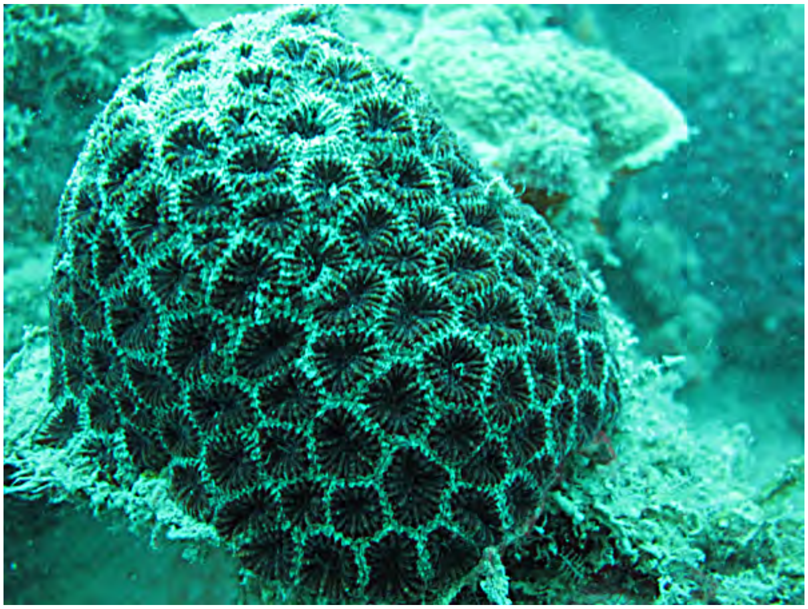
Montastraea valenciennesi G61904

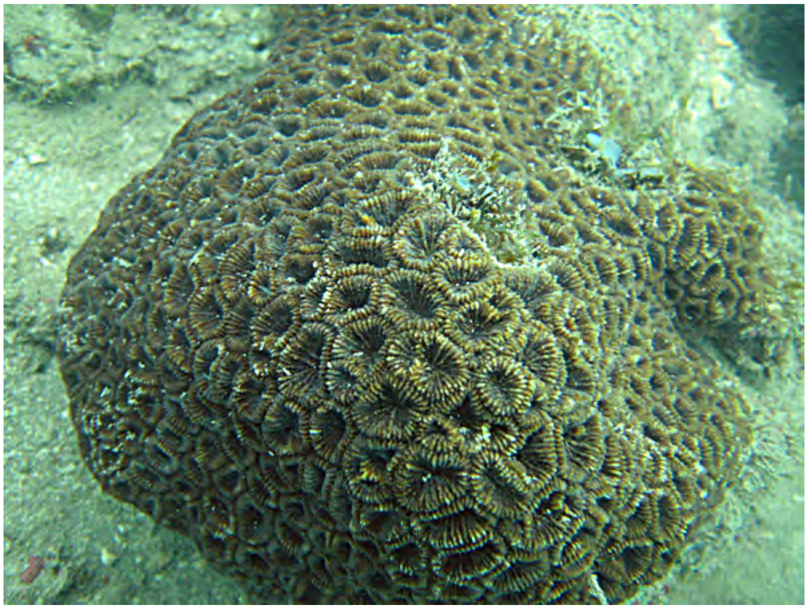
Montastraea valenciennesi S006

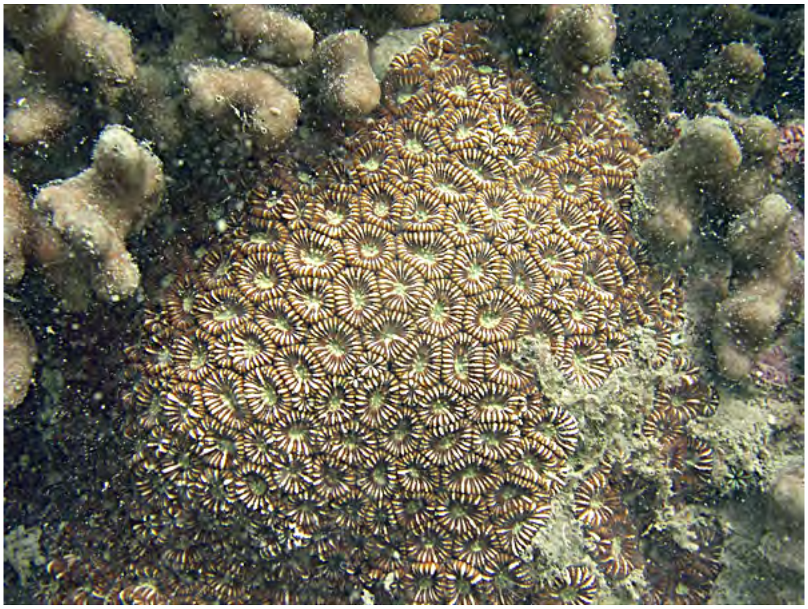
Montastraea valenciennesi P102.jpg

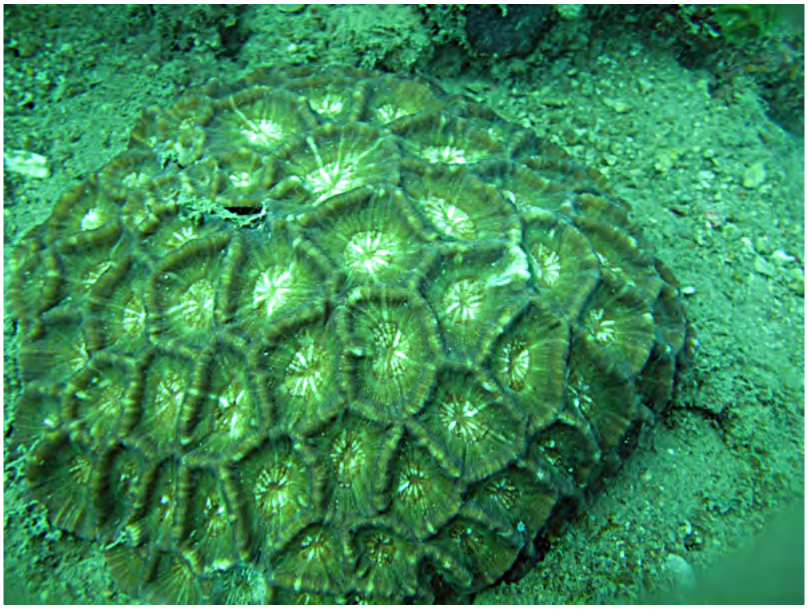
Moseleya latistellata G61909

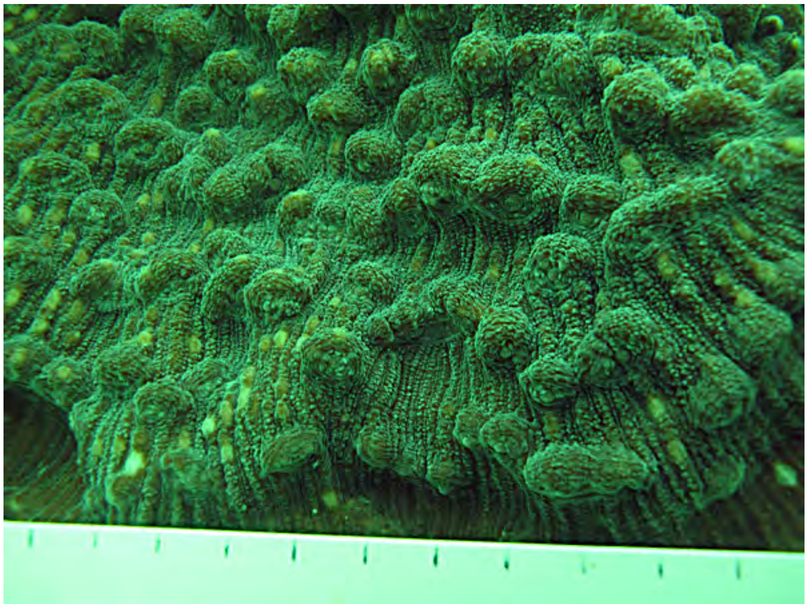
Mycedium elephantotus S121

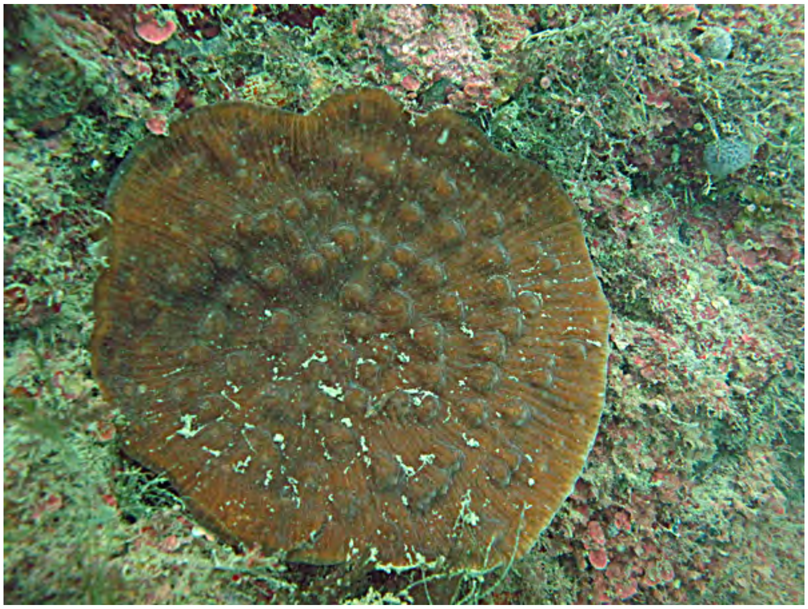

Mycedium robokaki S126

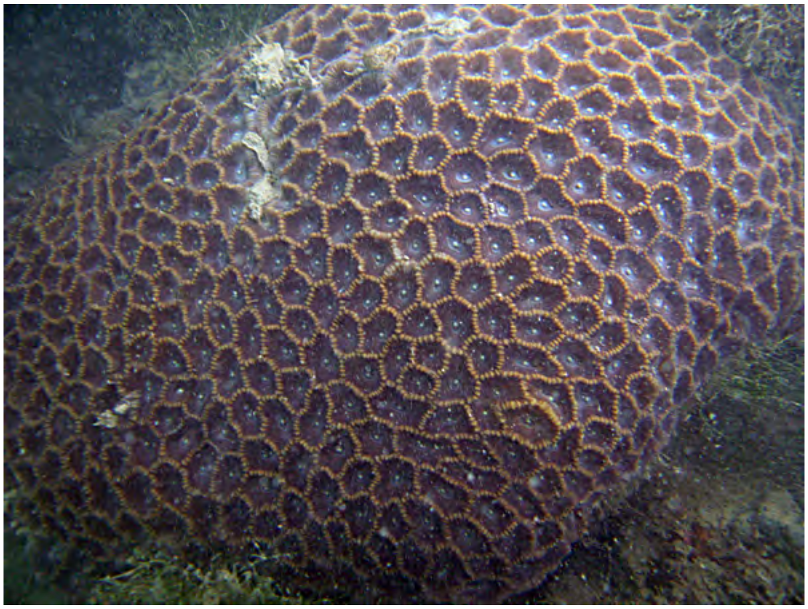

Oulophyllia bennettae S033

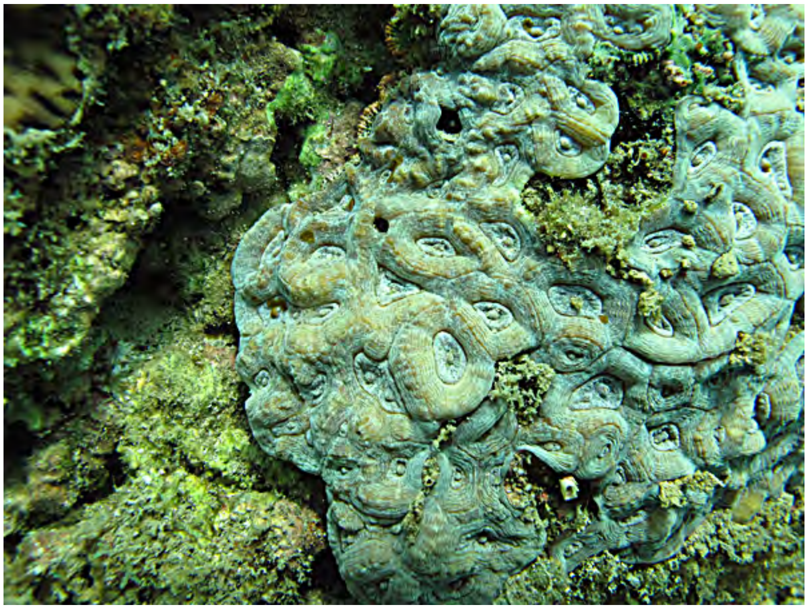

Oulophyllia cf. *bennettae* P140

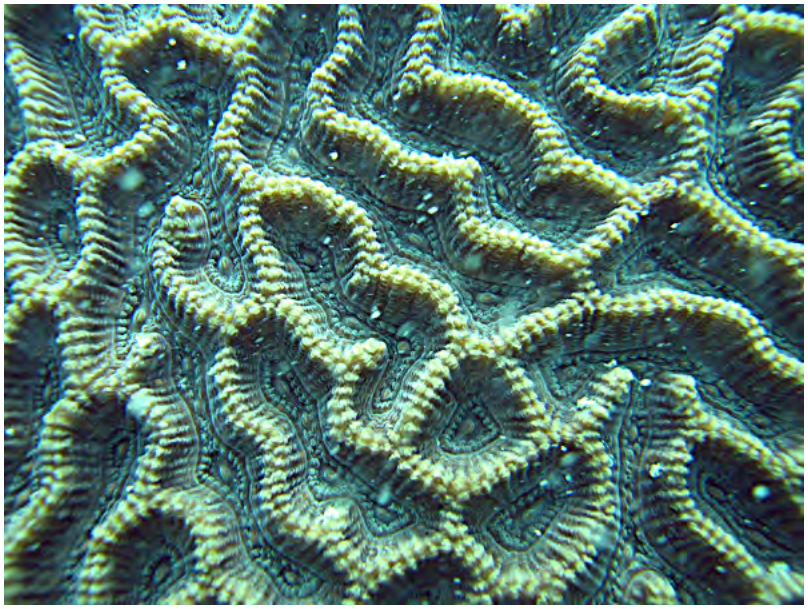

Oulophyllia crispa S055

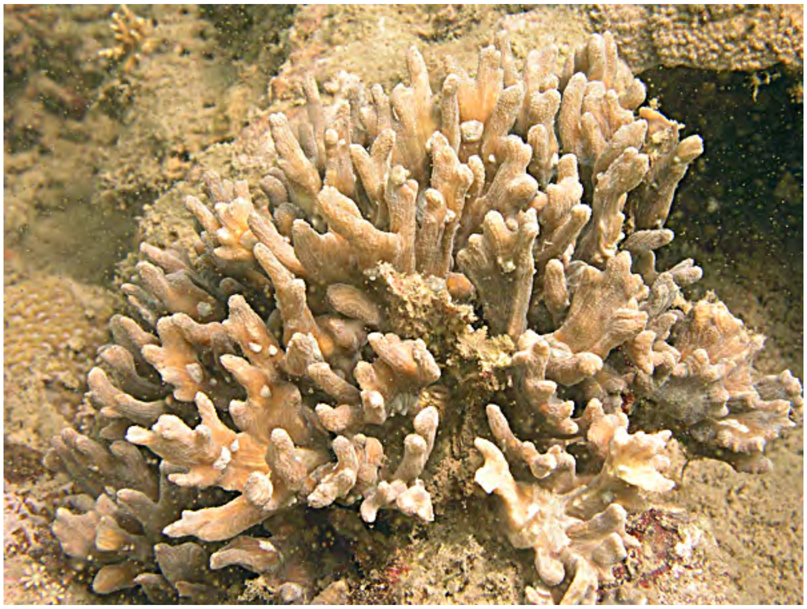

Pectinia alcicornis P124

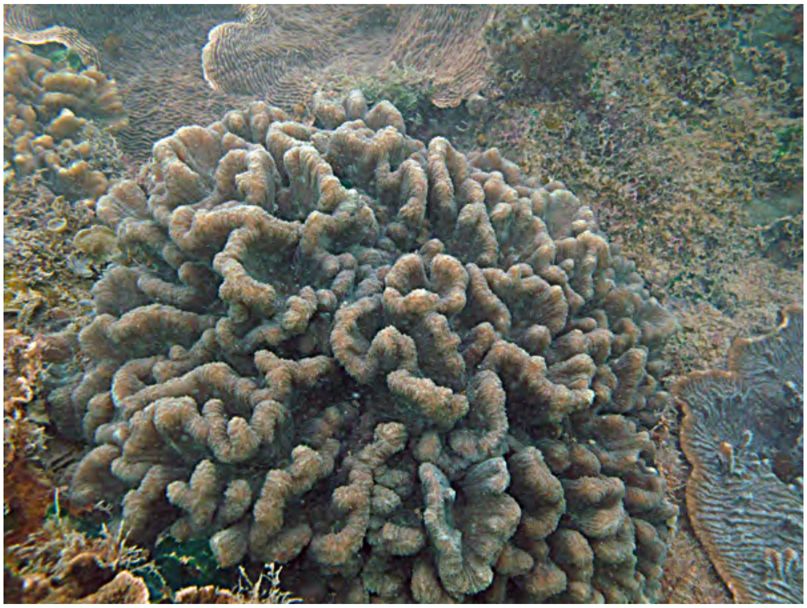

Pectinia ayleni S122

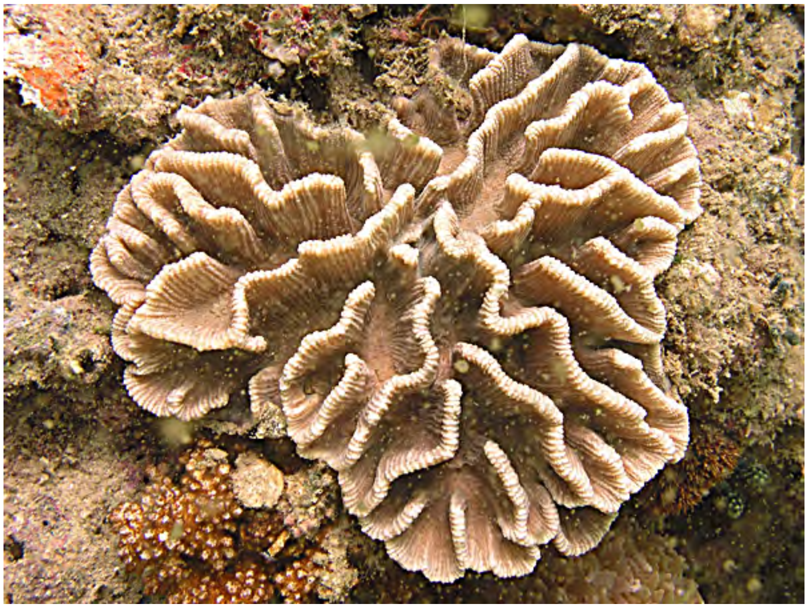
Pectinia lactuca P115

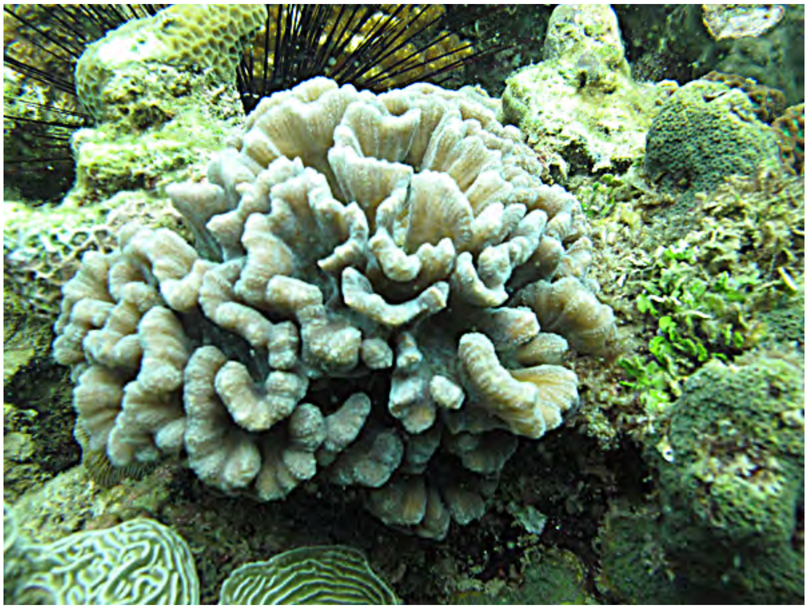
Pectinia paeonia P126

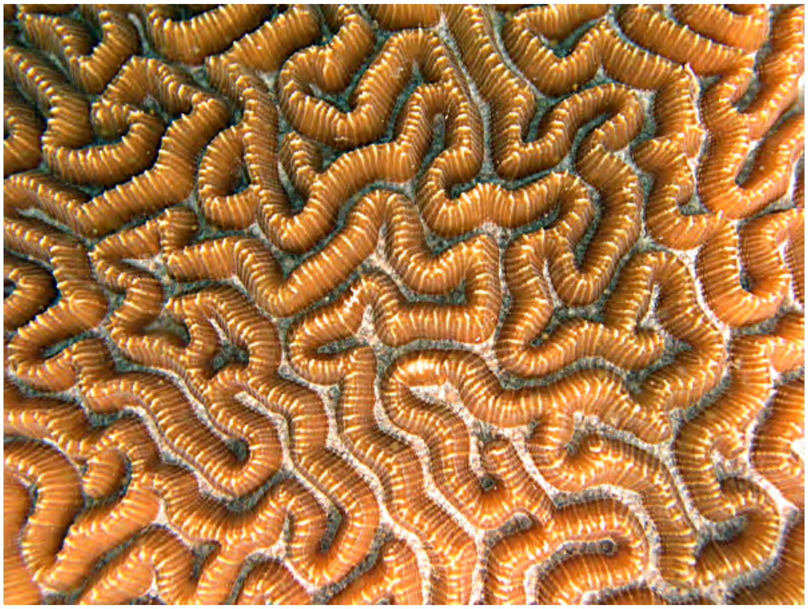
Platygyra acuta P123

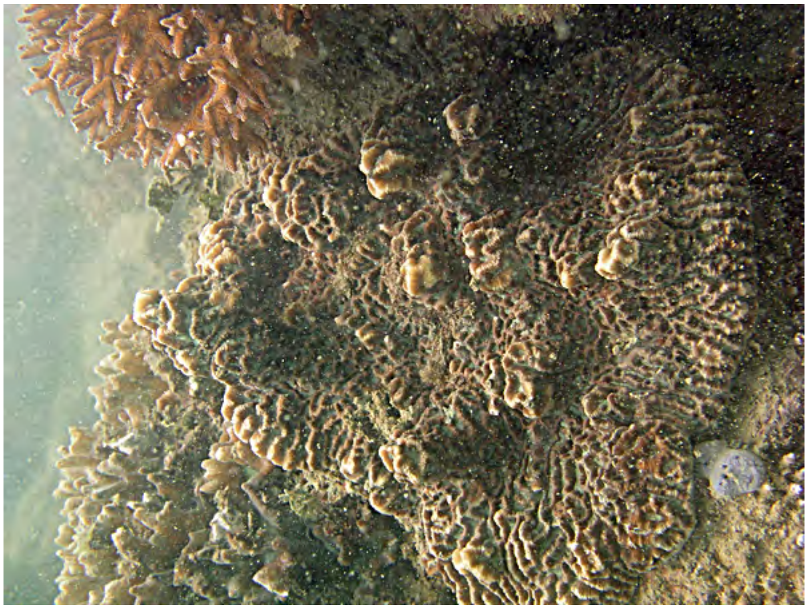
Platygyra contorta P112

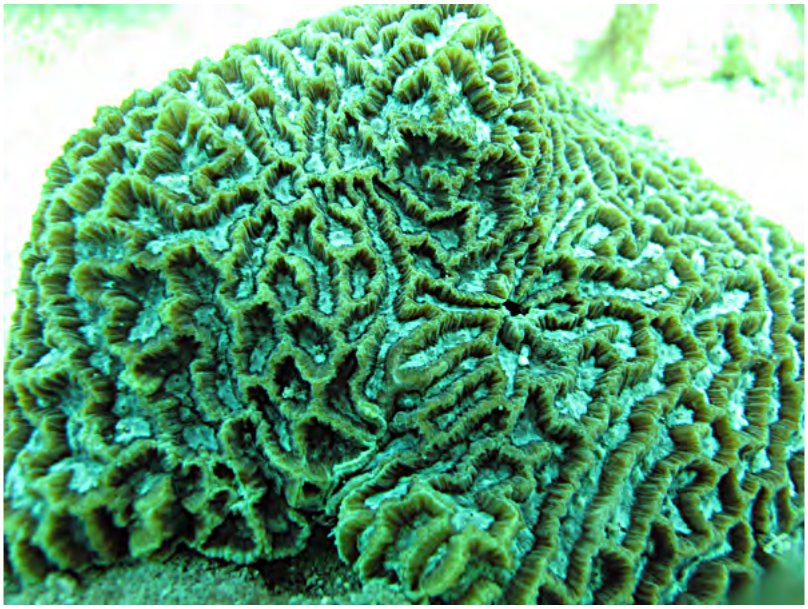
Platygyra daedalea G61878

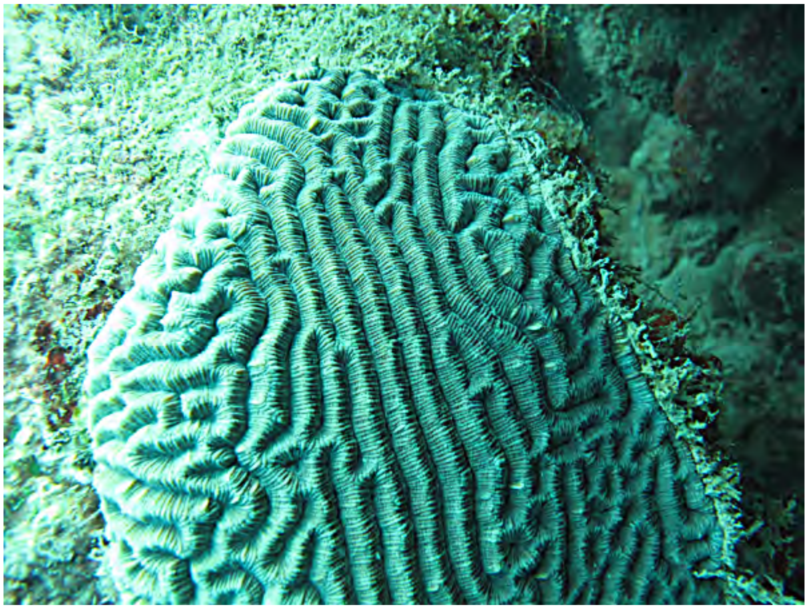
Platygyra lamellina G61887

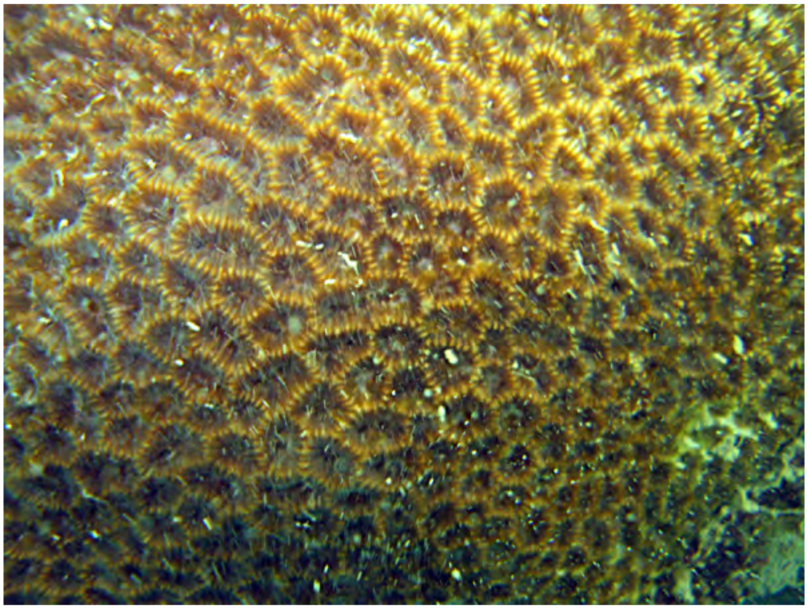

Platygyra pini S035

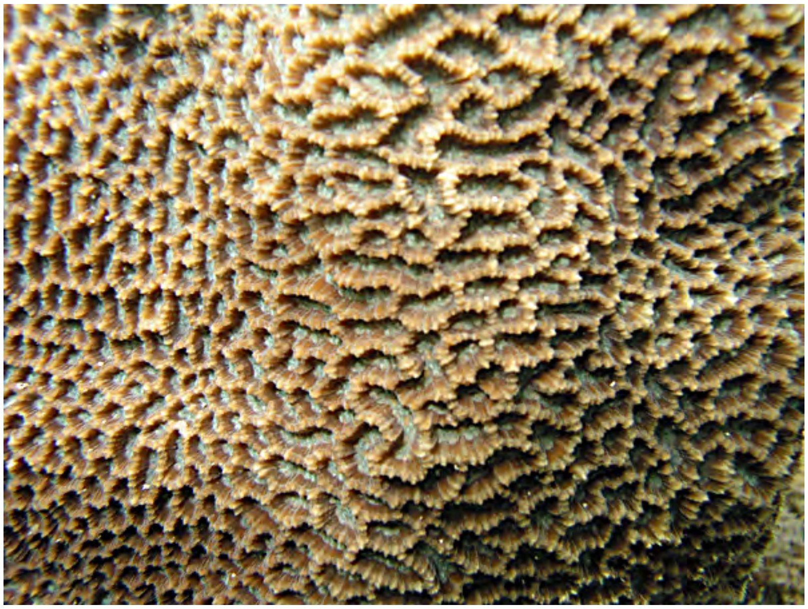

Platygyra ryukyuensis P101

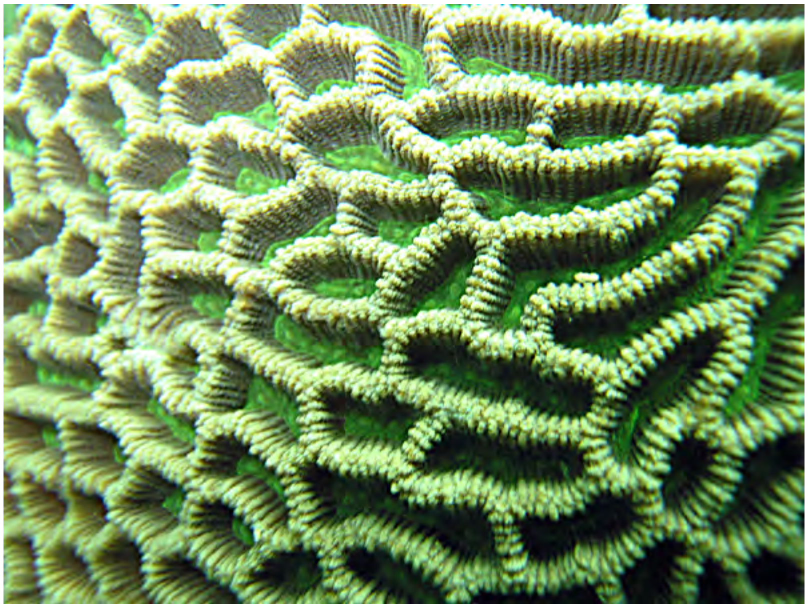

Platygyra sinensis S118

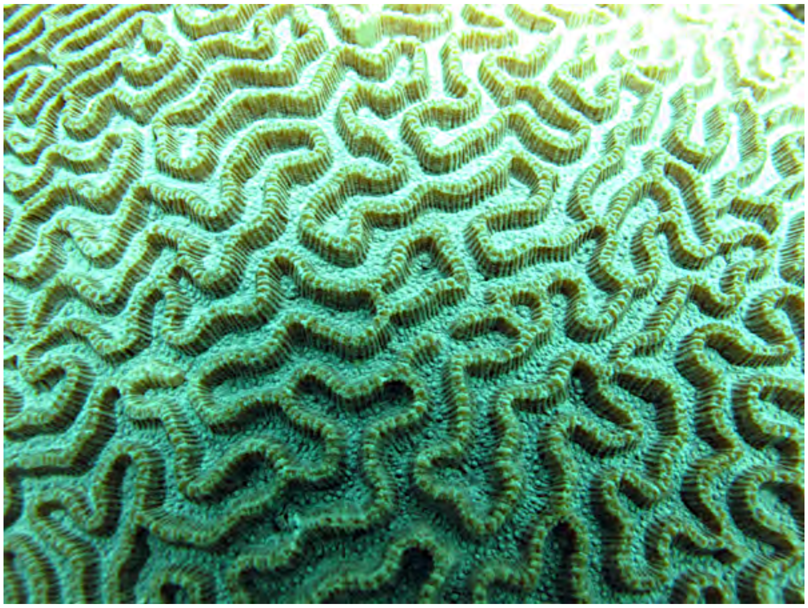

Platygyra sinensis P130

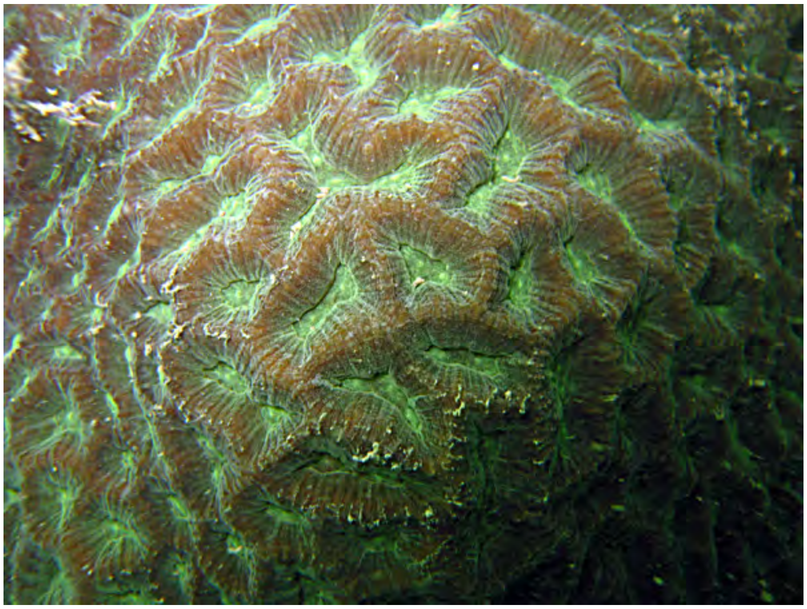

Platygyra cf. *verweyi* S037

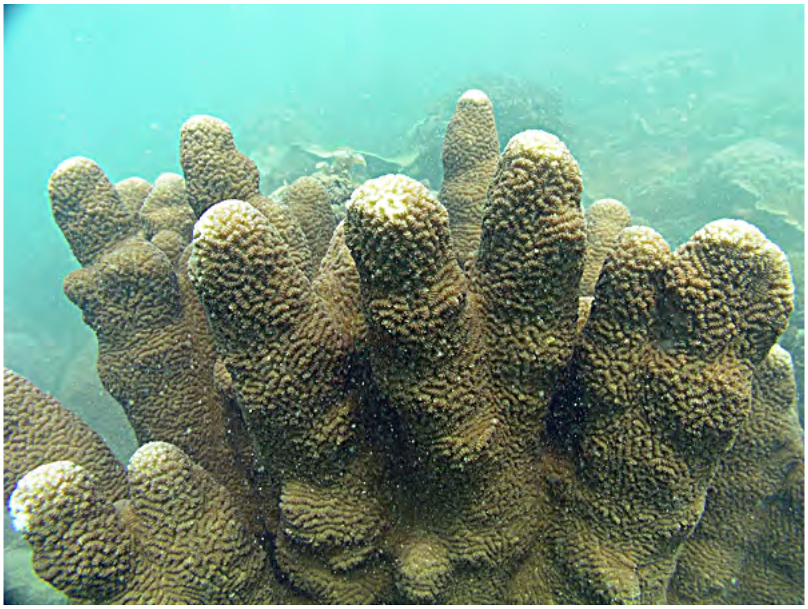

Scapophyllia cylindrica S060
