# Supplementary figures and images for: Cleaning up the 'Bigmessidae': Molecular phylogeny of scleractinian corals from Faviidae, Merulinidae, Pectiniidae and Trachyphylliidae
Source: BMC Evol Biol. 2011 Feb 7;11:37. doi: 10.1186/1471-2148-11-37 (PMC3042006; doi:10.1186/1471-2148-11-37)

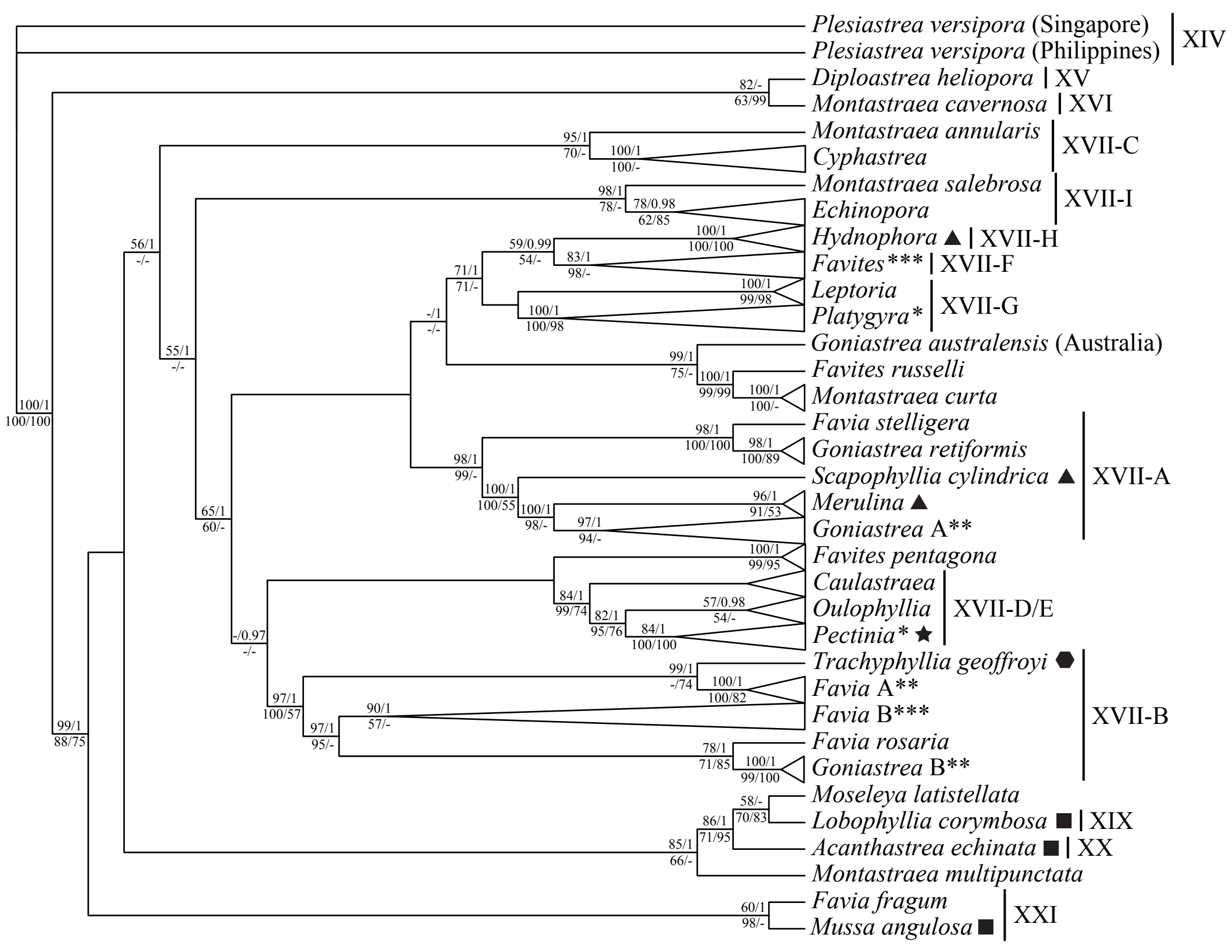

Supplement: Additional file 2 — Maximum likelihood tree topology of the combined molecular data. Numbers above branches are maximum likelihood bootstrap ≥50 and Bayesian posterior probability ≥0.9, while numbers below denote maximum parsimony bootstrap ≥50 and neighbor-joining bootstrap ≥50. Family classification follows definitions given for Figure 1. [file 1471-2148-11-37-S2.PDF]

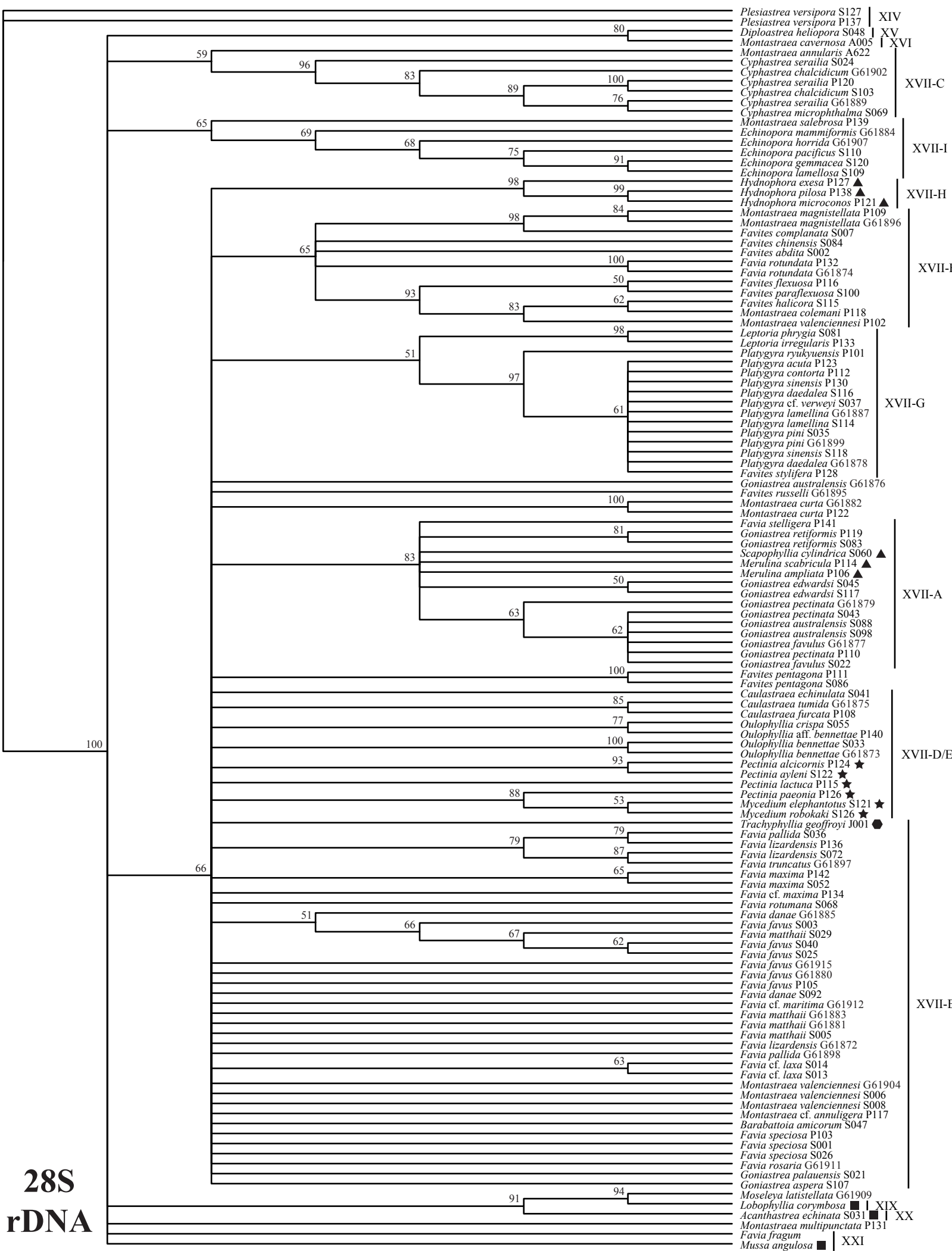

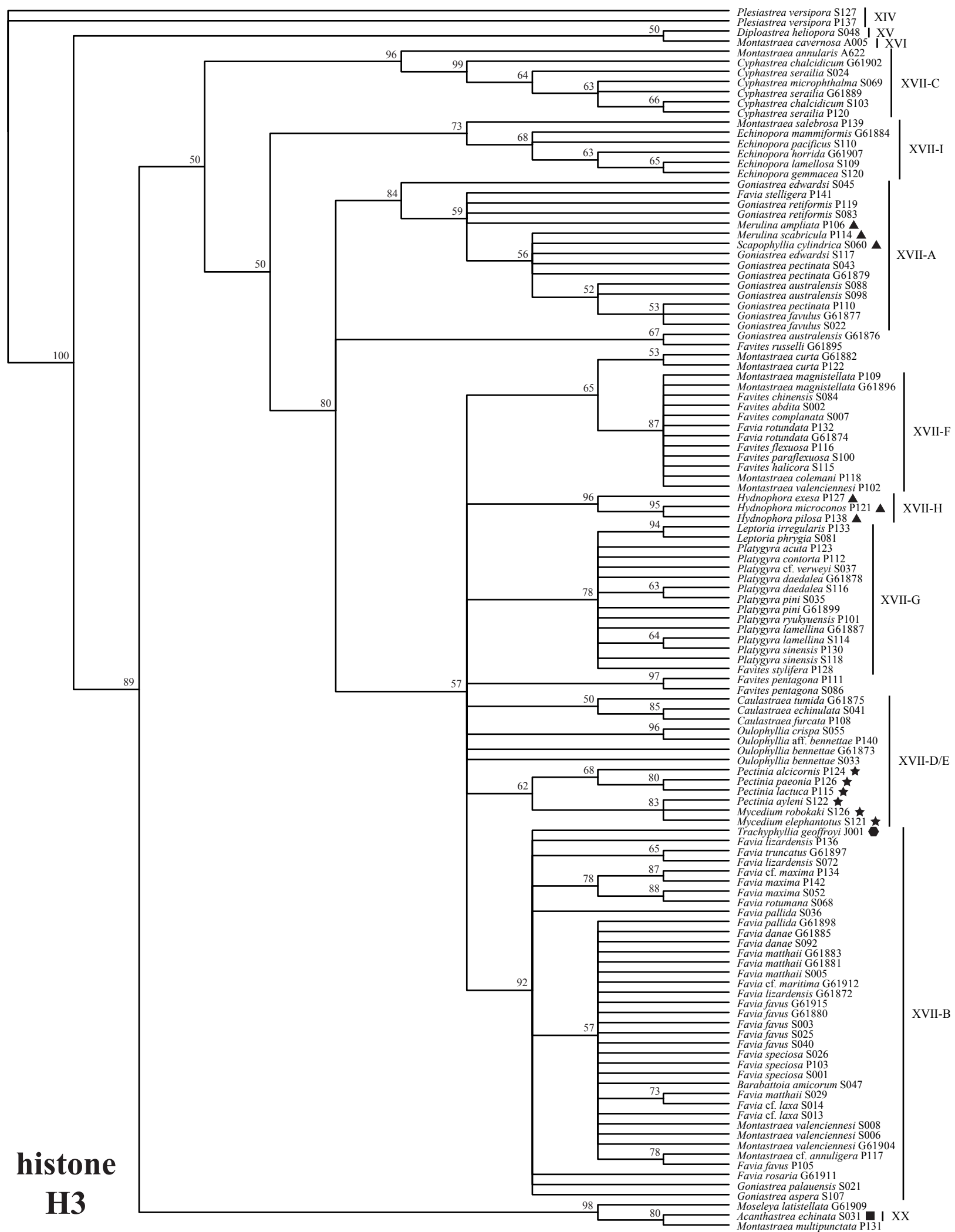

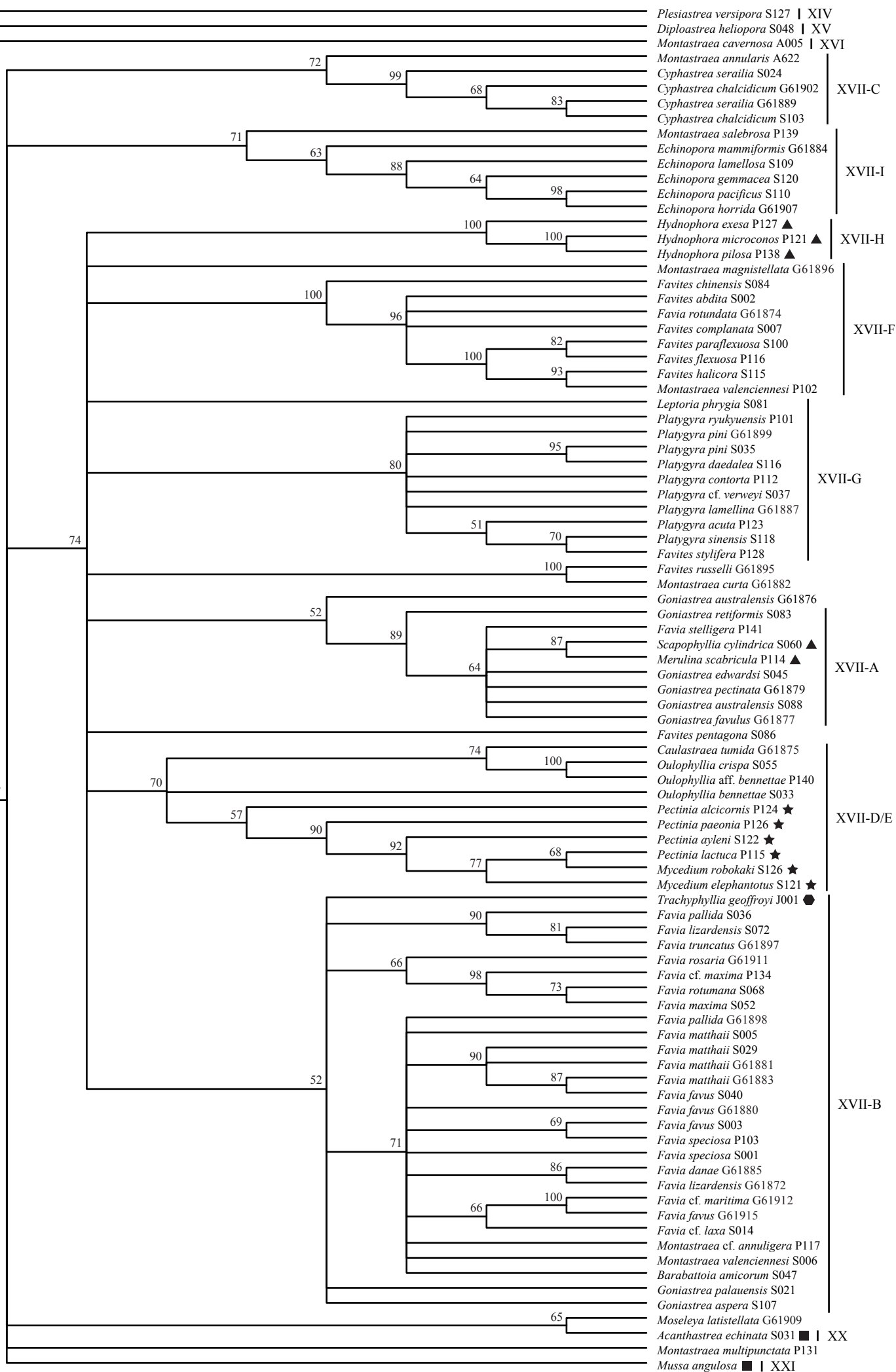

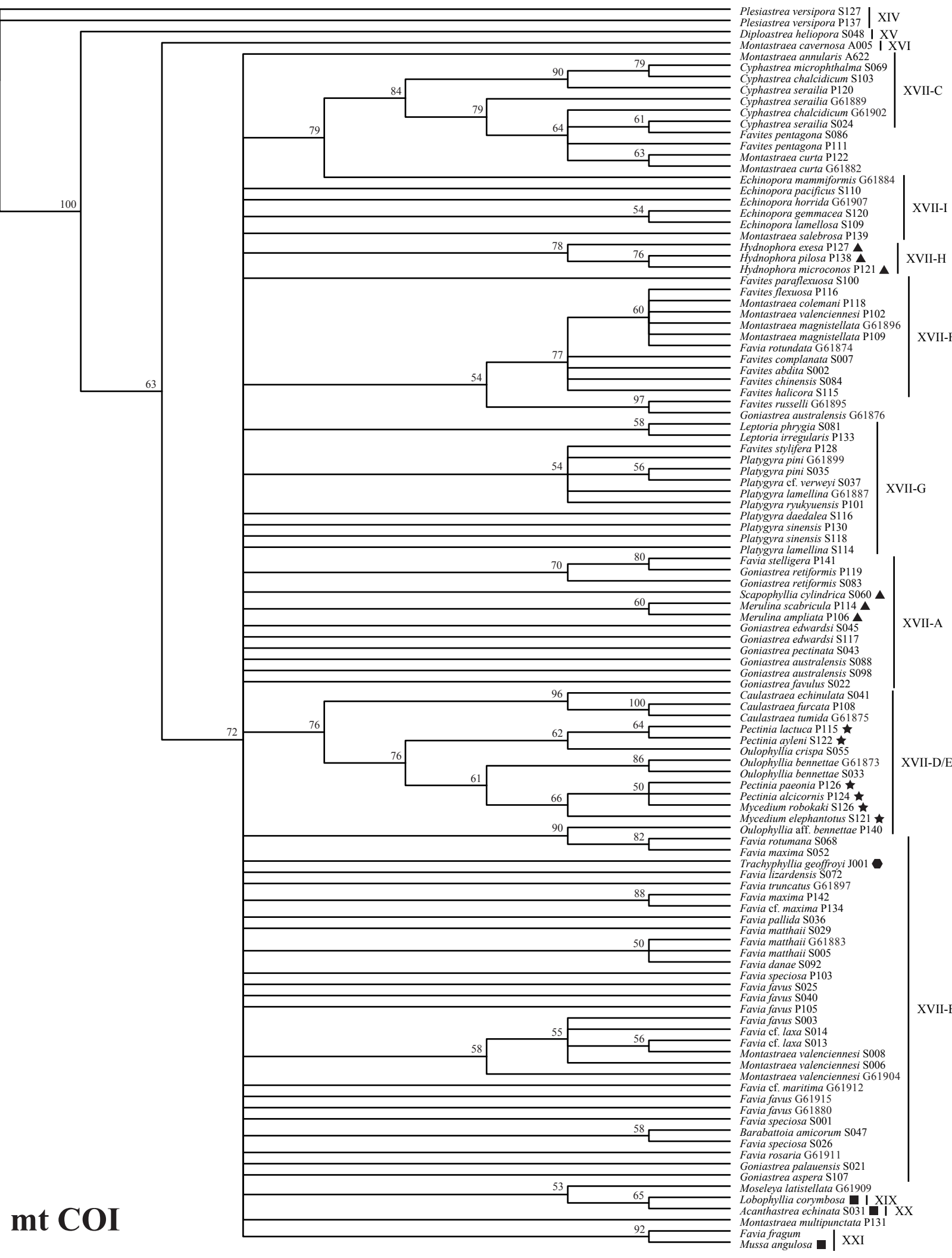

mt  
IGR

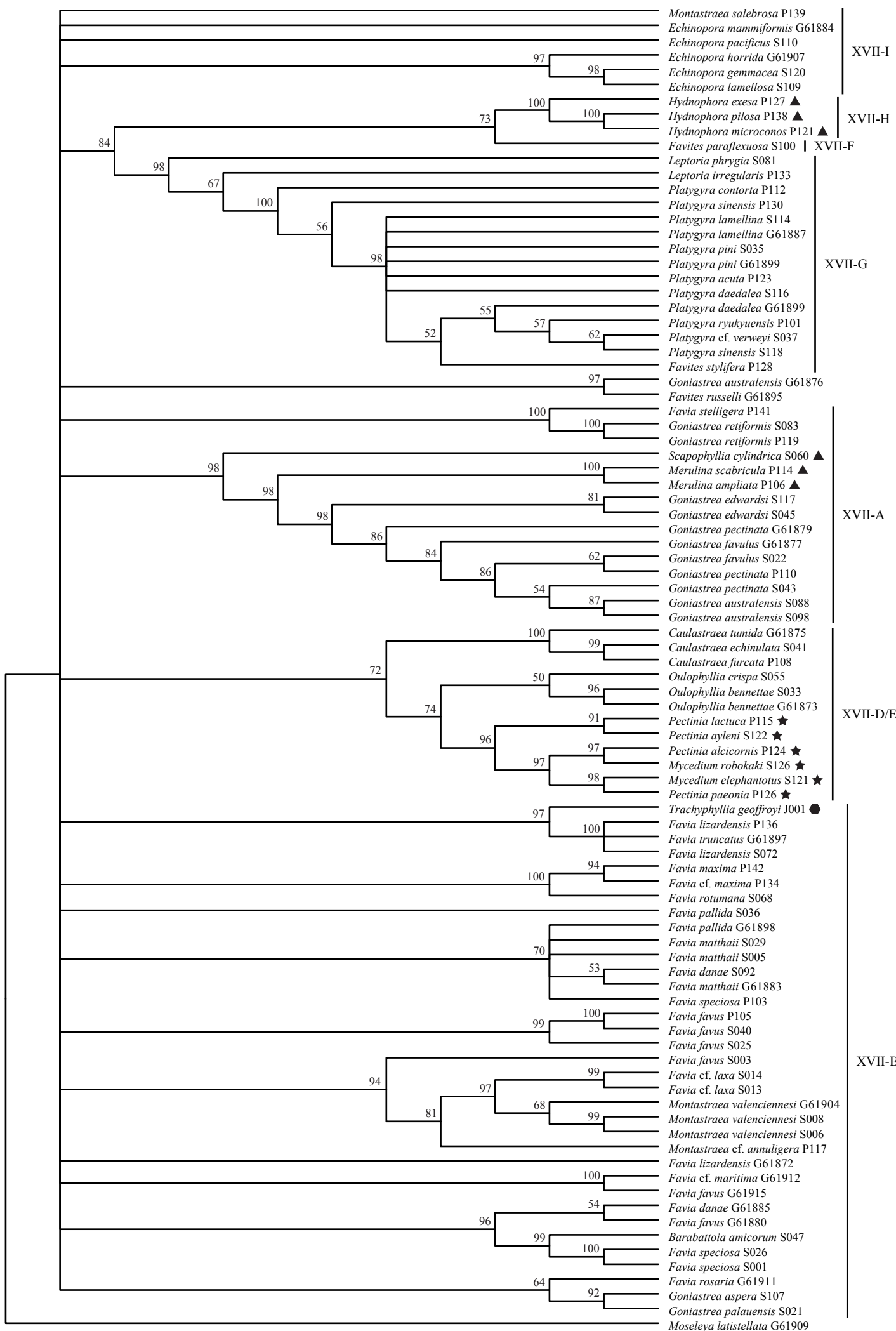

Supplement: Additional file 3 — Maximum likelihood tree topology of each partition. Numbers adjacent to branches are bootstrap support values ≥50. Definitions for family classification follow Figure 1. [file 1471-2148-11-37-S3.PDF]
